# Supplementary material for: High-accuracy crRNA array assembly strategy for multiplex CRISPR
Source: Mol Ther Nucleic Acids. 2024 Dec 12;36(1):102428. doi: 10.1016/j.omtn.2024.102428 (PMC11787013; doi:10.1016/j.omtn.2024.102428)
Supplement: Document S1. Figures S1–S24 and Tables S23–S36 [file mmc1.pdf]

OMTN, Volume *36*

Supplemental information

High-accuracy crRNA array assembly strategy for multiplex CRISPR

Xiangtong Zhao, Lixian Yang, Peng Li, Zijing Cheng, Yongshi Jia, Limin Luo, Aihong Bi, Hanchu Xiong, Haibo Zhang, Hongen Xu, Jinrui Zhang, and Yaodong Zhang

Supplemental Figures

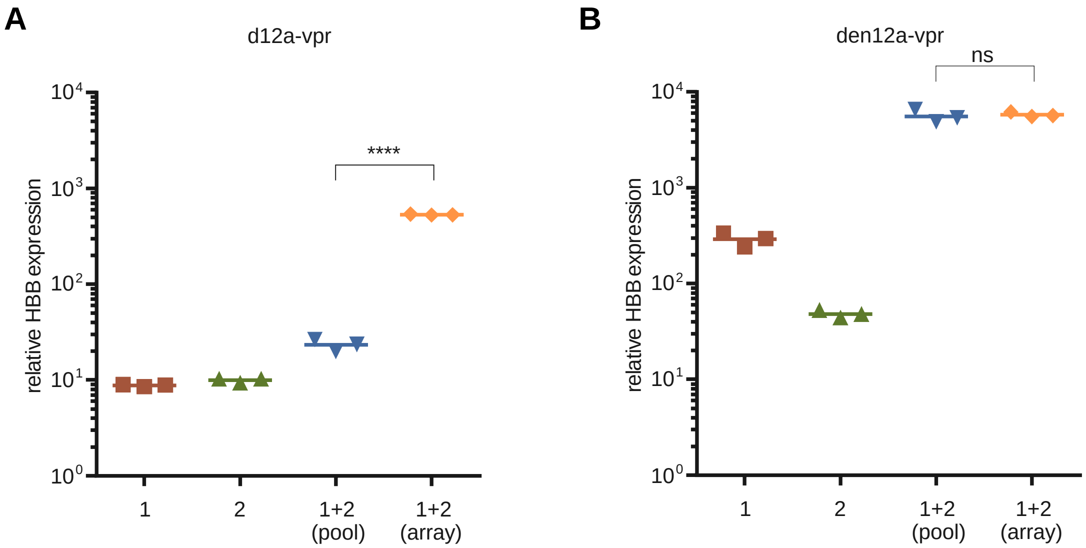

Figure S1. Synergistic transcriptional activation of *HBB* with d12a-VPR or den12a-VPR. **(A**, **B)** Quantification of relative *HBB* expression over non-targeting control in HEK293T cells 48h after transfection with plasmids encoding d12a-VPR **(A)** or den12a-VPR **(B)** and *HBB* promoter-targeting crRNA-1, crRNA-2, a pool of both, or an array containing both. Values shown as mean, n = 3 independent experiments.

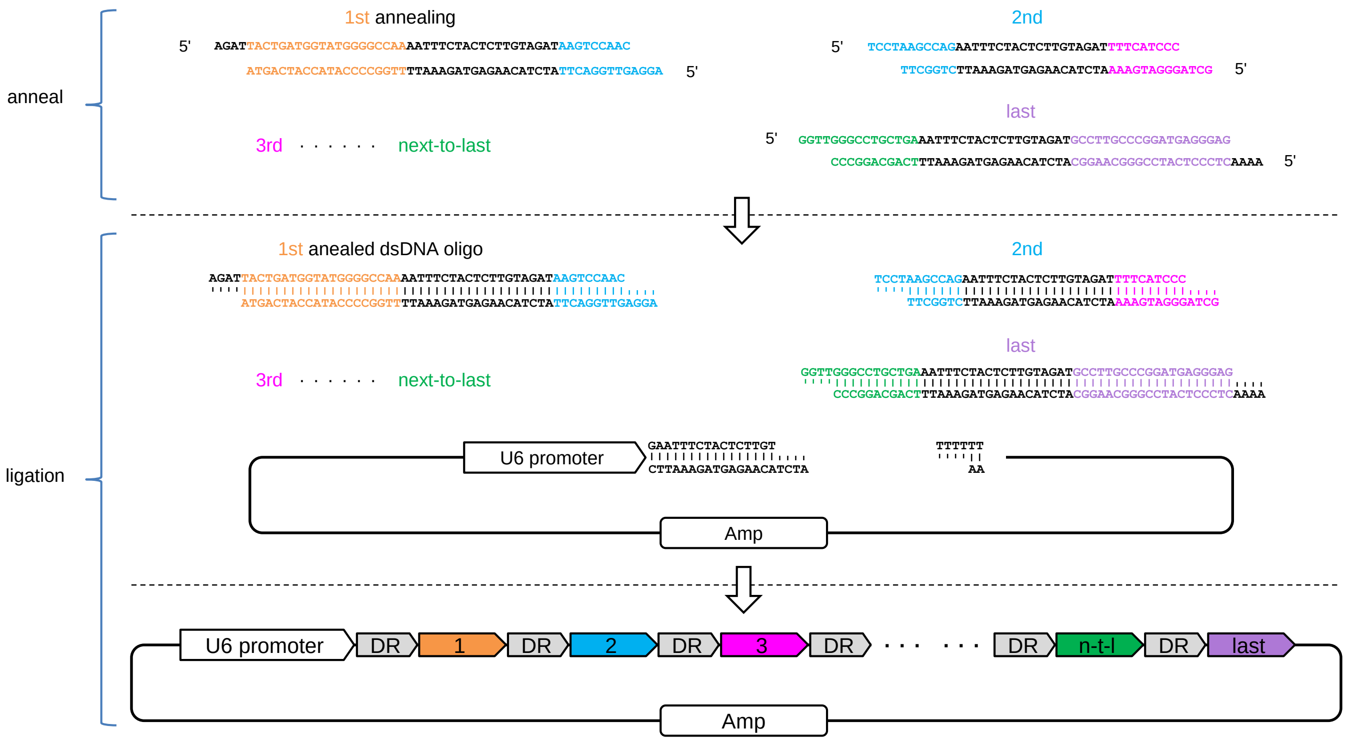

Figure S2. Schematic illustration and workflow of conventional sticky end-based CRISPR array assembly strategy. First, anneal single-stranded oligo pairs to form double-stranded DNA segments with desired sticky ends. Then, set up and run a ligation reaction with diluted annealed oligos and destination cloning vector (with/without predigestion and purification). Detailed protocols are provided in Supplemental Methods.

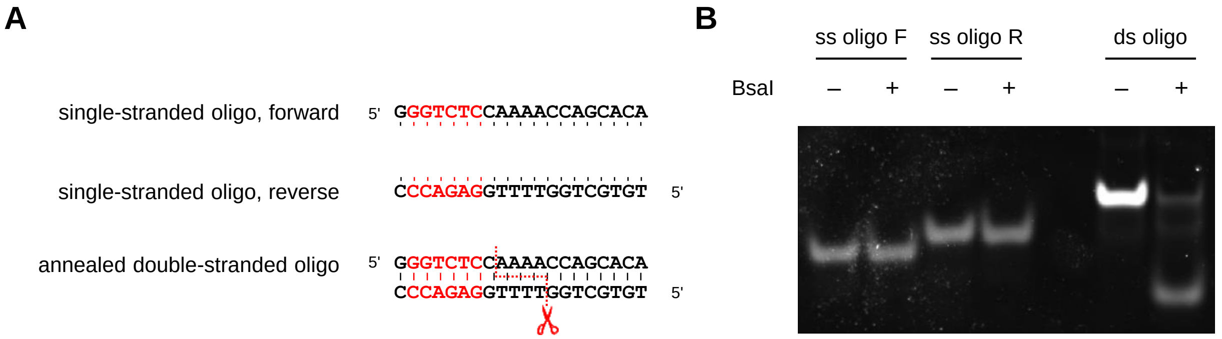

Figure S3. Restriction endonuclease BsaI specifically cleaves double-stranded DNA substrates. **(A)** Reverse complementary single-stranded oligos and resulting annealed double-stranded product. Recognition site of BsaI is colored red. Cleavage site is indicated by red dashed line. **(B)** Representative PAGE image of oligos depicted in (**A**) after incubation with/without BsaI at 37°C for 1 hour.

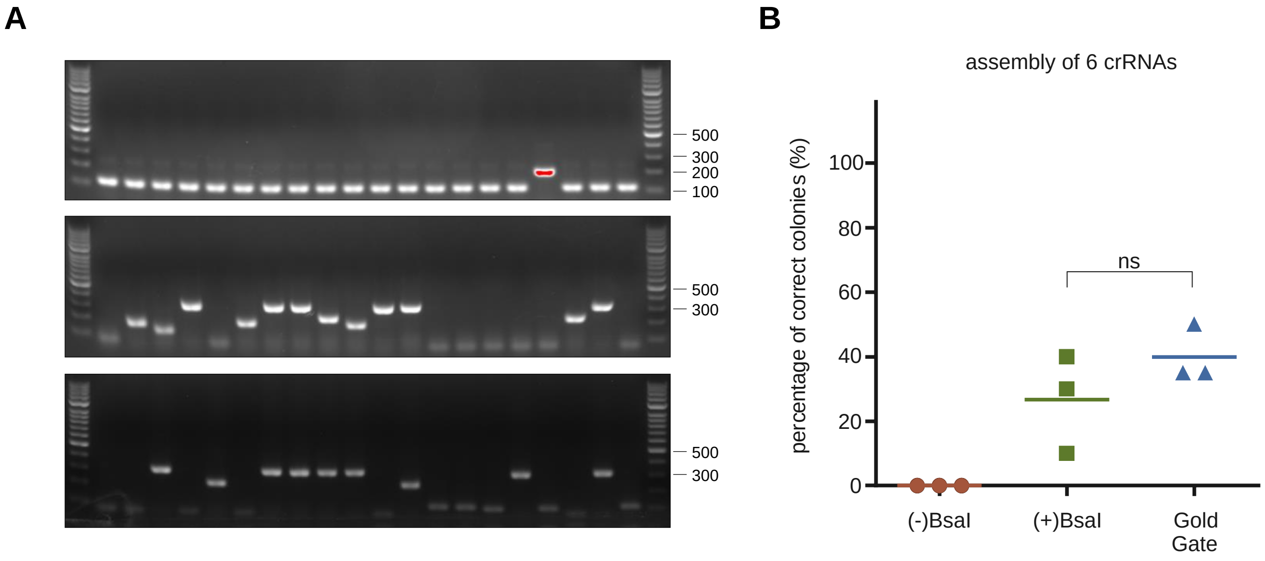

Figure S4. Comparison of assembly accuracy across different versions of conventional sticky end-based strategy. **(A)** Representative images of colony PCR to evaluate the accuracy of assembling 6 crRNAs (correct size ≈ 290 nt) using three versions of sticky end-based strategy: (-)BsaI (upper), (+)BsaI (middle), Gold Gate (lower), and the corresponding accuracies are shown in **(B)**. (-)BsaI: pre-digested cloning vector is recovered and added to a ligation reaction with annealed oligos, incubated overnight at room temperature, then directly transformed into competent cells; (+)BsaI: same as (-)BsaI, except that the ligation mixture is re-digested with BsaI for 15 minutes before transformation; Gold Gate: circular cloning vector and annealed oligos are added to a Gold Gate reaction, and then run a standard Gold Gate assembly program (37℃ 5 min → 16℃ 5 min, 30 cycles, followed by 60℃ 5 min) with a thermocycler. Detailed protocols are provided in Supplemental Methods. Values shown as mean, n = 3 independent experiments.

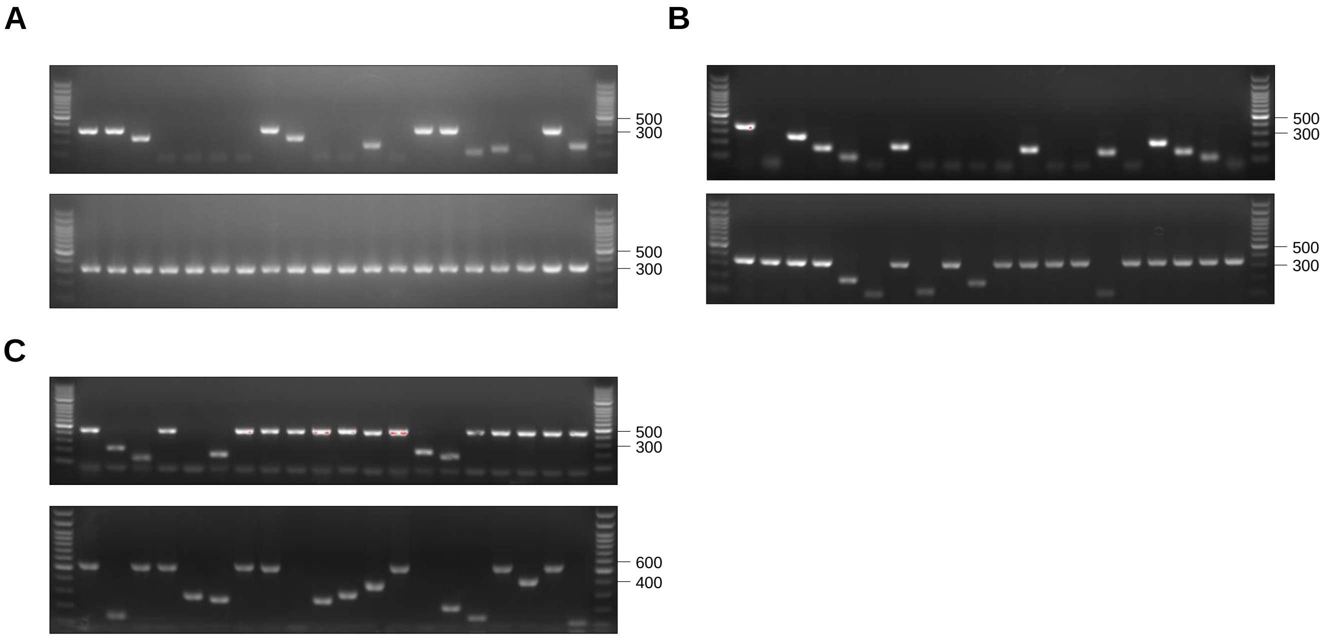

Figure S5. High-accuracy assembly of CRISPR array with GGA-based strategy. (**A**, **B**) Representative images of colony PCR to evaluate the accuracy of assembling 6 (**A**, correct size ≈ 290 nt) or 7 (**B**, correct size ≈ 330 nt) crRNAs using conventional sticky end-based (upper) or GGA-based strategy (lower). (**C**) Representative images of colony PCR to evaluate the accuracy of assembling 9 (upper, correct size ≈ 410 nt) or 12 (lower, correct size ≈ 530 nt) crRNAs using GGA-based strategy.

A **B**

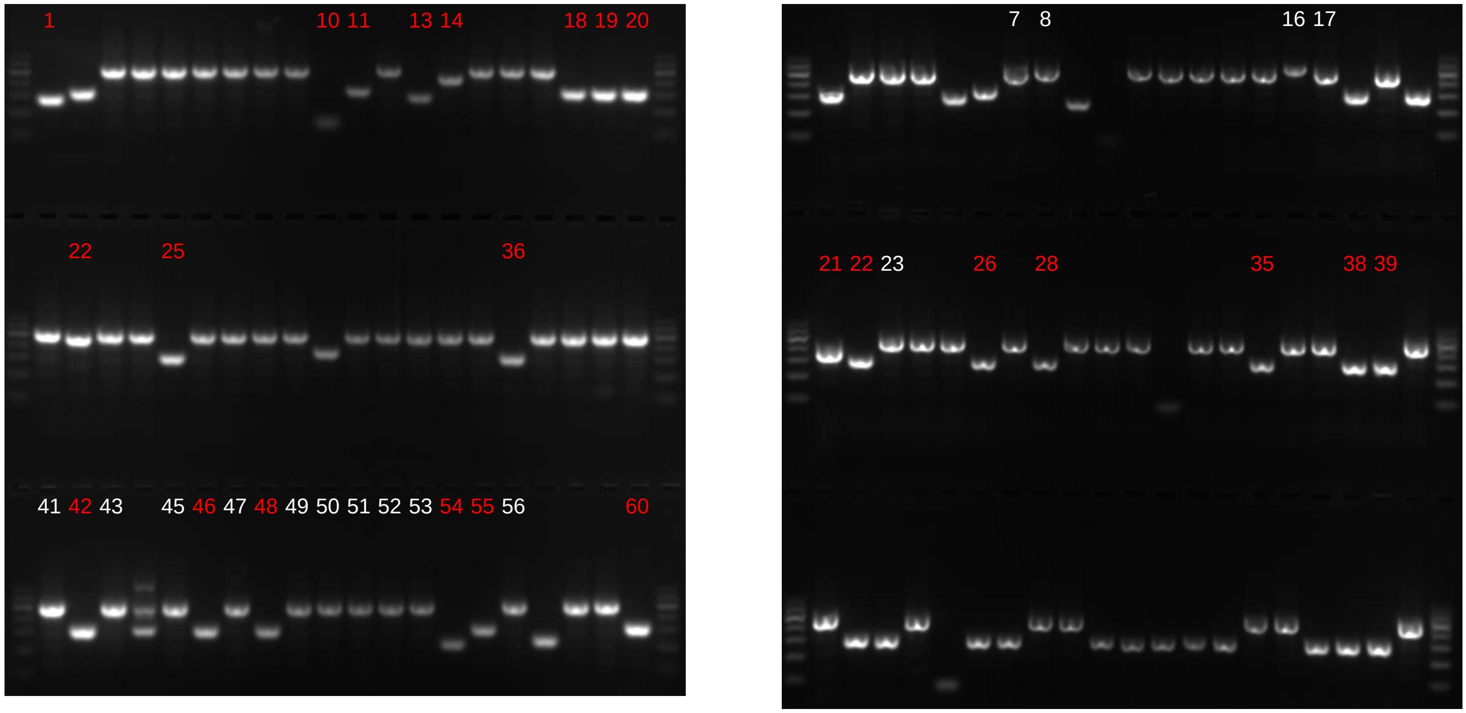

Figure S6. For the assembly of the 12 crRNAs, colonies from an additional replicate experiment were sequenced to confirm our results and investigate the source of errors in incorrect inserts during the revision of this manuscript. The 12×crRNA array presented here was based on the oligos used to assemble the reverse array shown in Figure S14A, as these oligos are closer to the manufacturing date. Note that these oligos were designed based on the mutant DR sequence (Figure 3C).

Colonies numbered with a white front indicated sequenced correctly sized colonies. All colonies except colony A-43 were sequenced to be correct (14/15). The sanger sequencing results in FASTA format were provided in the Table S37.

Colonies numbered with a red front indicated sequenced incorrectly sized colonies. Due to our limited capacity, we were barely able to find any clues to further mechanisms from the sequencing results, except that 15 of the 24 incorrectly sized colonies had the same wrong sequence: A (11, 18, 20, 25, 36, 42, 46, 55, 60), B (22, 26, 28, 35, 38, 39). And the sequence is: AATTCCTACTCTTGTAGGTGCCTTGCCCGGATGAGGGAGAATTCCTACTCTTGTAGGTCTGTGGG-TTGGGCCTGCTGAAATTCCTACTCTTGTAGGTACATCAGCAACCAATGCTCTAATTCCTACTCTTGTAGGTTA TGACTGCCCACAAGTGCTAATTCCTACTCTTGTAGGTAATGAATGTGTGCCAGCCAATTCCTACTCTTGTAGG TAAGTCCAACTCCTAAGCCAGAATTCCTACTCTTGTAGGTTACTGATGGTATGGGGCCAA. The error site had been annotated as red underlined letters. It was an 18-nt hybrid of the following two 20-nt spacers: AATGAATGT**GTGC**ACACATG, TTCTTCATCCC**TAGC**CAGCC. The sanger sequencing results in FASTA format were provided in the Table S38.

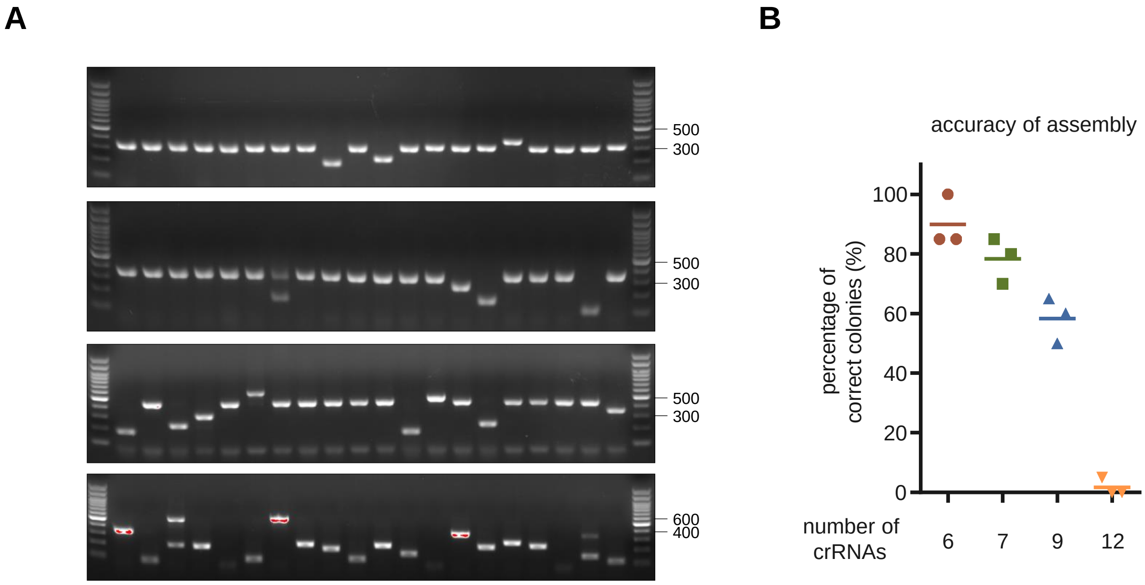

Figure S7. Accuracies of assembling 6 to 12 crRNAs using simplified recovery-free version of GGA-based strategy. (**A**) Representative images of colony PCR when assembling 6 (upper, correct size ≈ 290 nt), 7 (middle-upper, correct size ≈ 330 nt), 9 (middle-lower, correct size ≈ 410 nt), and 12 (lower, correct size ≈ 530 nt) crRNAs, and the corresponding accuracies are shown in **(B)**. Values shown as mean with n = 3.

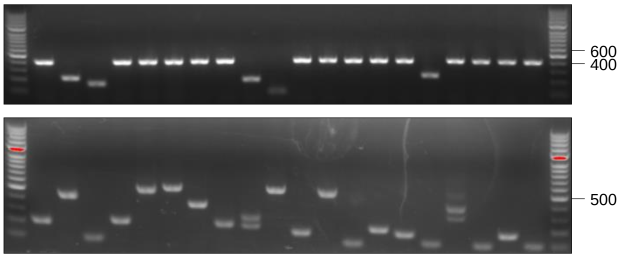

Figure S8. Representative images of colony PCR when assembling 9 (upper, correct size ≈ 410 nt) and 12 (lower, correct size ≈ 530 nt) crRNAs using mutant DR with higher GC content.

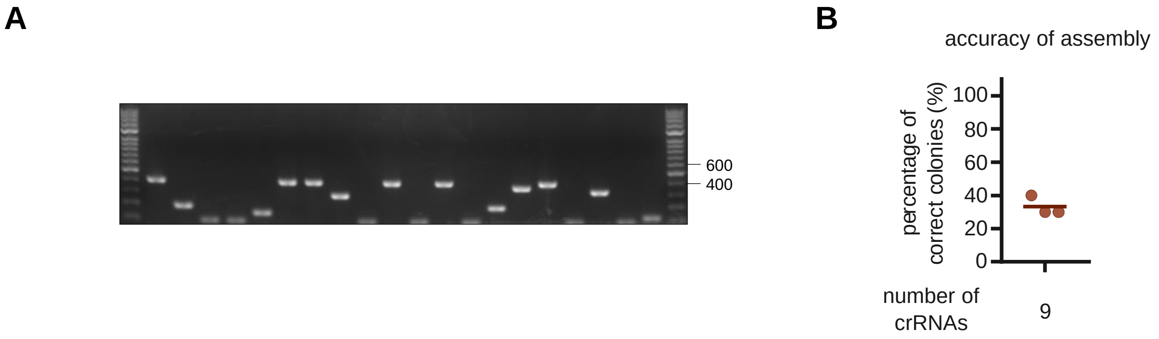

Figure S9. Accuracies of assembling 9 crRNAs (correct size ≈ 410 nt) using simplified recovery-free version of GGA-based strategy and mutant DR with higher GC content. (**A**) Representative image of colony PCR, and the corresponding accuracies are shown in **(B)**. Values shown as mean with n = 3.

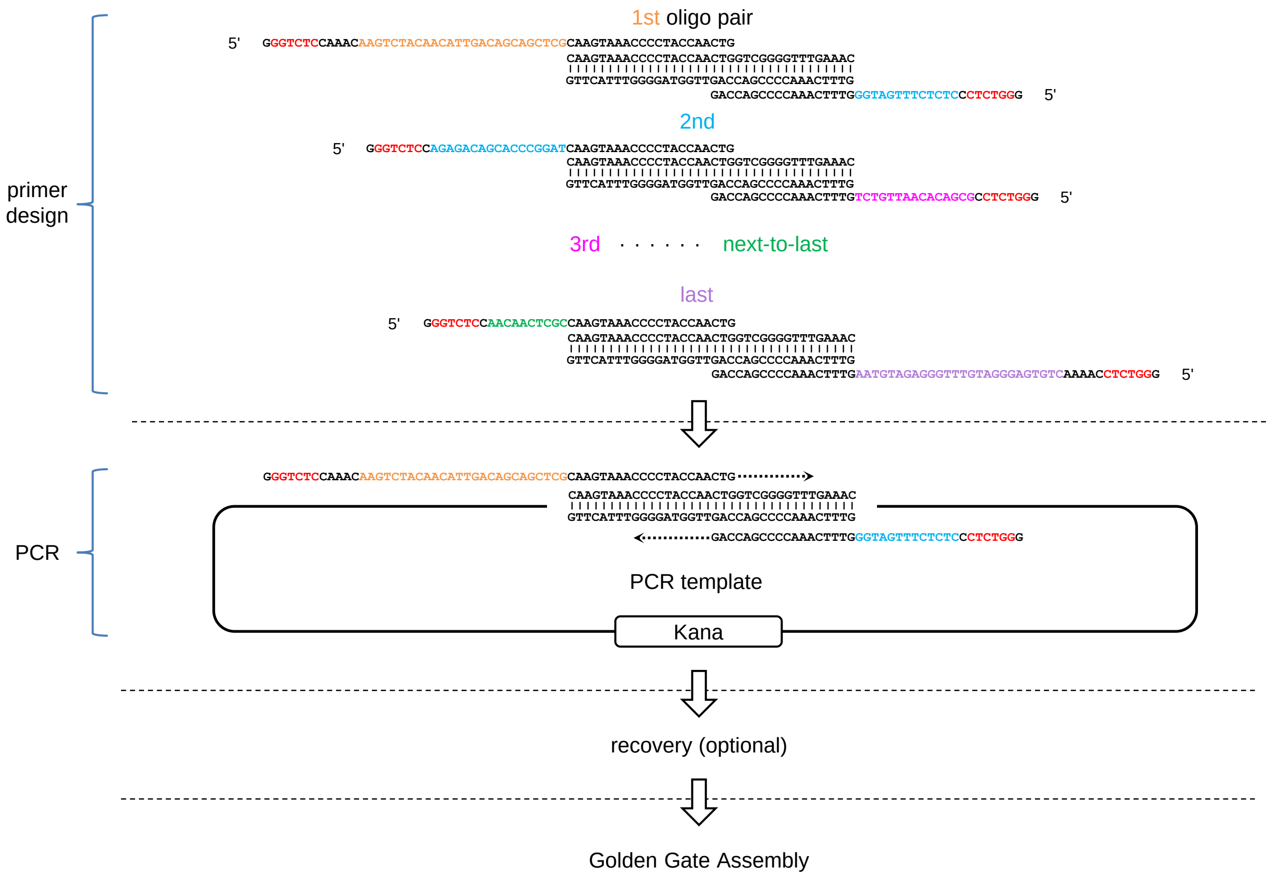

Figure S10. Schematic illustration and workflow of assembling crRNAs for RfxCas13d. Similar to Figure 1 except for the following minor modifications: (1) Primer pairs are designed to be partially (~20 bases 3' section) complementary to the 36bp DR of RfxCas13d, instead of to each other; (2) To further shorten the initial oligos for cost-saving, a kanamycin-resistant plasmid containing the 36nt DR of RfxCas13d, which could be easily home-made, is introduced as a template for PCR reactions; (3) The relatively long length of the PCR products (70~100nt) renders them compatible with common commercial DNA recovery kits, which are therefore used to obtain purer dsDNA segments.

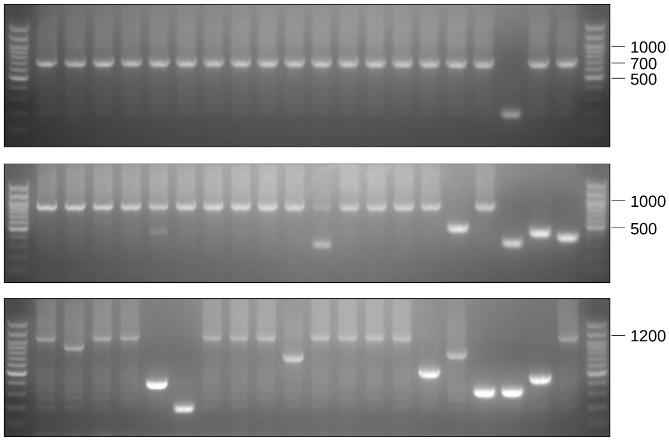

Figure S11. High-accuracy assembly of crRNAs for a Cas13d nuclease. Representative images of colony PCR to evaluate the accuracy of assembling 9 (upper, correct size ≈ 690 nt), 12 (middle, correct size ≈ 870 nt), or 15 (lower, correct size ≈ 1060 nt) crRNAs for RfxCas13d using GGA-based strategy.

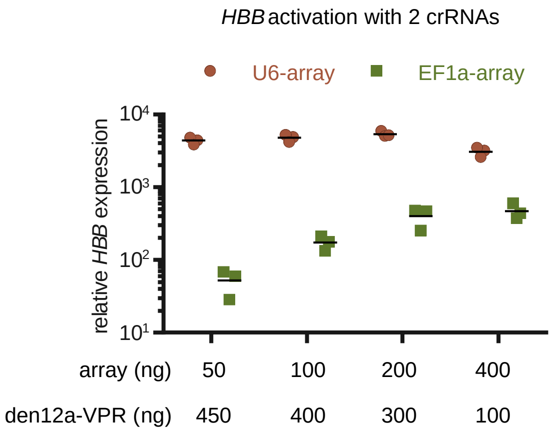

Figure S12. Quantification of relative *HBB* expression over non-targeting control in HEK293T cells 48h after transfection with the indicated amount of denAsCas12a-VPR and an array of 2 crRNAs targeting *HBB* promoter. Values shown as mean with n = 3.

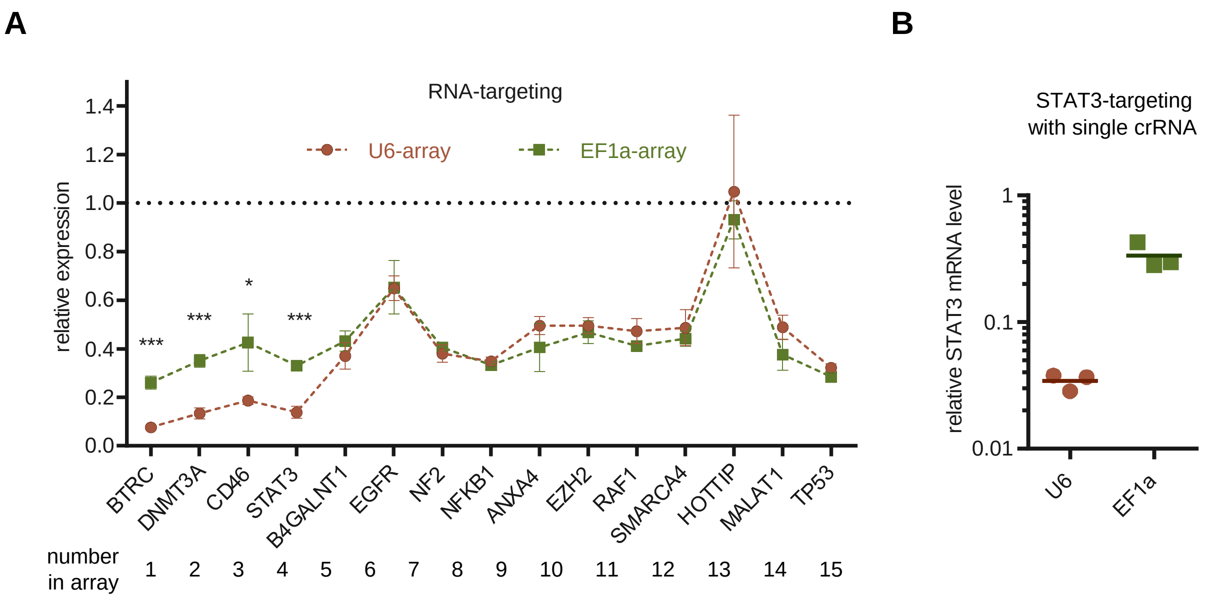

Figure S13. Distinct expression patterns of Pol II/III promoter-driven CRISPR arrays with *Rfx*Cas13d system. (**A**) Quantification of relative mRNA expression of the indicated genes compared to non-targeting control in HEK293T cells 48h after transfection with RfxCas13d and an array of 15 crRNAs driven by either U6 (U6-array) or EF1a (EF1a-array). Values shown as mean ± SD with n = 3. (**B**) Quantification of relative STAT3 mRNA expression compared to non-targeting control in HEK293T cells 48h after transfection with RfxCas13d and a STAT3-targeting crRNA driven by either U6 or EF1a. Values shown as mean with n = 3.

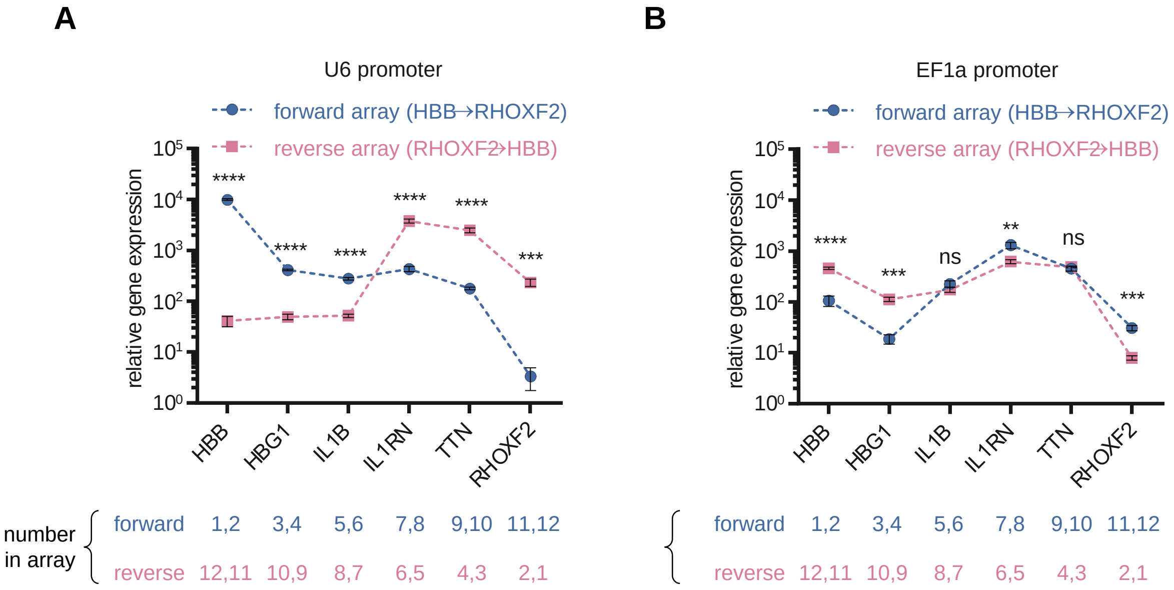

Figure S14. Transcription intensities across crRNAs located at different sites are milder but more evenly distributed in CRISPR arrays driven by Pol II promoters. (**A**) Quantification of relative mRNA expression over non-targeting control in HEK293T cells 48h after transfection with den12a-VPR and U6-driven arrays whose internal crRNAs are assembled in reverse order. (**B**) Quantification of relative mRNA expression over non-targeting control in HEK293T cells 48h after transfection with den12a-VPR and EF1a-driven arrays whose internal crRNAs are assembled in reverse order. Values shown as mean ± SD with n = 3.

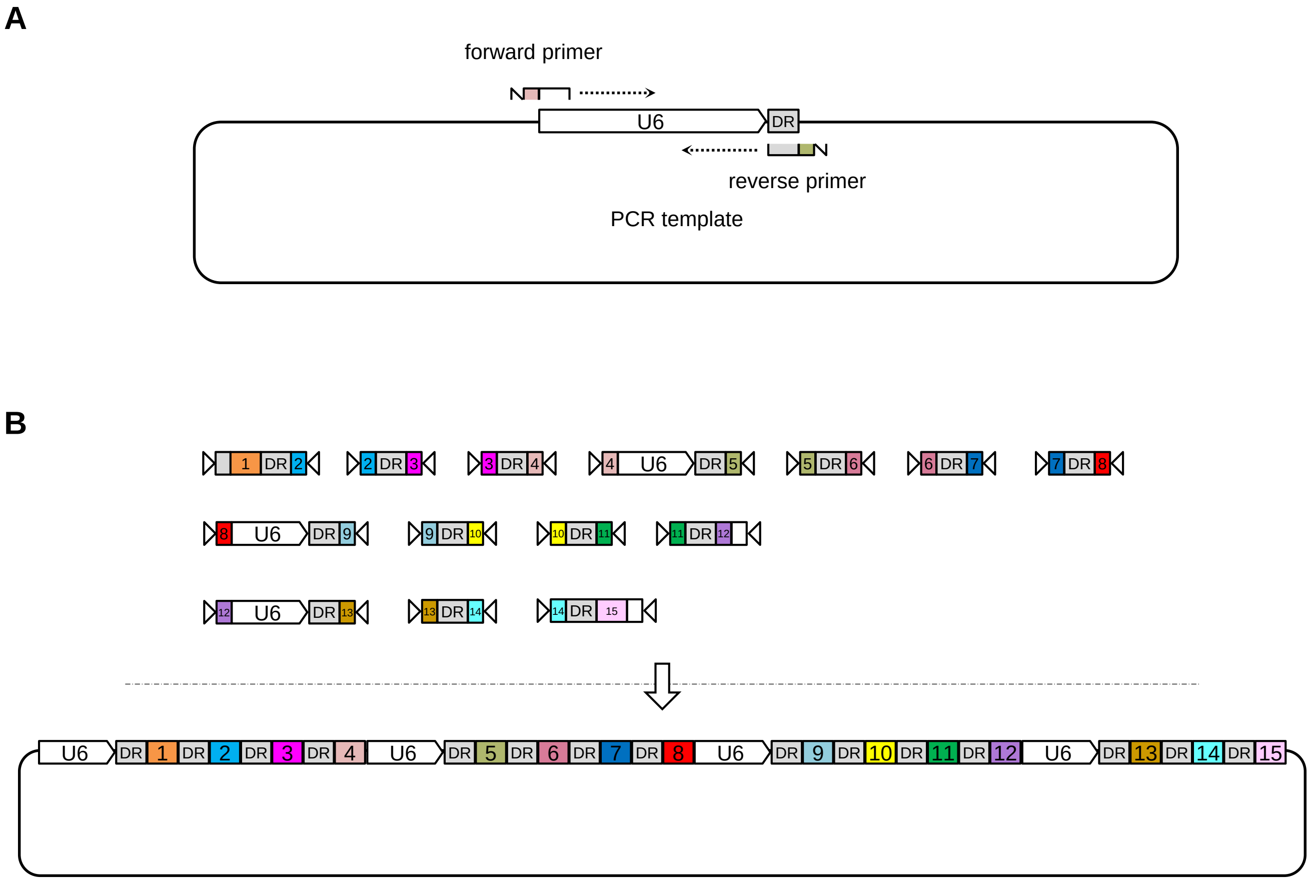

Figure S15. Assumption for the strategy to assemble a hierarchical CRISPR array. (**A**) Schematic illustration of the amplification of U6-containing fragments using a template containing a U6-DR cassette. (**B**) Schematic illustration of the proto assumption to assemble hierarchical CRISPR array.

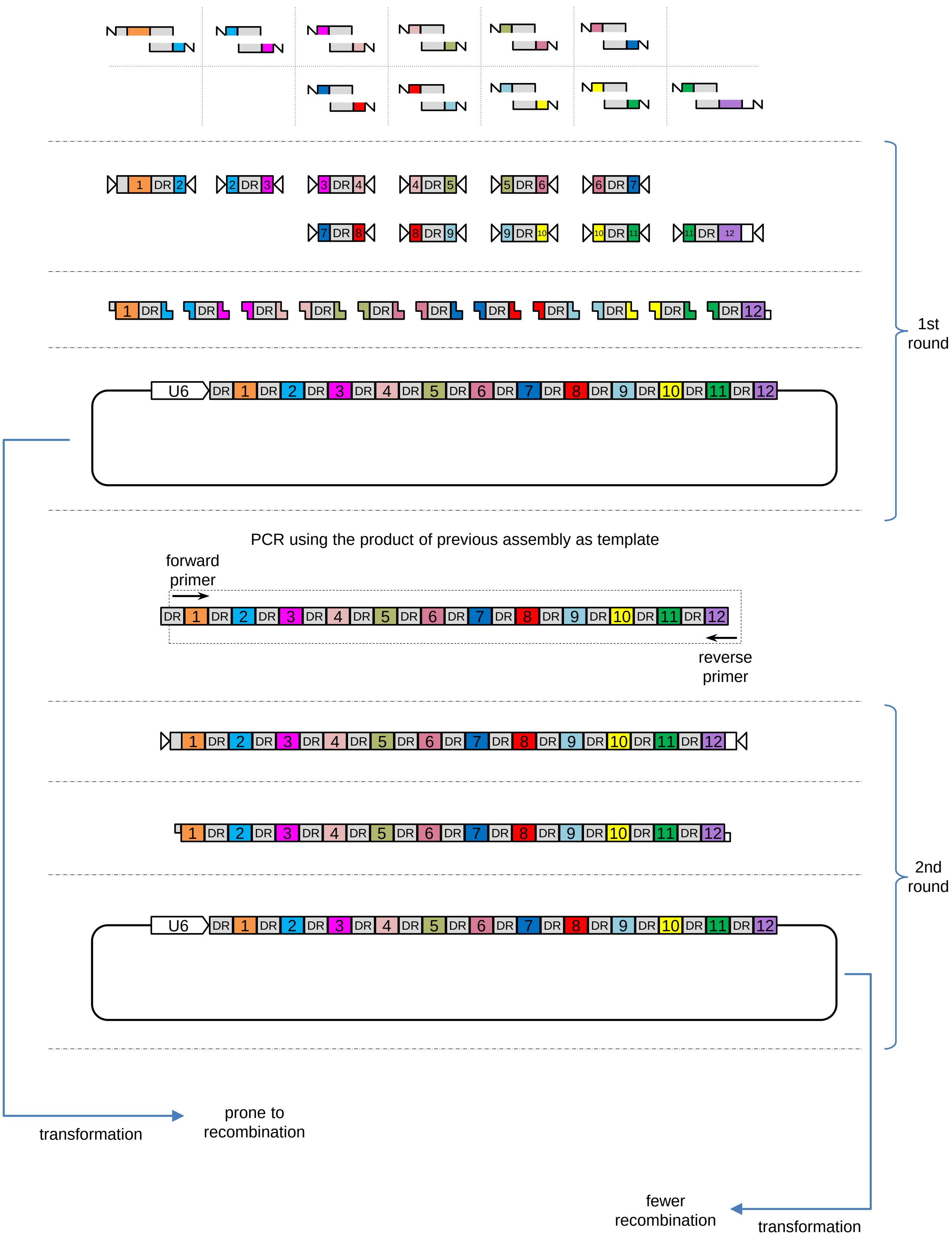

Figure S16. Schematic illustration and workflow of assembling CRISPR array using 2 rounds of assembly.

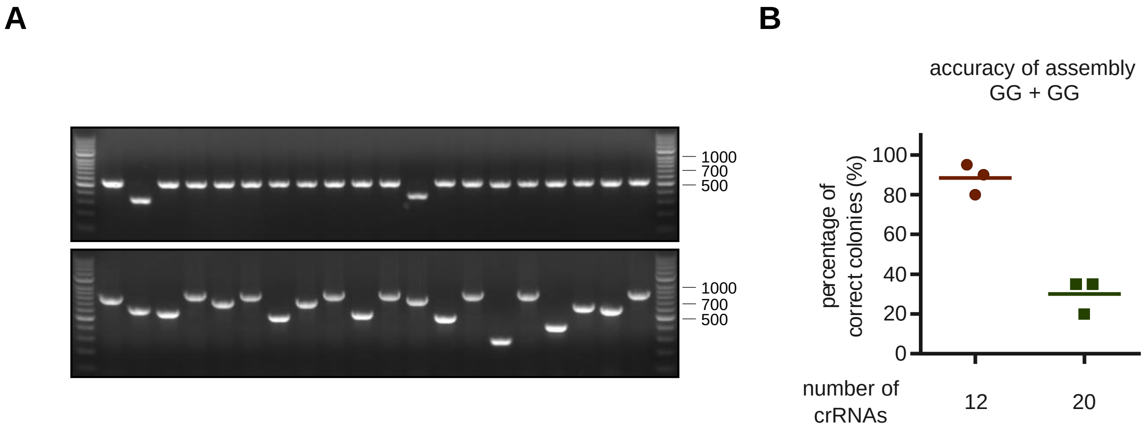

Figure S17. Accuracies of assembling 12 and 20 crRNAs using 2 rounds of assembly. (**A**) Representative images of colony PCR when assembling 12 (upper, correct size ≈ 530 nt) and 20 (lower, correct size ≈ 840 nt) crRNAs, and the corresponding accuracies are shown in (**B**). Note that since we failed to amplify the overall array from the assembly mixture of 20 crRNAs, the second round of PCR was separated into two individual reactions: crRNA 1-10, crRNA 11-20. Values shown as mean with n = 3.

A

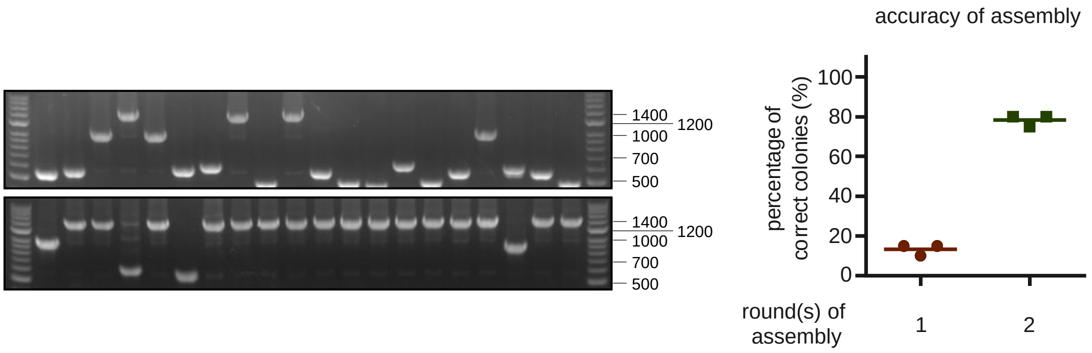

B

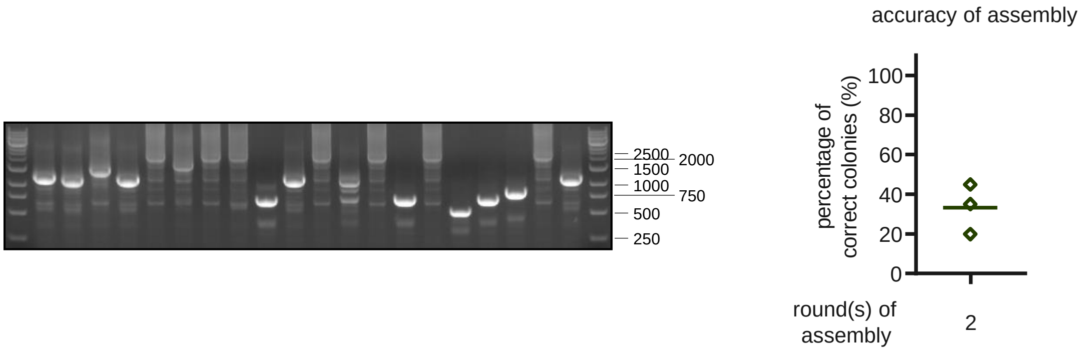

Figure S18. Assembly of complex, hierarchical CRISPR arrays. (**A**) Representative images of colony PCR when assembling an array consisting of 3 U6 promoters and 12 crRNAs (correct size ≈ 1450 nt) , by either a single (upper) or two (lower) rounds of assembly, and the corresponding accuracies are shown in (right). (**B**) Representative image of colony PCR when assembling an array consisting of 4 U6 promoters and 15 crRNAs by two rounds of assembly (correct size ≈ 2240 nt), and the corresponding accuracies are shown in (right). Values shown as mean with n = 3.

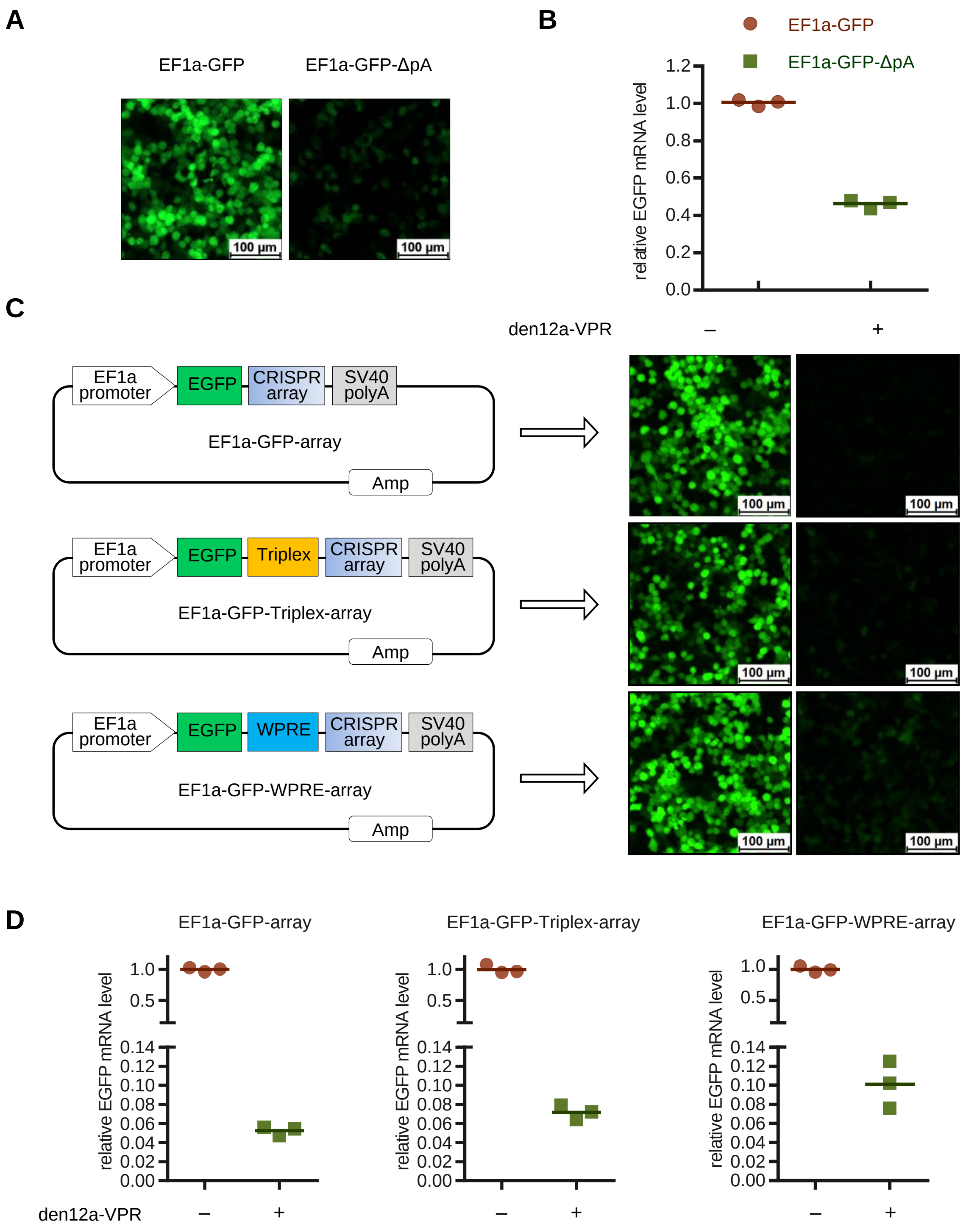

Figure S19. Processing of CRISPR array leads to inadequate expression of upstream EGFP encoded on the same transcript. **(A, B)** Representative fluorescence images **(A)** and mRNA expression **(B)** in HEK293T cells 48h after transfection with EF1a-driven EGFP constructs with/without a downstream SV40-poly(A) signal. Scale bar, 100 μm. **(C)** Schematics of EGFP constructs (left) and corresponding representative fluorescence images (right) 48h after transfection together with/without den12a-VPR. Scale bar, 100 μm. **(D)** Quantification of EGFP mRNA expression 48h after transfection with EGFP constructs together with/without den12a-VPR. Values shown as mean with n = 3.

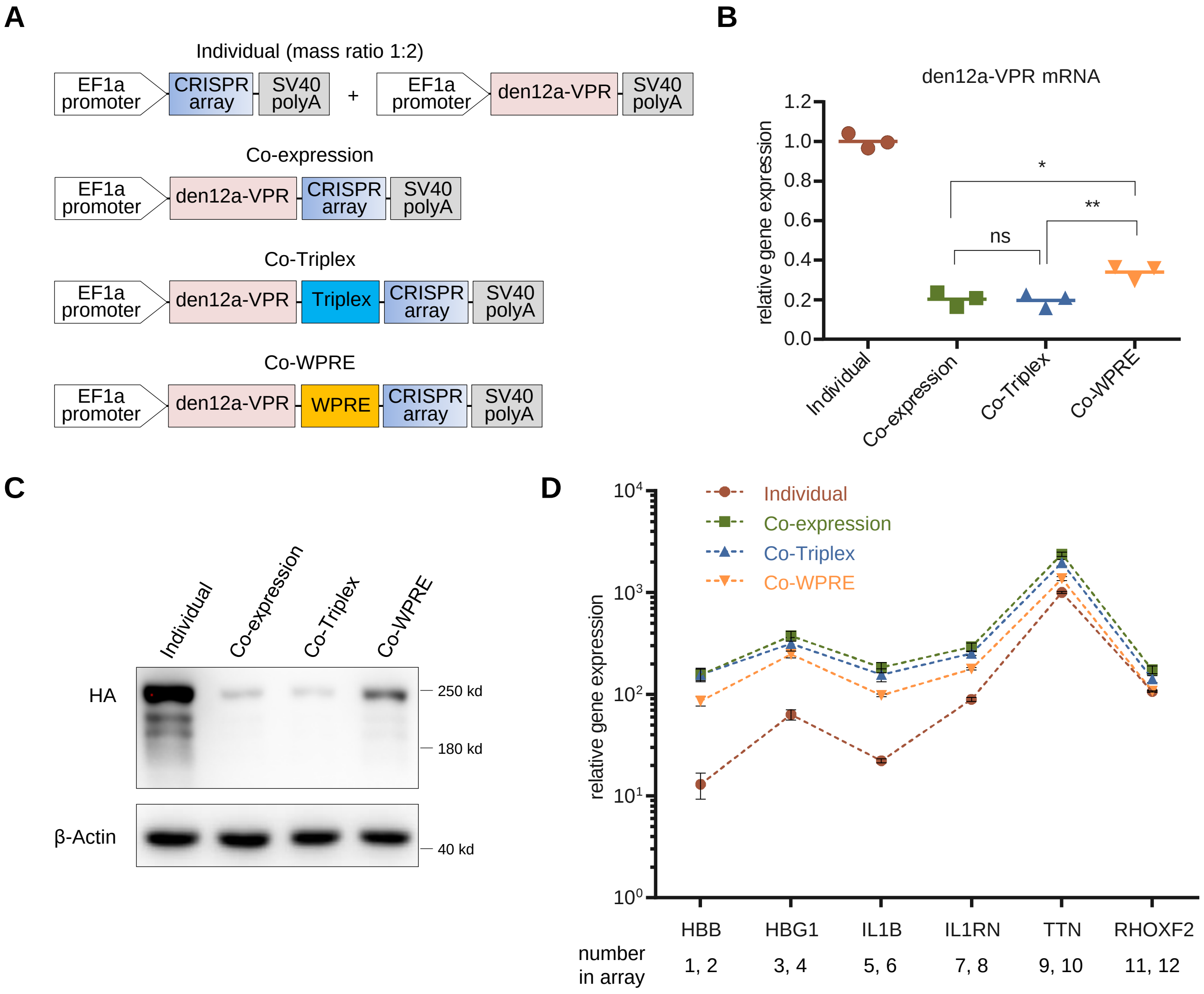

Figure S20. Co-expression of Cas protein and CRISPR array on a single transcript. (**A**) Schematics of constructs used to assess the targeting efficiency of co-expressing Cas protein and CRISPR array on a single transcript. (**B**, **C**) Relative mRNA (**B**) and protein (**C**) expression level of denAsCas12a-VPR in HEK293T cells 48h after transfection with constructs depicted in (**A**). Values shown as mean with n = 3. (**D**) Quantification of relative mRNA expression over non-targeting control in HEK293T cells 48h after transfection with constructs depicted in (**A**). Values shown as mean ± SD with n = 3.

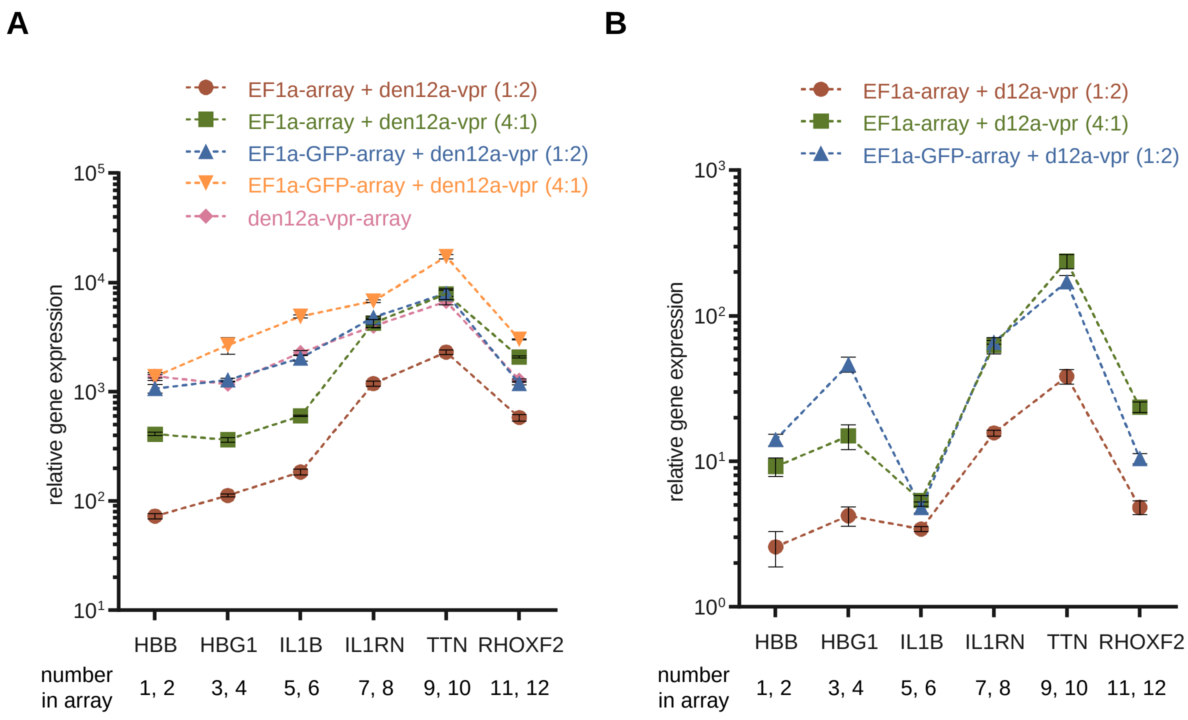

Figure S21. Comparison of the targeting efficiency across different mass ratios or expression patterns. **(A)** Quantification of relative mRNA expression over non-targeting control in HEK293T cells 48h after transfection with the indicated constructs at the mass ratios annotated in parentheses. **(B)** Quantification of relative mRNA expression over non-targeting control in HEK293T cells 48h after transfection with dAsCas12a-VPR and the indicated array-expressing constructs at the mass ratios annotated in parentheses. Values shown as mean ± SD with n = 3.

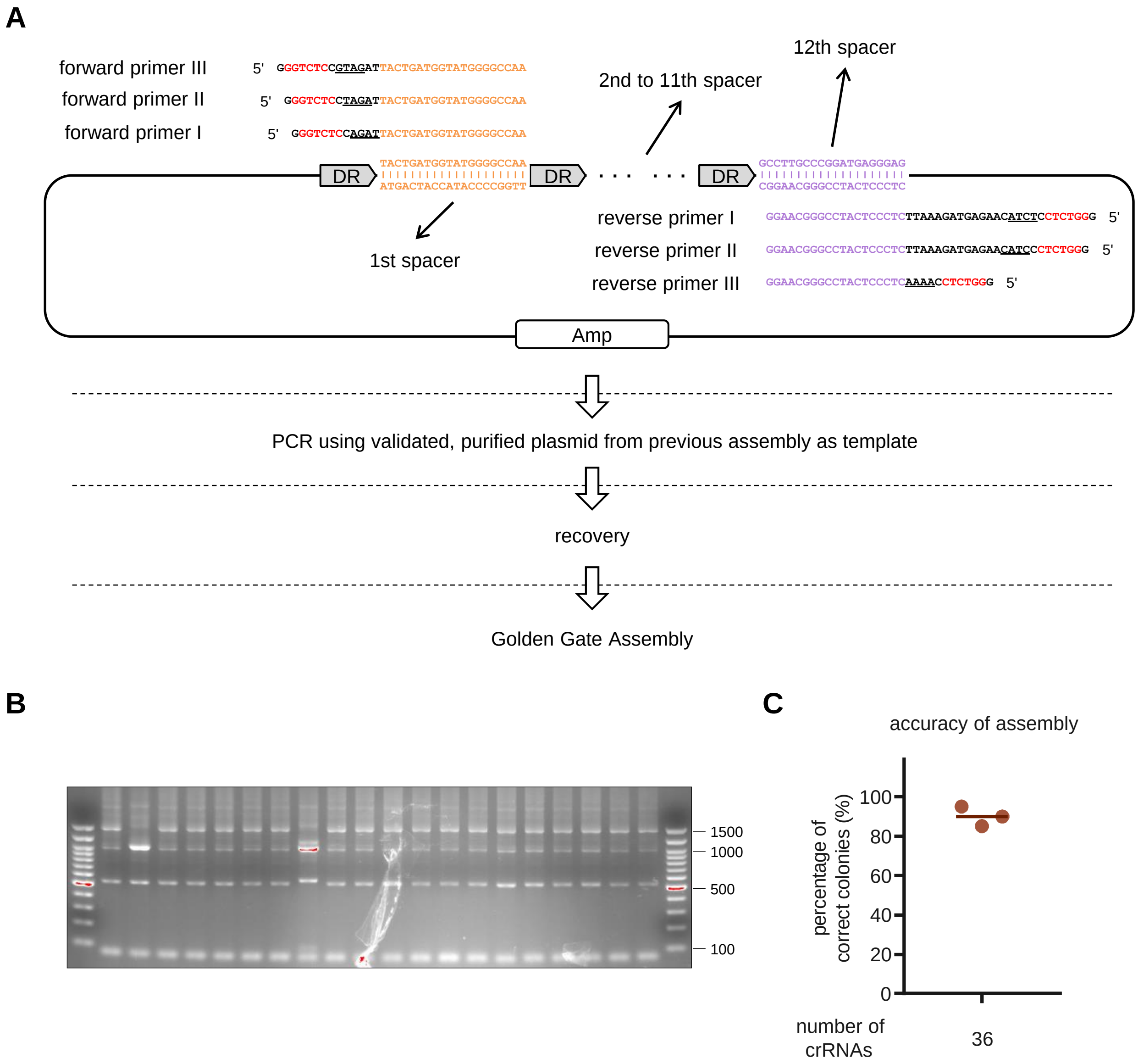

Figure S22. Generation of an array containing 36 crRNAs by two rounds of assembly. **(A)** Schematic illustration and workflow of the second round of assembly for the generation of an array containing 36 crRNAs. A detailed description of the first round of assembly is illustrated in Figure 1 and will not be shown here again. The resulting plasmid from the first round of assembly, encoding an array of 12 crRNAs, is used as a template for the second round of 3 PCR reactions (with primer pair I, II, III, respectively). Recovery the PCR products. Then, set up and run a standard Gold Gate assembly reaction with purified segments and destination cloning vector. **(B)** Representative images of colony PCR to evaluate the accuracy of the second round of assembly (correct size ≈ 1460 nt), and the corresponding accuracy are shown in **(C)**.

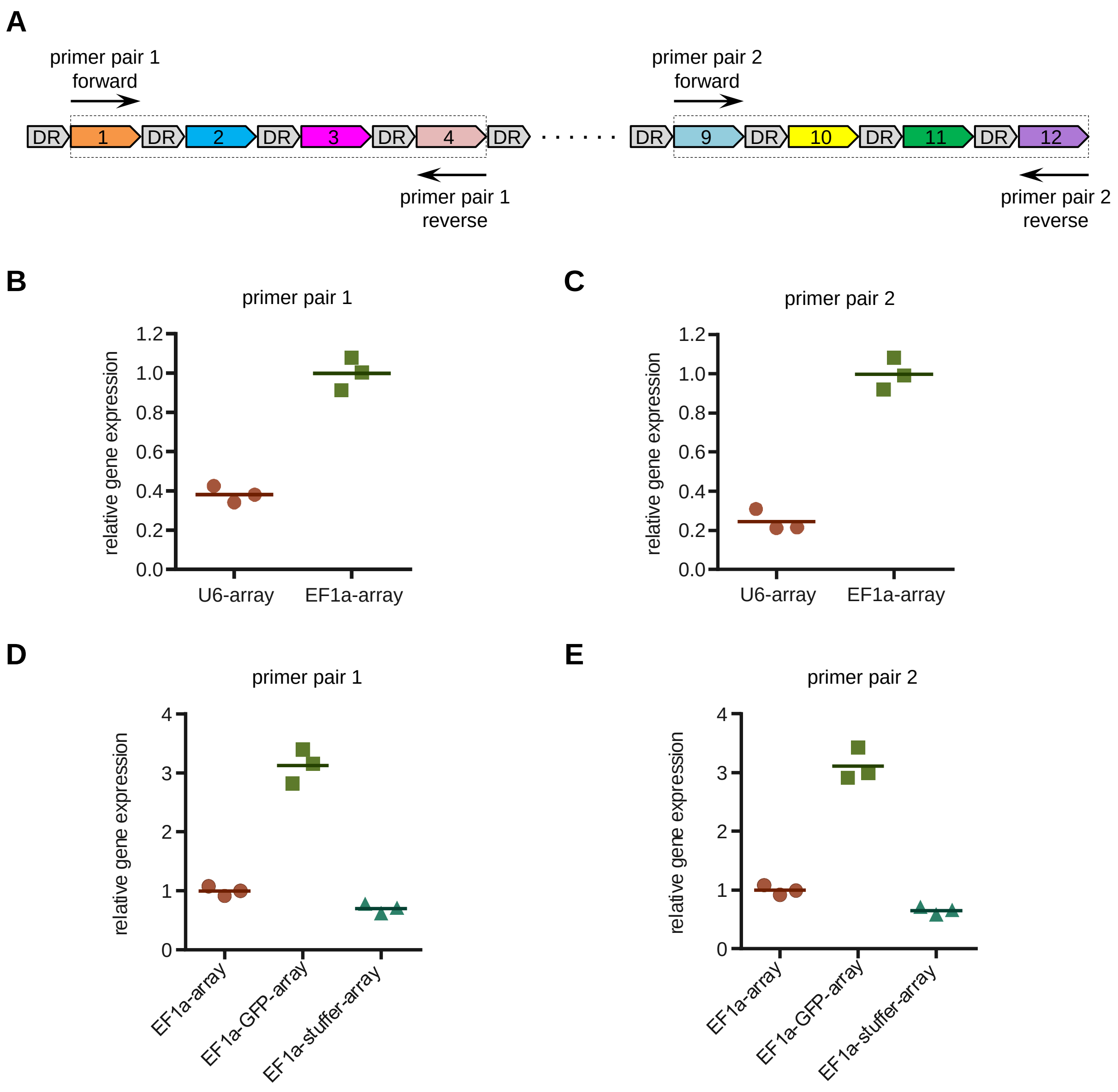

Figure S23. Expression of precursor arrays (for den12a-VPR) driven by Pol II or Pol III promoter. **(A)** Schematics of the two pairs of primers used to quantify the expression of CRISPR arrays. **(B**, **C)** Relative expression of the front (**B**) and back (**C**) parts of the CRISPR arrays driven by either U6 or EF1a. Values shown as mean with n = 3. **(D**, **E)** Relative expression of the front (**D**) and back (**E**) parts of the CRISPR arrays driven by EF1a with or without an upstream GFP or stuffer. Values shown as mean with n = 3.

An amount of 1 μg total RNA was reverse transcribed using ReverTra Ace® qPCR RT Kit (Toyobo) with respective reverse primer show in **Figure A**, followed by qPCR using ChamQ Universal SYBR qPCR Master Mix (Vazyme). qPCR reactions were performed using LightCycler 480 II (Roche). Quantification of RNA expression was normalized to ACTB (unless otherwise specified) and calculated using the ΔΔCt method.

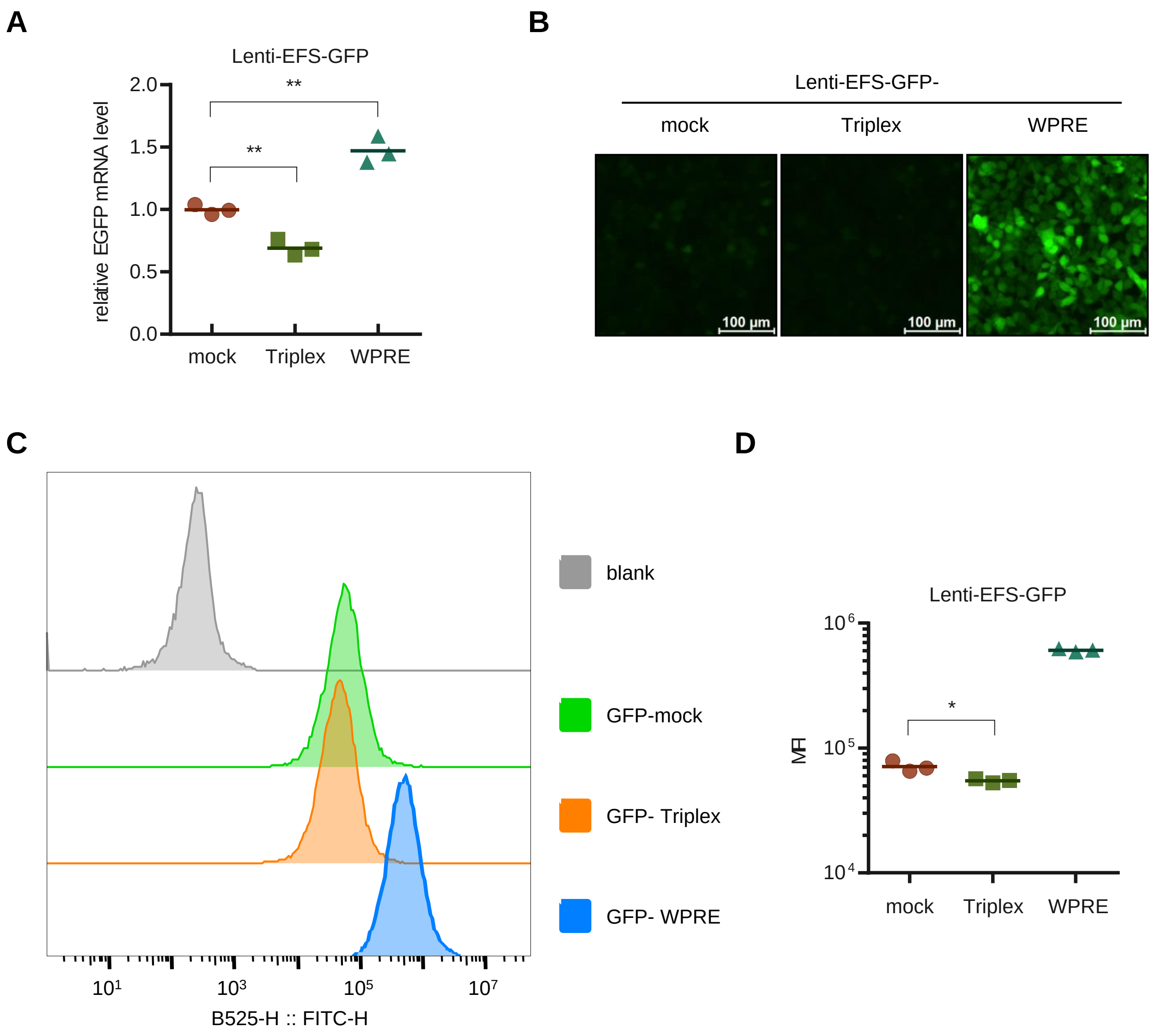

Figure S24. Introduction of WPRE, rather than the "Triplex" element, enhances the expression of gene delivered by lentivirus. **(A-D)** Quantification of EGFP mRNA expression **(A)**, representative fluorescence images **(B)**, representative fluorescence histograms detected by flow cytometry **(C)**, and mean fluorescence intensities **(D)** in HEK293T cells 72h after infection with EGFP-encoding lentivirus, either with or without WPRE or the "Triplex" element. Scale bar, 100 µm. Values shown as mean with n = 3.

Supplemental Methods

Protocol 1: Assembly of CRISPR arrays for (d)(en)AsCas12a with conventional sticky end-based strategies.

Reagents: BsaI-HF v2 (NEB, R3733), T4 DNA Ligase (NEB, M0202).

1) Set up the annealing reactions (total volume of 20 µl for each reaction) in PCR tubes as in **Table S23**.

2) Transfer PCR tubes to a thermocycler and run the annealing program as in **Table S24**.

3) Dilute annealed oligos to a final concentration of 0.5 µM (i.e., 5-fold dilution by adding 80 µl ddH_2O to each reaction).

4) Three different versions of assembly were performed individually as indicated in Figure S4: (-)BsaI, (+)BsaI, Gold Gate.

   4.1) Prepare the assembly reaction as in **Table S25**.

   Incubate at 16 ℃ overnight with a thermocycler.

   4.2) Prepare the assembly reaction as in **Table S26**.

   Incubate at 16 ℃ overnight with a thermocycler, followed by 37 ℃ for 15 min before transformation.

   4.3) Prepare the assembly reaction as in **Table S27**.

   Run the following Golden Gate Assembly program with a thermocycler: (37°C 5 min → 16 ℃ 5 min) × 30 cycles, followed by 60 ℃ for 5 min. If reactions are done overnight, add a 4 ℃ terminal hold to the program and repeat the step of 60 ℃ for 5 min the next day before transformation.

5) Transform 2-10 µl of the assembly reaction into competent cells.

Protocol 2: Assembly of CRISPR arrays for (d)(en)AsCas12a with GGA-based strategy.

Reagents: BsaI-HF v2 (NEB, R3733), T4 DNA Ligase (NEB, M0202), Taq DNA Polymerase (2 × premix), sodium acetate (3M, pH 5.2).

1) Set up the PCR reactions (total volume of 50 μl for each reaction) as in **Table S28**.

2) Transfer PCR tubes to a thermocycler and run a routine PCR program as in **Table S29**.

3) Upon completion, transfer PCR products to 1.5 ml tubes. Add 1/10 volume of NaAc (3M, pH 5.2), and 2 volumes of pre-chilled 100% ethanol. Mix well. Store at -20 ℃ for 20 min to overnight to precipitate the DNA.

4) Centrifuge at 10,000-20,000g for 15 min (at 4 ℃ if convenient). Carefully pour off the ethanol (or remove by pipetting) without disturbing the pellet. Wash twice with 70-75% ethanol. Allow the DNA pellet to air-dry. Dissolve the DNA 20 μl TE buffer or ddH_2O.

5) Quantify the concentration of recovered DNA samples. Adjust them to a uniform concentration (e.g., around 5 ng/ μl).

6) Prepare the assembly reaction as in **Table S30**.

7) Run the following Golden Gate Assembly program with a thermocycler: (37°C 5 min → 16°C 5 min) × 30-60 cycles, followed by 60 ℃ for 5 min. If reactions are done overnight, add a 4 ℃ terminal hold to the program and repeat the step of 60 ℃ for 5 min the next day before transformation.

8) Transform 2-10 μl of the assembly reaction into competent cells.

Protocol 3: A simplified version of Protocol 2.

Reagents: BsaI-HF v2 (NEB, R3733), T4 DNA Ligase (NEB, M0202), Taq DNA Polymerase (2 × premix).

Note: The optimal primer concentration for PCR reaction and the volume pipetted for subsequent assembly were determined based on the designated PCR reagent used in this study. Although these reaction parameters may need to be optimized for different PCR reagents, they can be used as benchmarks. When necessary, adjust them up or down to obtain satisfactory results.

1) Set up the PCR reactions (total volume of 20 μl for each reaction) as in **Table S31**.

2) Transfer PCR tubes to a thermocycler and run a routine PCR program as in **Table S32**.

3) Upon completion, dilute PCR products 10-fold by adding 180 μl ddH_2O to each reaction.

4) Prepare the assembly reaction as in **Table S33**.

5) Run the following Golden Gate Assembly program with a thermocycler: (37°C 5 min → 16°C 5 min) × 30-60 cycles, followed by 60 ℃ for 5 min. If reactions are done overnight, add a 4 ℃ terminal hold to the program and repeat the step of 60 ℃ for 5 min the next day before transformation.

6) Transform 2-10 μl of the assembly reaction into competent cells.

Protocol 4: Assembly of CRISPR arrays for RfxCas13d with GGA-based strategy.

Reagents: BsaI-HF v2 (NEB, R3733), T4 DNA Ligase (NEB, M0202), PrimeSTAR HS DNA Polymerase (Takara, R010A).

1) Set up the PCR reactions (total volume of 50 μl for each reaction) as in **Table S34**.
2) Transfer PCR tubes to a thermocycler and run a routine PCR program as in **Table S35**.
3) Upon completion, purify PCR segments with available DNA recovery kit. Quantify the concentration. Adjust them to a uniform concentration (e.g., around 5 ng/μl).
4) Prepare the assembly reaction as in **Table S36**.
5) Run the following Golden Gate Assembly program with a thermocycler: (37°C 5 min → 16°C 5 min) × 30-60 cycles, followed by 60 ℃ for 5 min. If reactions are done overnight, add a 4 ℃ terminal hold to the program and repeat the step of 60 ℃ for 5 min the next day before transformation.
6) Transform 2-10 μl of the assembly reaction into competent cells.

Supplemental Tables

Table S1. Primers used for qPCR.

Table S2. Gene-specific primers used for reverse transcription (also used as the reverse primer of subsequent qPCR) and counterpart qPCR primers.

Table S3. Spacer sequences for transcriptional activation mediated by dAsCas12a-VPR or denAsCas12a-VPR.

Table S4. Spacer sequences for RfxCas13d-mediated RNA cleavage.

Table S5. Spacer sequence for gene editing mediated by enAsCas12a.

Table S6. Primers used for the amplification and sequencing of the target region flanking indels.

Table S7. Primers used to assemble the CRISPR array of 6 crRNAs for dAsCas12a-VPR or denAsCas12a-VPR with conventional sticky end-based strategies.

Table S8. Primers used to assemble the CRISPR array of 7 crRNAs for dAsCas12a-VPR or denAsCas12a-VPR with conventional sticky end-based strategies.

Table S9. Primers used to assemble the CRISPR array of 6 crRNAs for dAsCas12a-VPR or denAsCas12a-VPR with GGA-based strategy.

Table S10. Primers used to assemble the CRISPR array of 7 crRNAs for dAsCas12a-VPR or denAsCas12a-VPR with GGA-based strategy.

Table S11. Primers used to assemble the CRISPR array of 9 crRNAs for dAsCas12a-VPR or denAsCas12a-VPR with GGA-based strategy.

Table S12. Primers used to assemble the CRISPR array of 12 crRNAs for dAsCas12a-VPR or denAsCas12a-VPR with GGA-based strategy.

Table S13. Primers used to assemble 12 crRNAs for dAsCas12a-VPR or denAsCas12a-VPR based on mutant DR.

Table S14. Primers used to assemble the CRISPR array of 9 crRNAs for RfxCas13d with GGA-based strategy.

Table S15. Primers used to assemble the CRISPR array of 12 crRNAs for RfxCas13d with GGA-based strategy.

Table S16. Primers used to assemble the CRISPR array of 15 crRNAs for RfxCas13d with GGA-based strategy.

Table S17. Primers used to assemble 12 crRNAs for dAsCas12a-VPR or denAsCas12a-VPR with two rounds of assembly.

Table S18. Primers used to assemble 20 crRNAs for dAsCas12a-VPR or denAsCas12a-VPR with two rounds of assembly.

Table S19. Primers used to assemble U6-4-U6-4-U6-4 for dAsCas12a-VPR or denAsCas12a-VPR with two rounds of assembly.

Table S20. Primers used to assemble U6-4-U6-4-U6-4-U6-3 for RfxCas13d with two rounds of assembly.

Table S21. Sequences of 5' bioton labelled DNA probes used for Northern blots of mature crRNAs.

Table S22. Sequences of constructs used in this study.

Table S23. Corresponding to step 1) of **Protocol 1**.

component	volume	final concentration
forward primer (10 μM)	5 μl	2.5 μM
reverse primer (10 μM)	5 μl	2.5 μM
T4 DNA Ligase Buffer (10×)	2 μl	1×
ddH_2O	8 μl	

Table S24. Corresponding to step 2) of **Protocol 1**.

step	temperature	duration	cycles
1	95℃	4 min	
2	70℃	10 min	
3	70℃ (set: -0.1℃/cycle)	2 sec	
4	go to step 3		500
end			

Table S25. Corresponding to step 4.1) of **Protocol 1**.

component	volume/amount
diluted annealed oligos (0.5 μM)	1 μl each
recovered predigested destination vector	30-80 ng
T4 DNA Ligase Buffer (10×)	2 μl
T4 DNA Ligase	0.5 μl
ddH_2O	to 20 μl

Table S26. Corresponding to step 4.2) of **Protocol 1**.

component	volume/amount
diluted annealed oligos (0.5 μM)	1 μl each
recovered predigested destination vector	30-80 ng
T4 DNA Ligase Buffer (10×)	2 μl
BsaI-HF v2	1 μl
T4 DNA Ligase	0.5 μl
ddH_2O	to 20 μl

Table S27. Corresponding to step 4.3) of **Protocol 1**.

component	volume/amount
diluted annealed oligos (0.5 μM)	1 μl each
destination vector	30-80 ng
T4 DNA Ligase Buffer (10×)	2 μl
BsaI-HF v2	1 μl
T4 DNA Ligase	0.5 μl
ddH_2O	to 20 μl

Table S28. Corresponding to step 1) of **Protocol 2**.

component	volume	final concentration
forward primer (10 μM)	5 μl	1 μM
reverse primer (10 μM)	5 μl	1 μM
Taq premix (2×)	25 μl	1×
ddH_2O	15 μl	

Table S29. Corresponding to step 2) of **Protocol 2**.

step	temperature	duration	cycles
1	95℃	3 min	
2	95℃	30 sec	
3	45-50℃	30 sec	
4	72℃	30 sec	
5	go to step 2		34
6	72℃	5 min	

Table S30. Corresponding to step 6) of **Protocol 2**.

component	volume/amount
recovered PCR segments	~5 ng each
destination vector	30-80 ng
T4 DNA Ligase Buffer (10×)	2 μl
BsaI-HF v2	1-2 μl
T4 DNA Ligase	0.5 μl
ddH_2O	to 20 μl

Table S31. Corresponding to step 1) of **Protocol 3**.

component	volume	final concentration
forward primer (10 μM)	5 μl	2.5 μM
reverse primer (10 μM)	5 μl	2.5 μM
Taq premix (2×)	10 μl	1×

Table S32. Corresponding to step 2) of **Protocol 3**.

step	temperature	duration	cycles
1	95℃	3 min	
2	95℃	30 sec	
3	45-50℃	30 sec	
4	72℃	30 sec	
5	go to step 2		34
6	72℃	5 min	

Table S33. Corresponding to step 4) of **Protocol 3**.

component	volume/amount
diluted PCR products	1 μl each
destination vector	30-80 ng
T4 DNA Ligase Buffer (10×)	2 μl
BsaI-HF v2	1-2 μl
T4 DNA Ligase	0.5 μl
ddH_2O	to 20 μl

Table S34. Corresponding to step 1) of **Protocol 4**.

component	volume/amount	final concentration
5 ×Buffer (Mg^{2+} Plus)	10 μl	1 ×
dNTP mixture (2.5 mM each)	4 μl	200 μM each
DNA polymerase	0.5 μl	
forward primer (10 μM)	5 μl	1 μM
reverse primer (10 μM)	5 μl	1 μM
template plasmid	0.2-1 ng	
ddH_2O	to 50 μl	

Table S35. Corresponding to step 2) of **Protocol 4**.

step	Temperature	duration	cycles
1	98℃	30 sec	
2	98℃	10 sec	
3	50℃	15 sec	
4	72℃	30 sec	
5	go to step 2		34
6	72℃	5 min	

Table S36. Corresponding to step 4) of **Protocol 4**.

component	volume/amount
recovered PCR segments	~5 ng each
destination vector	30-80 ng
T4 DNA Ligase Buffer (10×)	2 μl
BsaI-HF v2	1-2 μl
T4 DNA Ligase	0.5 μl
ddH_2O	to 20 μl

Table S37. Sanger sequencing results in FASTA format of correctly sized colonies.

Table S38. Sanger sequencing results in FASTA format of incorrectly sized colonies.
