# Supplementary material for: High-accuracy crRNA array assembly strategy for multiplex CRISPR
Source: Mol Ther Nucleic Acids. 2024 Dec 12;36(1):102428. doi: 10.1016/j.omtn.2024.102428 (PMC11787013; doi:10.1016/j.omtn.2024.102428)
Supplement: Document S2. Article plus supplemental information [file mmc3.pdf]

Molecular Therapy
Nucleic Acids
Original Article

High-accuracy crRNA array assembly strategy for multiplex CRISPR

Xiangtong Zhao,[1,2,6] Lixian Yang,[3,6] Peng Li,[4] Zijing Cheng,[5] Yongshi Jia,[3] Limin Luo,[3] Aihong Bi,[3] Hanchu Xiong,[3] Haibo Zhang,[3] Hongen Xu,[3] Jinrui Zhang,[1] and Yaodong Zhang[1]

[1]Henan Provincial Key Laboratory of Children's Genetics and Metabolic Diseases, Children's Hospital Affiliated to Zhengzhou University, Henan Children's Hospital, Zhengzhou Children's Hospital, Zhengzhou, Henan, China; [2]College of Public Health, Zhengzhou University, Zhengzhou, Henan, China; [3]Cancer Center, Department of Radiation Oncology, Zhejiang Provincial People's Hospital, Affiliated People's Hospital, Hangzhou Medical College, Hangzhou, Zhejiang, China; [4]Department of Gastroenterology, Zhejiang Provincial People's Hospital, Affiliated People's Hospital, Hangzhou Medical College, Hangzhou, Zhejiang, China; [5]School of Anesthesiology, Xuzhou Medical University, Xuzhou, Jiangsu, China

Simultaneous targeting of multiple loci with the CRISPR system, a tool known as multiplex CRISPR, offers greater feasibility for manipulating and elucidating the intricate and redundant endogenous networks underlying complex cellular functions. Owing to the versatility of continuously emerging Cas nucleases and the use of CRISPR arrays, multiplex CRISPR has been implemented in numerous *in vitro* and *in vivo* studies. However, a streamlined, practical strategy for CRISPR array assembly that is both convenient and accurate is lacking. Here, we present a novel, highly accurate, cost-, and time-saving strategy for CRISPR array assembly. Using this strategy, we efficiently assembled 12 CRISPR RNAs (crRNAs) (for AsCas12a) and 15 crRNAs (for RfxCas13d) in a single reaction. CRISPR arrays driven by Pol II promoters exhibited a distinct expression pattern compared with those driven by Pol III promoters, which could be exploited for specific distributions of CRISPR intensity. Improved approaches were subsequently designed and validated for expressing long CRISPR arrays. The study provides a flexible and powerful tool for the convenient implementation of multiplex CRISPR across DNA and RNA, facilitating the dissection of sophisticated cellular networks and the future realization of multi-target gene therapy.

INTRODUCTION

Over the past decade, following the pioneering discovery and eukaryotic implementation of the CRISPR-Cas9 system,[1–3] a growing number of new CRISPR systems have been developed.[4] As a substrate of the best-known Cas9, DNA is no longer the only target within our reach. With the discovery of various RNA-targeting Cas nucleases,[5–11] RNA can now be precisely targeted, further extending our range of genetic manipulation from the genome to the transcriptome. Moreover, by combining transcriptional activators,[12] repressors,[13] base editors,[14] epigenetic enzymes,[15] reverse transcriptases,[16] and tagging molecules,[17] these RNA-guided effectors have magnified the molecular toolbox of basic research. Most important, the CRISPR systems have demonstrated unlimited potential for the treatment of a growing number of incurable genetic diseases.[18–23]

As indicated by the definition of CRISPR, the natural CRISPR RNA (crRNA) in bacteria and archaea is usually not transcribed individually but in a clustered preform from a genomic locus called the CRISPR array, which consists of a succession of direct repeats (DRs) separated by distinct spacers.[4] Subsequently, the transcribed pre-crRNA is processed into a series of mature crRNAs, each containing a single spacer and a truncated DR. Unlike Cas9, which requires an additional transactivating crRNA to form a final guide RNA with the crRNA (usually replaced in practice by a chimeric single guide RNA), many other CRISPR systems use mature crRNAs directly as guide RNAs. Moreover, the Cas nucleases in these systems typically possess the ability to process pre-crRNAs.[4] These two features render these Cas effectors more amenable to multiplex CRISPR, because a single array is sufficient to express all the required crRNAs.

Current strategies for assembling customized CRISPR arrays are based on the conventional method of cloning a single crRNA, except that the number of annealed oligos is multiplied. In fact, such theoretically feasible strategies may work when assembling only a few crRNAs, but are quite incompetent when assembling more crRNAs, as demonstrated here. Therefore, an accurate, efficient, and practical strategy for the CRISPR array assembly is required. In the present study, we designed a novel cost-effective strategy for CRISPR array

Received 5 December 2023; accepted 10 December 2024;
https://doi.org/10.1016/j.omtn.2024.102428.

[6]These authors contributed equally

Correspondence: Xiangtong Zhao, Henan Provincial Key Laboratory of Children's Genetics and Metabolic Diseases, Children's Hospital Affiliated to Zhengzhou University, Henan Children's Hospital, Zhengzhou Children's Hospital, Zhengzhou, Henan, China.
E-mail: zhaoxiangtong@zuaa.zju.edu.cn
Correspondence: Lixian Yang, Cancer Center, Department of Radiation Oncology, Zhejiang Provincial People's Hospital, Affiliated People's Hospital, Hangzhou Medical College, Hangzhou, Zhejiang, China.
E-mail: yanglixian@hmc.edu.cn
Correspondence: Yaodong Zhang, Henan Provincial Key Laboratory of Children's Genetics and Metabolic Diseases, Children's Hospital Affiliated to Zhengzhou University, Henan Children's Hospital, Zhengzhou Children's Hospital, Zhengzhou, Henan, China.
E-mail: syek@163.com

Published by Elsevier Inc. on behalf of The American Society of Gene and Cell Therapy.
This is an open access article under the CC BY license (http://creativecommons.org/licenses/by/4.0/).

assembly and confirmed its superior accuracy over current alternatives using the AsCas12a system. While preserving its accuracy as much as possible, several simplifications and optimizations were made to make this strategy more user friendly. Next, this strategy was used to assemble crRNAs for an RNA-targeting CRISPR system, highlighting its general applicability, flexibility, and high accuracy. When attempting to harness Pol II promoters for CRISPR array expression, a distinct expression pattern of CRISPR arrays transcribed by EF1a (Pol II promoter) was found, compared with the more commonly used U6 (Pol III promoter). For long CRISPR arrays (>200 nt), we designed a better approach based on U6 by using its edges and circumventing its drawbacks. These tandem, hierarchical arrays achieved improved targeting efficiency in CRISPR systems, which require the abundant expression of crRNAs. Finally, by reexamining the strategy of co-expressing Cas nuclease and the CRISPR array on a single transcript, an unavoidable detrimental effect on the expression of Cas nuclease was discovered, despite its unexpectedly satisfactory targeting performance with some CRISPR systems in certain applications. Further investigation of this anomaly indicated that the introduction of an upstream GFP-coding sequence could enhance the expression of Pol II promoter-driven CRISPR arrays. This finding provides an alternative approach for efficient expression of long CRISPR arrays.

RESULTS

Design of a novel strategy for CRISPR array assembly

Given that it is more convenient and cost efficient when probing targeting efficiency, a catalytically dead AsCas12a fused to an artificial VP64-p65-Rta transcription activator (dAsCas12a-VPR, abbreviated as d12a-VPR hereafter) was used for subsequent experiments. An enhanced variant (denAsCas12a-VPR, abbreviated as den12a-VPR hereafter) was also used.[24] Similar to Cas9-based CRISPR activators,[25–28] the combination of multiple crRNAs enabled the synergistic activation of endogenous gene targets mediated by the d12a-VPR and den12a-VPR (Figure S1). Next, we assessed the accuracy of different strategies for the assembly of crRNAs for AsCas12a. Current strategies for the assembly of multiple crRNAs typically consist of two main procedures (Figure S2): annealing of predesigned, single-stranded DNA oligos to form double-stranded DNA (dsDNA) with the desired sticky ends, followed by sequential ligation into cloning vectors. Because of their similar principles, we classified them as sticky end-based strategies. The accuracy of these assemblies relies on the precise sequential ligation of the successfully annealed oligos. In practice, however, not all oligos eventually end up annealed to their respective partners as desired, i.e., a fair number of oligos remain single-stranded after annealing. These single-stranded oligos can be a source of trouble, as their natively exposed ends are identical to the sticky ends of the annealed dsDNA, meaning that any site of sequential ligation can be occupied by them, leading to irreversible premature termination of the assembly. This intrinsic defect is likely the dominant factor limiting the number of crRNAs that can be assembled into an array in a single reaction using sticky end-based strategies. The preliminary experiments suggested that only up to six crRNAs could be efficiently assembled in a single assembly (data not shown).

To overcome this limitation, we designed a novel strategy for CRISPR array assembly that aimed to increase both accuracy and maximal number by eliminating the perturbations of single-strand oligos (Figure 1). In brief, several additional bases containing a BsaI recognition site were appended to the 5′ end of each single-strand oligo, so that the designated inner sticky-end was not exposed until a dsDNA was formed and cut by BsaI (Figure S3). These short dsDNA segments can be generated mainly by two alternative methods: (1) annealing of complete complementary oligo pairs, which are particularly long because of the need for BsaI recognition sites at both ends of the resulting dsDNA, and (2) a PCR-based approach using much shorter oligo pairs that are partially complementary to each other or to a certain template. We chose the latter because it is cost effective and an annealing-free strategy could be tested that would be substantially different from conventional sticky end-based methods.

No additional procedures follow the PCR reaction, only optional routine recovery of DNA segments, and subsequent standard Golden Gate Assembly (GGA). To this end, we named this novel approach a GGA-based strategy.

High-accuracy assembly of CRISPR array with GGA-based strategy

There are several versions of detailed conventional sticky end-based protocols that differ slightly from each other (with or without predigestion before ligation or redigestion after ligation). Their accuracy was compared by assembling six crRNAs. Whereas no correct clones were obtained when the ligation mixture was directly transformed into *Escherichia coli*, additional redigestion with BsaI between ligation and transformation dramatically increased the accuracy of the selected clones (Figure S4). Compared with the remaining two procedures, the assembly using standard Golden Gate cycles exhibited improvement or non-inferiority without the procedure of predigesting or recovering the cloning vector (Figure S4); hence, this specified sticky end-based strategy was used for the following evaluation and comparison.

Next, using our GGA-based strategy, an array containing six crRNAs was assembled with parallel controls using a sticky end-based strategy for comparison. Whereas all 60 clones were correct when using the GGA-based strategy, assembly using the sticky end-based strategy achieved a mean accuracy of only 37% ($p < 0.0001$, GGA versus sticky end) (Figures 2A and S5A). When the number of crRNAs was increased to seven, the accuracy of the sticky end-based assembly strategy decreased sharply, with only 1 correct out of 60 clones. Although a marked reduction was observed, the GGA-based assembly strategy retained a mean accuracy of 73% (Figures 2B and S5B).

Having demonstrated the superiority of our GGA-based strategy over conventional sticky end-based strategies, the number of crRNAs to be assembled was increased until the accuracy decreased to a level substantially lower than 50%. Following this criterion, and considering that the extremely low accuracy in assembling seven crRNAs was already practically meaningless, the sticky end-based strategy was

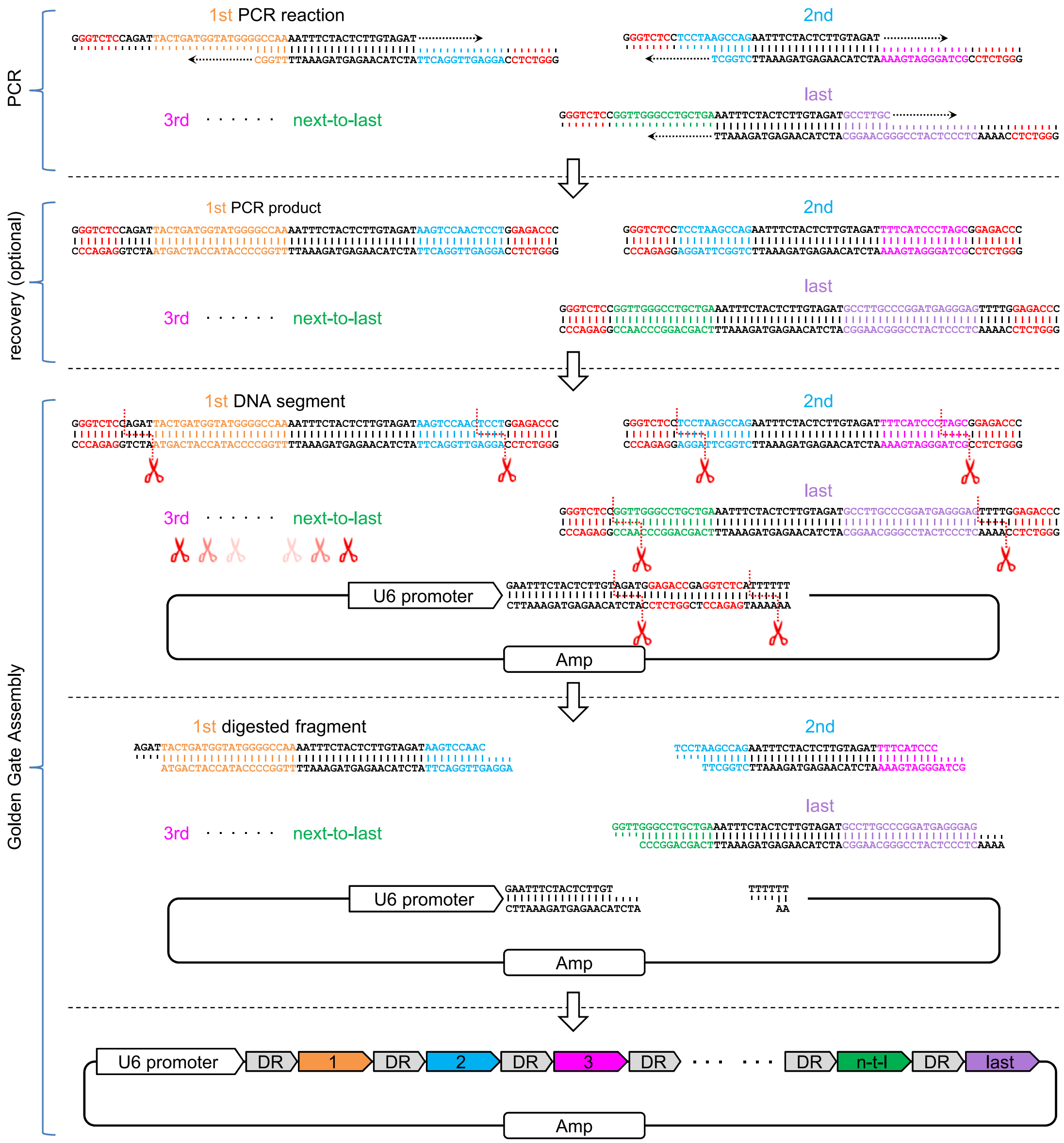

Figure 1. Schematic workflow of GGA-based CRISPR array assembly strategy

First, PCRs were set up and PCRs were run with predesigned oligo pairs that were partially complementary to each other. Next, an optional recovery step was performed if necessary (because of the relatively short DR length of AsCas12a, some of the resulting dsDNA segments were too short to be recovered using common DNA recovery kits; therefore, we used a relatively crude method via ethanol precipitation. Although single-stranded oligos or dNTPs might still be included, most of the salts were removed, and the DNA polymerase was inactivated). Finally, a standard GGA reaction was set up and run with the purified segments (or diluted PCR products) and a destination cloning vector.

no longer evaluated for assembling more crRNAs. Using the GGA-based strategy, arrays of 9 or 12 crRNAs were efficiently assembled, with mean accuracies of 67% and 43%, respectively (Figures 2C and S5C). For the assembly of the 12 crRNAs, colonies from an additional replicate experiment were sequenced to confirm our results and investigate the source of errors in incorrect inserts during the revision of this manuscript (plasmid 37) (Table S22). We found that 14 of the 15 full-length inserts obtained the desired sequences with no errors (Figure S6). A variety of unusual constructs were seen for inserts of small size, with one particular error arising from the apparent misassembly of the fusion site from fragment GGGTCTCCGACTGCCCACAAGTGCTAATTCCTACTCTTGTAGGTAATGAATGT**GTGC**GGAGACCwith fragment GGGTCTCC**TAGC**CAGCCAATTCCTACTCTTGTAGGTAAGTCCAGGAGACC. It is unclear whether these errors represent mistakes generated during GGA owing to *in vivo* repair events or a mixture of the two.

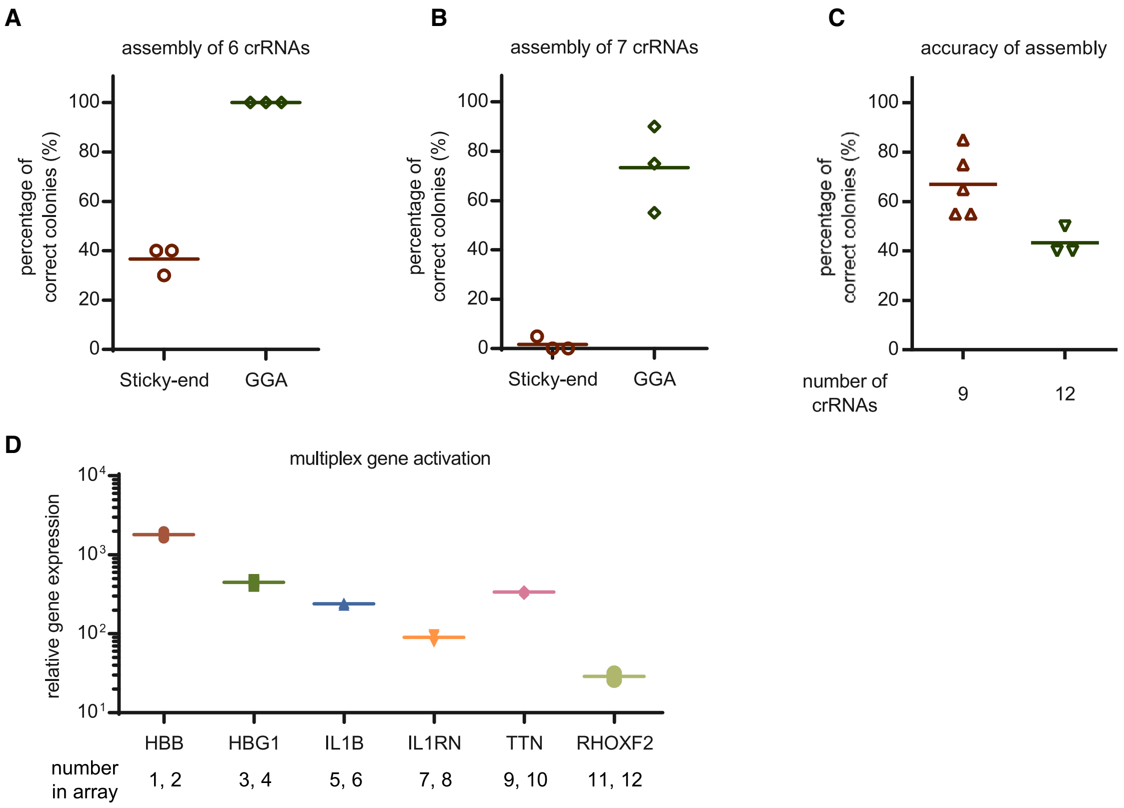

Figure 2. High-accuracy assembly of CRISPR array with GGA-based strategy

(A and B) Accuracies of the assembly of six (A) or seven (B) crRNAs using the conventional sticky end-based or novel GGA-based strategy. Values shown as mean, $n = 3$ independent experiments. (C) Accuracies of the assembly of 9 or 12 crRNAs using the GGA-based strategy. Values shown as mean with $n \geq 3$. (D) Quantification of relative mRNA expression over the non-targeting control in HEK293T cells 48 h after transfection with a single plasmid containing CBh-driven denAsCas12a-VPR and a U6-driven CRISPR array of 12 crRNAs targeting the indicated 6 genes. CBh, a robust Pol II promoter. Values shown as mean ± SD with $n = 3$.

Although arrays containing more than 12 crRNAs may still be assembled in a single reaction, in view of the gradual decrease in assembly accuracy as the number of crRNAs increased, the practical value of such experiments would be limited; thus, we ceased here and determined the maximum number of crRNAs for AsCas12a that could be assembled into an array in a single reaction to be 12.

The resulting array of 12 crRNAs contained spacers targeting the promoters of six endogenous human genes (two crRNAs for each gene): *HBB*, *HBG1*, *IL1B*, *IL1RN*, *TTN*, and *RHOXF2*. Together with this array, the den12a-VPR achieved robust activation of these six genes simultaneously in HEK293T cells from 20- to 2,000-fold (Figure 2D).

Simplification and optimization of the GGA-based strategy for CRISPR array assembly

Although our GGA-based assembly strategy exhibited greater accuracy than the alternatives, the requirement for an additional recovery procedure for PCR products may hinder its wider application. To address this, we sought to circumvent this procedure while preserving assembly accuracy as much as possible. Instead of the time-consuming recovery required to obtain purified dsDNA segments in the standard assembly protocol, the PCR products were diluted and added directly to the GGA reaction. From a wide range of PCR primer concentrations and volumes of PCR products pipetted for subsequent assembly, the best accuracy was achieved when using a final primer concentration of 2.5 μM for PCR and 0.1 μL (i.e., 1 μL after 10-fold dilution) from each of the resulting PCR mixtures for a 20-μL assembly reaction (for the designated DNA polymerase; data not shown). Assemblies of 6, 7, 9, and 12 crRNAs were performed following this reaction condition. The mixing of unwanted PCR components into the subsequent assembly reaction compromised the final accuracy, but only to a modest degree, with the exception of the assembly of 12 crRNAs, from which the correct clones were barely obtained (Figure S7). In addition, assembly failures could also be attributed to erroneous amplification products (e.g., primer dimers), which could be actively assembled because they also contain BsaI sites that produce compatible overhangs. Nevertheless, this simplified recovery-free version of our GGA-based strategy outperformed current alternative assembly strategies without the requirement for additional procedures and could be a rational choice when assembling no more than nine crRNAs.

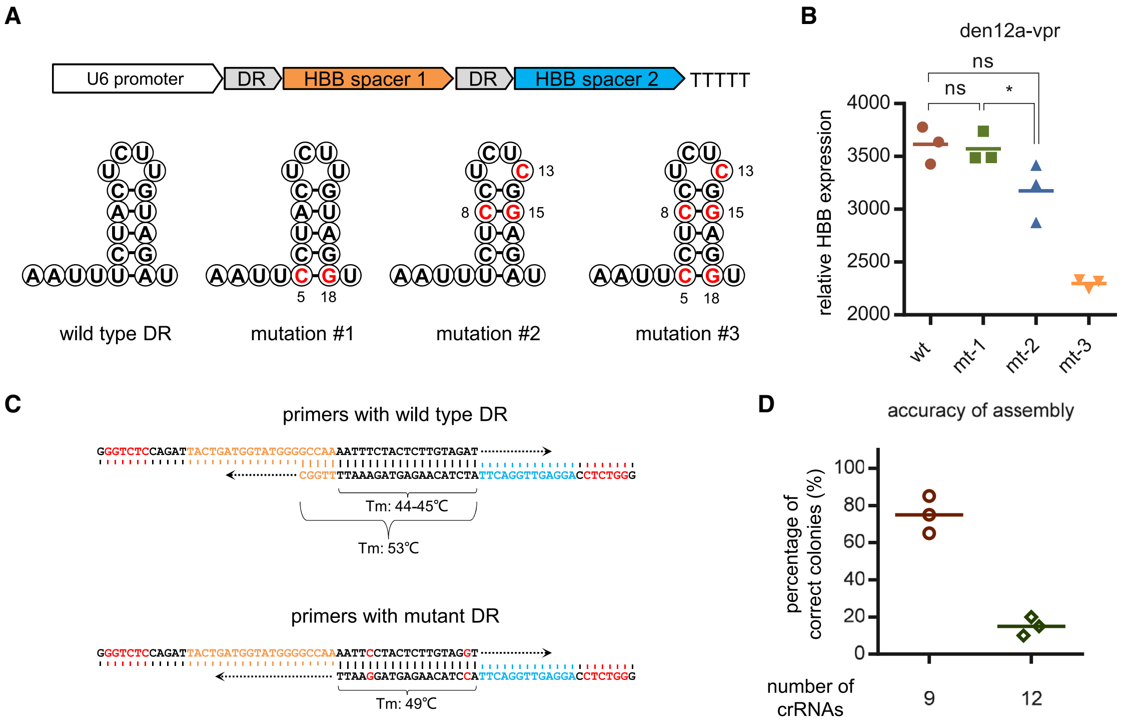

Figure 3. Simplification of the GGA-based strategy for CRISPR array assembly

(A) Schematics of wild-type and three mutant DR variants of the AsCas12a system. (B) Quantification of relative *HBB* expression over the non-targeting control in HEK293T cells 48 h after transfection with den12a-VPR and arrays with DR variants depicted in (A). (C) An example showcasing the streamlining of the initial primer design by using a mutant DR with higher GC content. (D) Accuracies in the assembly of 9 or 12 crRNAs with mutant DR using the GGA-based strategy. Values shown as mean with n = 3.

Another shortcoming that may complicate the assembly strategy is the DR sequence of AsCas12a. The 19 nt DR of AsCas12a has a relatively low melting temperature (44°C–45°C, T_m) due to its low GC content (approximately 26%). This intrinsic property makes it challenging to design initial primers for subsequent PCR reactions, since oligos must be extended beyond the DR sequence to obtain a minimal appropriate melt temperature (T_m 50°C–55°C). To address this issue, we sought to introduce mutations into the original wild-type DR, with the aim of increasing its T_m value to a proper extent (>50°C) without compromising its function. Several previous studies have made such attempts but with distinct aims.[29–31] Based on their findings, we generated three variants of wild-type DR (Figure 3A). Of these candidates, one variant with a T_m of 49°C showed non-inferiority in targeting efficiency compared with the wild-type DR (Figure 3B). Although the T_m of this variant was still slightly below 50°C, it was already feasible to use the DR sequence directly as the end of primers and 45°C as the annealing temperature for subsequent PCR reactions simplified the primer design procedure (Figure 3C). Based on this DR variant, arrays of nine and 12 crRNAs were successfully assembled, with mean accuracies of 75% and 15%, respectively (Figures 3D and S8). Moreover, the assembly of an array containing nine crRNAs was accomplished efficiently when both mutant DR and the recovery-free version of the GGA-based strategy were used (Figure S9).

High-accuracy assembly of CRISPR array for a Cas13d nuclease

Whereas the previous content focused on AsCas12a, the potential applications of the assembly strategy can be extended beyond this specific CRISPR system. Owing to its flexibility and generalizability, this crRNA array assembly strategy can theoretically applied to most CRISPR systems in which crRNA contains a DR followed by a spacer, especially when the Cas nuclease has the ability to process its own crRNA.

To confirm this concept, we used this crRNA array assembly strategy to assemble crRNAs from another distinct CRISPR system, Cas13d, a family of RNA-targeting Cas nucleases.[4] Of these, RfxCas13d was chosen for subsequent assessment.[9] The commonly used crRNA for RfxCas13d is composed of a 36 nt DR and a spacer of 22–30 nt. An intermediate spacer length of 26 nt was selected for the following CRISPR array assembly. In contrast with that of AsCas12a, the DR of RfxCas13d was much longer and had a modest GC content (50%). These properties render the assembly of its crRNAs more suitable for our strategy: (1) design of the primers was much easier than that of AsCas12a, as all the forward and reverse primers could use the same approximately 20 base 3′ section; and (2) using current alternatives for such a CRISPR system with long DR sequence would be more costly, since it would be unavoidable to purchase particularly long oligos (>60 bases), which are usually several times more expensive per

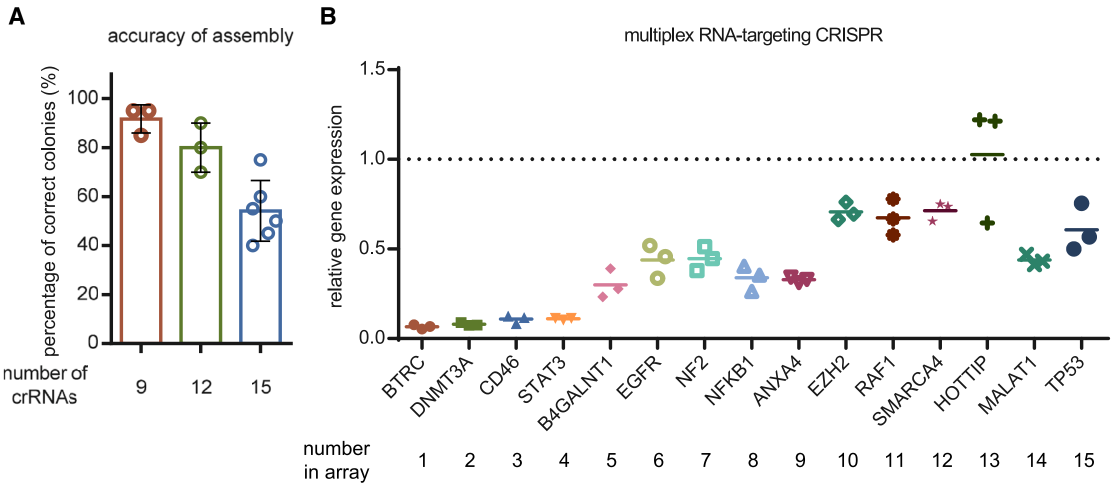

Figure 4. High-accuracy assembly of crRNAs for a Cas13d nuclease

(A) Accuracies of the assembly of 9, 12, or 15 crRNAs for RfxCas13d using the GGA-based strategy. Values shown as mean with $n \geq 3$. (B) Quantification of relative mRNA expression compared with the non-targeting control in HEK293T cells 48 h after transfection with a plasmid containing EF1a-RfxCas13d and a U6-driven CRISPR array of 15 crRNAs targeting the indicated 15 endogenous transcripts. Values shown as mean with $n = 3$.

base than the shorter routine oligos due to their complex production process. The detailed procedure was the same as that for the assembly of crRNAs for AsCas12a, with two minor modifications (Figure S10). The resulting procedure was reminiscent of several previous strategies for assembling sgRNA cassettes used in Cas9-based multiplex CRISPR.[32–34]

The GGA-based assembly strategy performed better with RfxCas13d than with AsCas12a, as an array of up to 15 crRNAs could be assembled in one pot with acceptable accuracy (Figures 4A and S11). This improved performance may be due to purer dsDNA segments or other uncertain reasons. Using this array, the simultaneous cleavage of 15 endogenous transcripts was attempted. Forty-eight hours after transfection, most of these transcripts were efficiently cleaved and detected using the corresponding primers flanking the cleavage sites (Figure 4B).

Distinct expression patterns of Pol II/III promoter-driven CRISPR arrays

Although the array of 15 crRNAs used for RfxCas13d reached a length of approximately 1,200 nt, the U6 promoter still seemed capable of driving its transcription. This disagreed with the conventional conception that Pol III promoters (e.g., U6, H1, and 7SK) are typically used for the transcription of small RNAs, although RNA up to 800 nucleotides have also been reported to be efficiently transcribed by U6.[35] One solution to this indeterminate restriction was to replace Pol III promoters with Pol II promoters (e.g., cytomegalovirus [CMV] and EF1a), as they are capable of transcribing much longer RNAs. Several studies have evaluated the ability of harnessing Pol II promoters to express crRNAs or synthetic sgRNAs.[36,37]

To determine whether CRISPR arrays transcribed by Pol II promoters work as efficiently as those transcribed by Pol III promoters, HEK293T cells were transfected with den12a-VPR and an array of 12 crRNAs driven by either U6 (U6-array) or EF1a (EF1a-array), followed by quantification of the expression of the corresponding six targets 48 h after transfection. No straightforward determination of superiority or inferiority could be drawn from the results, because each had its own strengths and weaknesses (Figure 5A). Northern blots of mature crRNAs showed that U6 outperformed EF1a in transcribing the first four crRNAs, particularly the first two, but was inferior for all subsequent crRNAs (Figure 5B). When targeting individual genes using two crRNAs, U6 outperformed EF1a for both *HBB* and *RHOXF2* (Figures 5C and 5D), suggesting that U6 was superior to EF1a when driving the expression of short CRISPR arrays. Because we could not rule out the possibility that the crRNAs expressed from U6 cells were already saturated, the disparity observed here might have been underestimated. Indeed, a larger gap was observed by varying the ratio of den12a-VPR to the array (Figure S12). However, no disparity between these promoters was observed during gene editing using nuclease-active AsCas12a (Figure 5E). The reason might be that the number of targets in the genome was limited, and once edited with an indel, CRISPR elements were no longer needed, meaning that a small amount of crRNA was already sufficient, and more would be redundant.

To determine whether this phenomenon represented a general pattern, we compared it with other CRISPR systems. Forty-eight hours after transfection with RfxCas13d and an array of 15 crRNAs driven by either U6 or EF1a, no conclusions could be drawn except that the U6-array showed superiority in targeting the first four transcripts (Figure S13A). When harnessed for the expression of a single

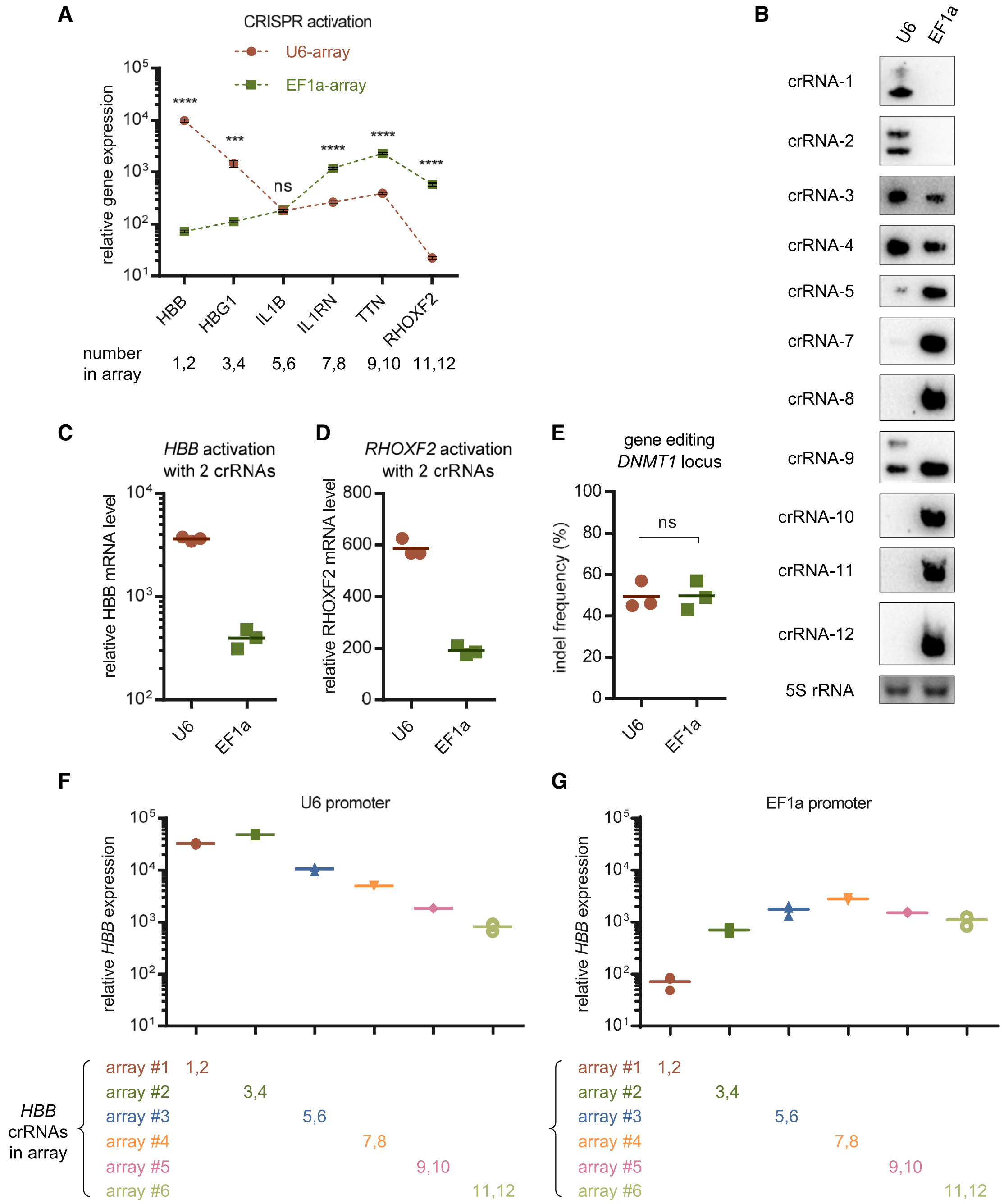

Figure 5. Distinct expression patterns of Pol II/III promoter-driven CRISPR arrays

(A) Quantification of relative mRNA expression over the non-targeting control in HEK293T cells 48 h after transfection with EF1a-denAsCas12a-VPR and an array of 12 crRNAs driven by either U6 (U6-array) or EF1a (EF1a-array). Values shown as mean ± SD with $n = 3$. (B) Representative northern blot images of mature crRNAs processed from CRISPR arrays driven by U6 or EF1a. Blots of crRNA-6 were not detected because the corresponding probe was not working well. (C and D) Quantification of the indicated gene expression over the non-targeting control in HEK293T cells 48 h after transfection with EF1a-denAsCas12a-VPR and a U6- or EF1a-driven array of 2 crRNAs targeting the promoter of *HBB* (C) or *RHOXF2* (D). (E) Quantification of gene editing efficiency in HEK293T cells 72 h after transfection with enAsCas12a and a *DNMT1*-targeting crRNA driven by either U6 or EF1a. (F and G) Quantification of relative *HBB* expression over the non-targeting control in HEK293T cells 48 h after transfection with EF1a-denAsCas12a-VPR and the indicated arrays (12×) driven by U6 (F) or EF1a (G), where HBB-targeting crRNAs were positioned at different loci. Values shown as mean with $n = 3$.

crRNA, EF1a exhibited a substantially lower target cleavage efficiency than U6 (Figure S13B).

In contrast with the EF1a-array, the location of the crRNAs in the U6-array seemed to have a greater influence on their own expression. When the internal order of the CRISPR array was reversed, the U6-array experienced a more drastic change in multiplex CRISPR efficiency (Figure S14). To obtain an intuitive and precise correlation between transcriptional strength and distance from the promoter, the two crRNAs targeting the HBB promoter were placed at a series of different loci while the overall component was kept constant (Figures 5F and 5G). The first few crRNAs of the U6-array achieved very high levels of expression, which could not be reached by EF1a. Thereafter, the intensity of transcription decreased continuously with increasing distance from U6. In contrast, the distance from the EF1a promoter had a much weaker effect on crRNA expression, except that the first few crRNAs might undergo inadequate expression.

These results suggest that Pol II and III promoters are not functionally equivalent when driving the expression of CRISPR arrays. While U6 favored the expression and execution of the first few crRNAs within approximately 200 nt, EF1a seemed to distribute its relatively milder strength more evenly. For single crRNA or small CRISPR arrays (<200 nt), especially when using the RfxCas13d system, harnessing Pol II promoters may still be an inadequate alternative unless mild expression was acceptable.

Improve the targeting efficiency by manipulating the architecture of long CRISPR arrays

Having determined the superiority of U6 over EF1a when transcribing the first few crRNAs within approximately 200 nt, we decided to find a better approach for the transcription of longer CRISPR arrays than simply selecting one from either U6 or EF1a. By splitting long CRISPR arrays into several short arrays, each of them could be assigned a U6 promoter, thus using the edge of U6 in the strength of transcribing short RNAs while circumventing its disadvantage in transcribing long RNAs. The principle and workflow were similar to those previously described in the current study, with the only difference being that small PCR segments containing crRNAs and longer PCR segments containing U6 were mixed and assembled in one reaction (Figure S15). However, we failed to assemble the correct array that matched our expectations for our maiden attempt (data not shown). This may be attributed to the introduction of repeated U6 elements, which make the resulting array more prone to recombination events.

In previous studies, we amplified an array of 12 or 15 crRNAs as an intact sequence from a purified plasmid that already contained the entire array (considered the second round of PCR) and then cloned this unique insert into a new vector (considered the second round of assembly), which is a simple procedure to transfer an existing array from one vector to another. Although the accuracy of assembling 12 or 15 individual crRNAs into one array in the first round was as low as 15% (Figure 3D), the second round always achieved an accuracy of greater than 80% (data not shown). Although we were unable to provide an explicit interpretation of this phenomenon, we exploited it by performing a second round of assembly to reboot the unsuccessful assembly of the hierarchical CRISPR array. For the sake of time, the assembly mixture of the first round was directly used as the PCR template for the second round instead of the validated and purified plasmid (Figure S16). The mean accuracy of the array assembly with 12 crRNAs improved to 88% (Figure S17). However, amplifying the entire array from the crude mixture of previous assembly may not always succeed, especially when the destination fragment spans more than a dozen crRNAs. An array of 20 crRNAs was successfully assembled in two rounds; however, we performed two individual amplifications with two pairs of primers in the second round (Figure S17).

To improve accuracy by performing an additional round of assembly, we decided to confront the obstacle of assembling hierarchical CRISPR arrays again. In the second round, an RfxCas13d CRISPR array consisting of four U6-crRNAs units was successfully assembled with mean accuracy of 33% (Figure S18B), which had been shown to be impossible in the first round. Similarly, this strategy substantially increased the accuracy of the denAsCas12a CRISPR array assembly consisting of three U6-crRNAs units (Figure S18A).

In contrast with arrays driven by a single U6 or EF1a, the tandem array driven by three U6 promoters achieved robust transcriptional activation of all six targets in the denAsCas12a-VPR mediated multiplex CRISPR system (Figures 6A and 6B). Similarly, for most of the 15 endogenous transcripts, RfxCas13d showed more prominent cleavage efficiency when working with a tandem array driven by four U6 (Figures 6C and 6D).

Enhance the expression of Pol II promoter-driven CRISPR arrays by introducing an upstream GFP-coding sequence

Since Pol II promoters are capable of driving the expression of CRISPR arrays in addition to protein-encoding genes, one might consider the possibility of co-expressing the Cas protein and CRISPR array on a single transcript. To achieve this type of co-expression, the coding sequence of the Cas protein must be positioned upstream of the CRISPR array, followed by a poly(A) tail. However, this arrangement poses a problem: processing of the CRISPR array inevitably leads to the separation of the Cas protein mRNA from the poly(A) tail, ultimately resulting in inadequate expression of the Cas protein and unsatisfactory CRISPR efficiency. A previous study attempted to address this issue by harnessing a putative mRNA-stabilizing element called Triplex.[37] Their results showed that the introduction of Triplex between EGFP and the CRISPR array completely rescued the loss of EGFP fluorescence due to the processing of downstream array. We are concerned about this kind of complete rescue, as it challenges the irreplaceable role of poly(A) tails in stabilizing eukaryotic mRNA.[38]

To test this hypothesis, we investigated whether this specific co-expression pattern is a viable option for multiplex CRISPR.

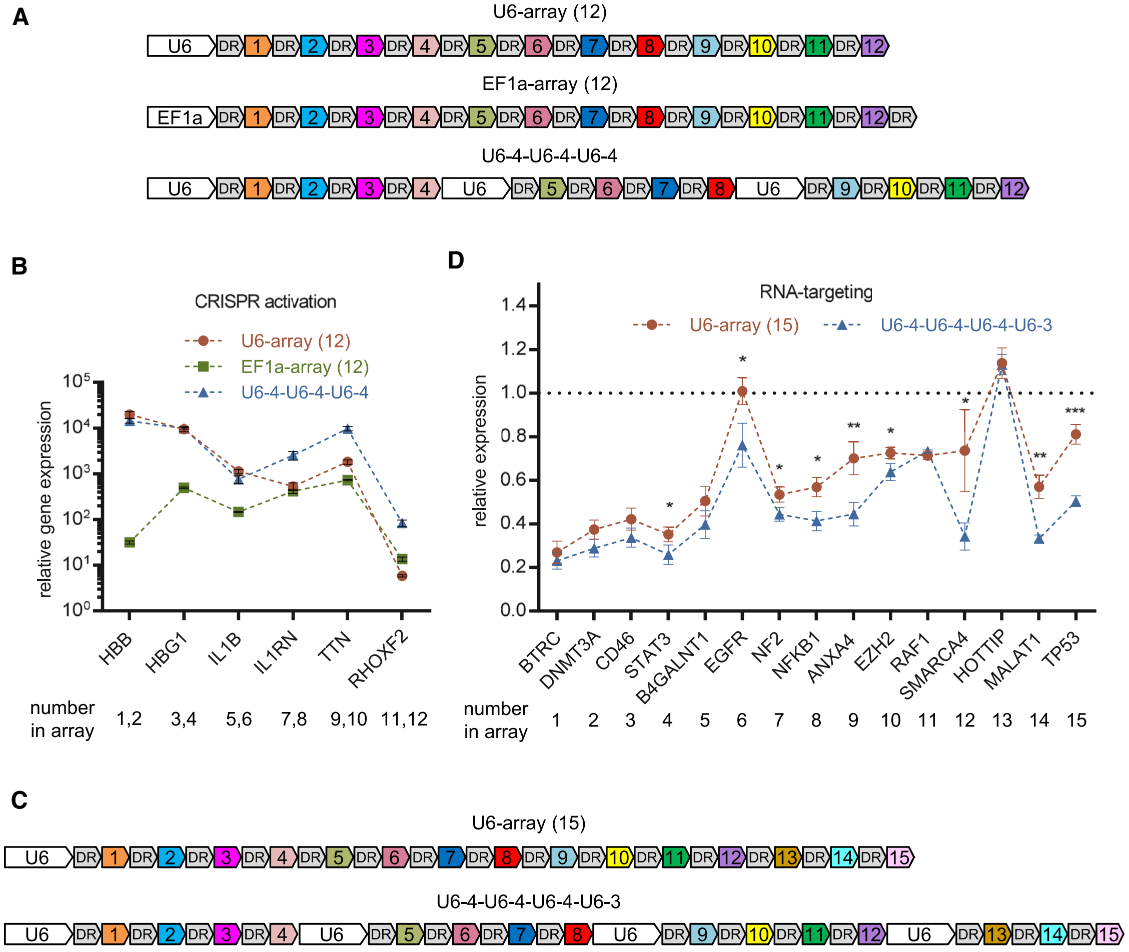

Figure 6. Improved targeting efficiency by optimizing the internal architecture of the CRISPR array

(A) Schematics of CRISPR arrays used with denAsCas12a-VPR: U6-array (12), EF1a-array (12), and U6-4-U6-4-U6-4. (B) Quantification of relative mRNA expression over the non-targeting control in HEK293T cells 48 h after transfection with EF1a-denAsCas12a-VPR and the indicated arrays. Values shown as mean ± SD with $n = 3$. (C) Schematics of CRISPR arrays used with RfxCas13d: U6-array (15) and U6-4-U6-4-U6-4-U6-3. (D) Quantification of relative mRNA expression of the indicated genes compared with the non-targeting control in HEK293T cells 48 h after transfection with RfxCas13d and the indicated arrays. Values shown as mean ± SD with $n = 3$.

Consistent with the conventional understanding, removal of the poly(A) tail remarkably reduced EGFP expression at both the mRNA and protein levels (Figures S19A and S19B), emphasizing the crucial role of the poly(A) tail in stabilizing mRNA. To examine the mRNA-stabilizing efficacy of Triplex and the well-known woodchuck hepatitis virus post-transcriptional regulatory element (WPRE), we cloned EGFP downstream of the EF1a promoter, followed by an array of 12 crRNAs for AsCas12a, with or without a Triplex or WPRE element, between EGFP and the CRISPR array. The resulting plasmids were transfected into HEK293T cells with the control vector or den12a-VPR. Forty-eight hours after transfection, as a result of the processing of crRNAs, the simultaneous expression of the den12a-VPR dramatically reduced EGFP mRNA and fluorescence. Distinct from a previous study,[37] the additional introduction of Triplex or WPRE resulted in mild, if any, improvement in the mRNA or fluorescence of EGFP (Figures S19C and S19D).

The targeting efficiency of co-expressing the den12a-VPR and CRISPR arrays on a single transcript was directly evaluated next (Figure S20A). Co-expression via direct integration resulted in a marked reduction in den12a-VPR mRNA/protein expression but not in the ultimate CRISPRa efficiency, which increased (Figures S20B–S20D). This discrepancy between decreased den12a-VPR expression and enhanced CRISPRa efficiency might be a result of increased expression of the CRISPR array due to the insertion of the upstream den12a-VPR. When the EF1a-array and den12a-VPR were delivered by individual constructs, the expression of the CRISPR array and the final CRISPRa efficiency were enhanced by inserting an EGFP-coding sequence upstream of the CRISPR array, but not by inserting a

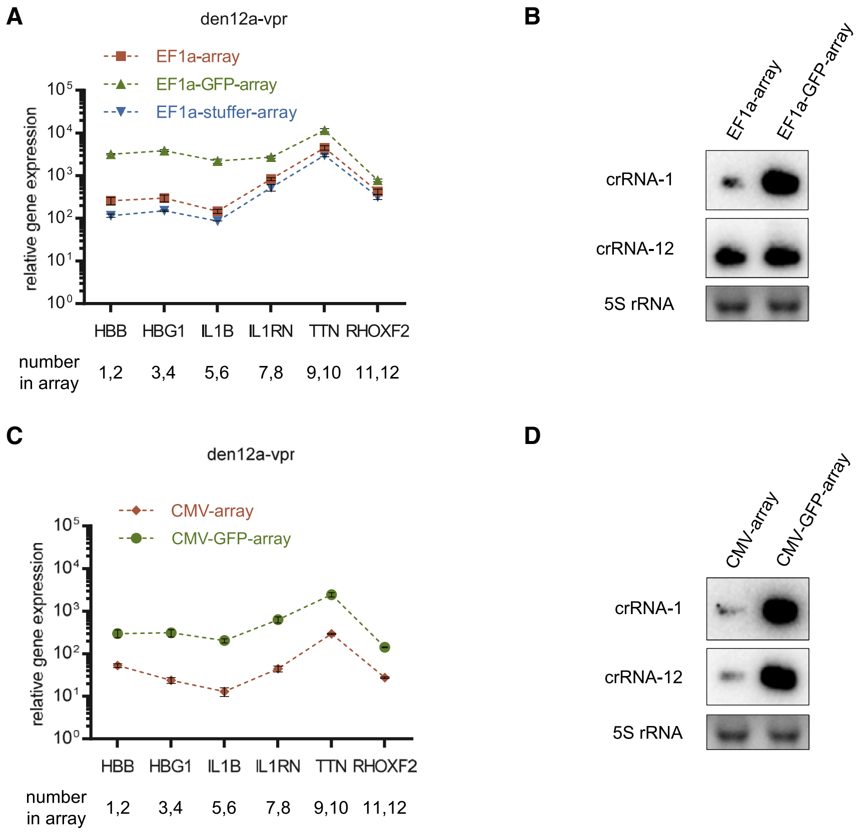

Figure 7. Enhanced expression of Pol II promoter-driven CRISPR arrays by introducing an upstream GFP-coding sequence

(A) Quantification of relative mRNA expression over the non-targeting control in HEK293T cells 48 h after transfection with EF1a-den12a-VPR and EF1a-driven arrays with or without upstream EGFP or stuffer. The 745-bp stuffer with a GC content of approximately 30% was cloned from the pLKO.1 cloning vector (see supplemental table for a detailed sequence). Values are shown as mean ± SD with n = 3. (B) Representative northern blot images of mature crRNAs processed from EF1a- and EF1a-GFP-arrays. (C) Quantification of relative mRNA expression over the non-targeting control in HEK293T cells 48 h after transfection with EF1a-den12a-VPR and CMV-driven arrays with/without upstream EGFP. Values shown as mean ± SD with n = 3. (D) Representative northern blot images of mature crRNAs processed from CMV- and CMV-GFP arrays.

random stuffer (Figure 7A). Northern blots of mature crRNAs verified an increase in expression levels, especially in the initial part (Figure 7B). This phenomenon is not unique, as CMV-driven CRISPR arrays can also be enhanced using this approach (Figures 7C and 7D). Moreover, enhanced CRISPRa efficiency was achieved by increasing the ratio of array-coding plasmids to those expressing the den12a-VPR (Figure S21A). Even the original version (i.e., den12a-VPR), which is supposed to possess considerably lower activity and requires a smaller number of crRNAs, showed improved CRISPRa efficiency when the ratio of the EF1a-array was increased or combined with the EF1a-GFP-array (Figure S21B).

Regarding the additional insertion of Triplex or WPRE when co-expressing den12a-VPR and CRISPR arrays on a single transcript, WPRE seemed to slightly rescue den12a-VPR mRNA/protein, whereas Triplex did not (Figures S20B and S20C). However, neither improved the efficiency of CRISPRa compared with direct integration (Figure S20D).

Taken together, the strategy of co-expressing the Cas protein and CRISPR array on a single transcript may be a viable option for some CRISPR systems under certain circumstances, but not always. According to our results, an important determinant is that the optimal ratio of the Cas effector to its crRNA varies across CRISPR systems. A systematic and rigorous evaluation is needed before applying this approach to other CRISPR systems not tested here, especially for *in vivo* delivery, which was lacking in our study.

DISCUSSION

Although up to 15 crRNAs can be assembled in a single reaction using our method, some previous studies have used arrays containing more than 20 crRNAs.[37] However, these methods typically perform more than one round of assembly or rely on expensive long dsDNA segments purchased from companies. Based on purified construct coding the array of 12 crRNAs assembled in the first round (while the assembly product of the first round was used as template) (Figure S16), a second round of PCR and subsequent assembly we performed (Figure S22A). Although recombination events occurred during clonal expansion, because the same template was used for all three PCR reactions, the array of 36 crRNAs was efficiently assembled with high accuracy (Figures S22B and S22C). Owing to the lack of appropriate application examples, we did not determine the maximum number of crRNAs that could be assembled in two rounds of assembly. Given the remarkable accuracy of assembling 36 crRNAs, the successful assembly of more crRNAs (i.e., 40, 50, or even more) within two rounds is possible.

Speaking of unwanted recombination events during the transformation of plasmids into competent *E. coli*, we were puzzled by its elusiveness. For convenience, even for the transformation of lentiviral constructs, we routinely use DH5α, a commonly used recA1 mutant competent *E. coli* strain, instead of stbl3 or NEB stable, which are supposed to further reduce recombination events between repeat elements in the plasmid. A lower temperature of 30°C for bacterial culture is also rarely used. However, we had never encountered any recombination events between the two long terminal repeats (LTRs) in any the lentiviral constructs. Specific to the current study, the accuracy of the assembly of

crRNAs was not improved by using stbl3 or NEB stable, nor by culturing at 30°C (data not shown). Compared with the transformation of the newly assembled mixture, recombination events were rare during the re-transformation of repeat-containing plasmids extracted from bacteria (data not shown). We are not sure whether most of the negative colonies were the result of unwanted recombination or simply failed assembly. As the reagents associated with Golden Gate cloning have been continuously upgraded over the years,[39,40] the limits on the maximum number of crRNAs that can be assembled in a single reaction, if the failure of assembly is currently the primary constraint, may be pushed further in the future.

In contrast with previous reports,[24,37] the current results indicate that EF1a is not functionally equivalent to U6 in the transcription of CRISPR arrays or single crRNA. Despite the differences between their strengths when transcribing transcripts of varying lengths, additional modifications (e.g., 7-methylguanosine cap and poly(A) tail) may also affect the subcellular localization or processing of the CRISPR array, thereby affecting its final execution. We attempted to quantitatively compare the transcription levels of the precursor crRNA arrays using RT-qPCR (Figure S23). To generate amplified segments of the appropriate length, each primer pair must span three to four crRNAs. This intrinsic property causes the final result to be affected by the positions of both the forward and reverse primers. For these RT-qPCR experiments, because we used reverse qPCR primers to produce cDNA in the reverse transcription reaction, the quantitative results were dominated by the expression level of the reverse primer locus. This may not be appropriate for the CRISPR array driven by U6, because the most abundant short transcripts containing only one or two crRNAs were not detected.

While the loss of the poly(A) tail by different approaches led to reduced EGFP expression, we noticed that the reduction seemed to be more drastic when the loss of the poly(A) tail was caused by processing the downstream CRISPR array, compared with simply removing the SV40-poly(A) signal in the construct (Figure S19). Although we are unsure whether the former accurately represents the actual effect of poly(A) deficiency, the latter may underestimate it. When SV40-poly(A) was removed from the construct, Pol II promoters did not stop transcribing upon completion of EGFP transcription, but instead continued to transcribe uncertain downstream sequences until terminated for other reasons. Thus, the 3′ terminus of the final EGFP mRNA was not directly exposed, but was protected by downstream RNA of uncertain length, which may delay the degradation of EGFP mRNA by acting as a stuffer. In lentiviral constructs, the poly(A) signal between the two LTRs must be removed, as it would lead to the premature termination of viral RNA during virus packaging. Consequently, the sequence integrated into the host genome after lentiviral infection does not have a poly(A) signal. A WPRE element is frequently introduced upstream of the 3′ LTR, which is supposed to stabilize and facilitate the translation of mRNA.[41] Whereas the improvement in the expression of upstream coding genes in our previous results with transient expression was mild (Figure S19), WPRE markedly enhanced the expression of upstream EGFP in the context of lentiviral delivery, which is consistent with the conventional concept. In contrast, the triplexes failed under these circumstances (Figure S23).

Although the previously reported co-expression strategy inevitably resulted in reduced expression of the Cas effector in our re-evaluation, it did not necessarily lead to compromised CRISPR efficiency, at least in the denAsCas12a-VPR mediated multiplex activation system. However, regarding the genomic DNA-targeting system with the primordial nuclease-active AsCas12a (or enAsCas12a), it is currently unknown whether this particular co-expression approach is compatible.

One of the shortcomings of this study, which we must emphasize, is that all the evaluations were performed *in vitro* using cell lines, and these results may not precisely represent the *in vivo* performance of the CRISPR systems and expression methods assessed here. Therefore, a rigorous *in vivo* evaluation is warranted before further application, especially when harnessing Pol II promoters to express CRISPR arrays or when using the co-expression strategy. The findings will substantially simplify the preparatory work before *in vivo* manipulation of multiple targets and boost the exploration of potential applications using multiplex CRISPR.

MATERIALS AND METHODS

Plasmid construction

The coding sequences of AsCas12a, VPR, and RfxCas13d were amplified from pY108 (Addgene #84739), pXR001 (#84739), and lenti-EF1a-dCas9-VPR-Puro (#99373) plasmids, respectively. The amplified fragments were cloned into destination vectors using standard digestion-ligation or the Gibson method. Gibson cloning was used to introduce the desired mutations into protein-coding plasmids. The CRISPR arrays were assembled using direct ligation or the standard Golden Gate method. The junction sets of the four-base overhangs were determined based on their ligation fidelity predicted by the online tool NEBridge Ligase Fidelity Viewer (https://ggtools.neb.com/viewset/run.cgi).[39] Detailed protocols are provided in the supplemental methods. All constructs, including those encoding the CRISPR arrays used in the editing experiments, were validated using Sanger sequencing. The detailed sequences of these constructs are listed in Table S22.

Assembly of CRISPR arrays with GGA-based strategy

Detailed protocols can be found in the supplemental methods. Briefly, PCR was performed using predesigned oligo pairs (synthesized by Tsingke Biotech). Next, an optional recovery step was performed, if necessary. The GGA reaction was set up with purified segments (or diluted PCR products) and a destination cloning vector. The reagents used were BsaI-HF v2 (NEB, R3733) and T4 DNA Ligase (NEB, M0202). The GGA program was run using a thermocycler: (37°C 5 min → 16°C 5 min) × 30 cycles, followed by 60°C for 5 min. If reactions were performed overnight, a 4°C terminal hold was added to the program and the step at 60°C for 5 min until the day before transformation. An amount of 2–10 μL of the assembly reaction was transformed into competent cells.

Cell culture and transient transfection

HEK293T cells were cultured in DMEM supplemented with 10% fetal bovine serum (FBS) and 1% penicillin/streptomycin at 37°C with 5% CO_2. For transient transfection, 2×10^5 HEK293T cells/well were seeded in 24-well plates. When 60%–80% confluence was reached the following day, a total of 500 ng plasmids were transfected into each well using Lipo8000 Transfection Reagent (Beyotime) according to the manufacturer's protocol. Transfected cells were supplied with fresh, complete DMEM (containing FBS) every 24 h, and harvested 48–72 h after transfection for downstream experiments.

Lentivirus production and infection

For lentivirus packaging, 5×10^5 HEK293T cells were seeded per well into 12-well plates. When 80% confluence was reached 80% the following day, 1 μg of mixed plasmids (transfer: psPAX2: pMD2.G = 4:3:1) were transfected into each well using Lipo8000 (Beyotime). The medium was replaced with 1 mL fresh, complete DMEM 12–24 h after transfection. Then, 48 h after transfection, another 1 mL of complete DMEM was added. At 72 h after transfection, lentivirus-containing supernatant was harvested, filtered through a 0.45-μm filter (or centrifuged at 2,000×*g* for 5 min) and stored at 4°C (or −80°C for long-term preservation).

For lentivirus infection, 1.5×10^5 HEK293T cells were seeded per well into 24-well plates. When 40%–60% confluence was reached the following day, the medium was replaced with 0.5 mL fresh complete DMEM and 1.5 mL lentivirus-containing supernatant. The medium was replaced with fresh, complete DMEM 12–24 h after infection and refreshed/supplemented every 24 h until cells were harvested 72 h after infection.

Extraction of RNA and RT-qPCR

Total RNA was extracted using RNAiso Plus (Takara) 48–72 h after transient transfection or lentiviral infection. An amount of 1 μg total RNA was then reverse transcribed using ReverTra Ace qPCR RT Kit (Toyobo) with supplied primer mix or gene-specific primers (when designated, and sequences are listed in Table S2), followed by qPCR using ChamQ Universal SYBR qPCR Master Mix (Vazyme) and the primers listed in Table S1. The qPCR was performed using a LightCycler 480 II (Roche). Quantification of RNA expression was normalized to ACTB (unless otherwise specified) and calculated using the ΔΔCt method.

Electrophoresis of oligonucleotides

DNA oligonucleotide electrophoresis was performed on a 20% polyacrylamide gel in 1× TBE buffer. The gel was stained with Gel Red (Beyotime) for 1 h at room temperature and scanned using an ultraviolet transilluminator after washing.

Quantification of gene editing

A total of 72 h after transfection, genomic DNA was extracted using a Rapid Animal Genomic DNA Isolation Kit (Sangon Biotech) following the manufacturer's protocol. The extracted DNA was used as a template to amplify the target region flanking the edited site, using specific primers. The PCR amplicons were purified using the AxyPrep PCR Clean-up Kit (Axygen) following the manufacturer's protocol. Purified DNA was subjected to Sanger sequencing using the forward PCR primer as the sequencing primer. The resulting ".ab1" files were uploaded to obtain the final quantitative spectrum of the indels using the online tool: https://ice.synthego.com.[42]

Northern blot

Northern blots of mature crRNAs were based on the Bioton-Streptavidin system and performed using the Biotin Northern Blot Kit (for Small RNA) (Beyotime, R0220) according to the manufacturer's protocol. The 5′ Bioton-labelled DNA probes were synthesized by Tsingke Biotech. The probe sequences are listed in Table S21. The 5s rRNA was used as an internal control and was detected by agarose gel electrophoresis.

Western blot

A total of 72 h after transient transfection or lentivirus infection, cells were lysed in RIPA buffer (MedChem Express) supplemented with PMSF and BeyoZonase Super Nuclease (Beyotime). Total protein concentration was quantitated using Pierce BCA Protein Assay Kit (Thermo Fisher Scientific). All lysates were diluted to a concentration of 1,250 ng/μL, then mixed with 5× loading buffer (a final protein concentration of 1 μg/μL) and boiled for 5 min. Equal amounts of protein were loaded and separated using sodium dodecyl sulphate-polyacrylamide gel electrophoresis and then transferred to a polyvinylidene difluoride membrane. Membranes were blocked with 5% nonfat milk in TBST buffer for 1 h at room temperature, and incubated with appropriate dilutions of primary antibody overnight at 4°C. The next day, the membrane was washed three times with TBST and incubated with a horseradish peroxidase-conjugated secondary antibody for 1 h at room temperature. After three washes, chemiluminescent signals in the membrane were captured using a CCD camera.

Statistical analysis

Values are reported as mean or mean ± SD as indicated in the figure legends. Unless otherwise stated, at least three biological replicates were used for each experiment. When comparing two groups, statistical differences were determined using the unpaired Student's t test. One-way ANOVA was used to assess the significance of differences between more than two groups. Two-way analysis of variance was used to compare two factors. Statistical significance was set at a p value of less than 0.05. Prism 6.01 was used for all statistical analyses.

DATA AND CODE AVAILABILITY

The authors declare that the data supporting the results of this study are available in the article and supplemental material. Additional resource information is available from the corresponding author upon request.

ACKNOWLEDGMENTS

We thank the members of the Institute of Immunology (Zhejiang University School of Medicine) for their support in this study. This work was financially supported by National Natural Science Foundation of China (32300642 and 82102814), Zhejiang Provincial Natural Science Foundation of China (LQ24C060009 and LQ22H160053), and

postdoctoral grants for scientific research from the Zhejiang Provincial People's Hospital (C-2023-BSH28).

AUTHOR CONTRIBUTIONS

X.Z.: Conception and design, collection of data, data analysis, manuscript writing. L.Y.: Conception and design, data collection, data analysis, manuscript writing, financial support. P.L., Z.C., Y.J., L.L., A.B., H.Xiong., H.Z., H.Xu., and J.Z.: Collection of data, data analysis, manuscript editing, administrative, technical, material, or financial support. Y.Z.: Conception and design, data analysis, financial support, revision, and final approval of the manuscript.

DECLARATION OF INTERESTS

The authors declare no competing interests.

SUPPLEMENTAL INFORMATION

Supplemental information can be found online at https://doi.org/10.1016/j.omtn.2024.102428.

REFERENCES

1. Jinek, M., Chylinski, K., Fonfara, I., Hauer, M., Doudna, J.A., and Charpentier, E. (2012). A programmable dual-RNA-guided DNA endonuclease in adaptive bacterial immunity. Science *337*, 816–821.
2. Cong, L., Ran, F.A., Cox, D., Lin, S., Barretto, R., Habib, N., Hsu, P.D., Wu, X., Jiang, W., Marraffini, L.A., and Zhang, F. (2013). Multiplex genome engineering using CRISPR/Cas systems. Science *339*, 819–823.
3. Mali, P., Yang, L., Esvelt, K.M., Aach, J., Guell, M., DiCarlo, J.E., Norville, J.E., and Church, G.M. (2013). RNA-guided human genome engineering via Cas9. Science *339*, 823–826.
4. Makarova, K.S., Wolf, Y.I., Iranzo, J., Shmakov, S.A., Alkhnbashi, O.S., Brouns, S.J.J., Charpentier, E., Cheng, D., Haft, D.H., Horvath, P., et al. (2020). Evolutionary classification of CRISPR-Cas systems: a burst of class 2 and derived variants. Nat. Rev. Microbiol. *18*, 67–83.
5. Abudayyeh, O.O., Gootenberg, J.S., Konermann, S., Joung, J., Slaymaker, I.M., Cox, D.B.T., Shmakov, S., Makarova, K.S., Semenova, E., Minakhin, L., et al. (2016). C2c2 is a single-component programmable RNA-guided RNA-targeting CRISPR effector. Science *353*, aaf5573.
6. East-Seletsky, A., O'Connell, M.R., Knight, S.C., Burstein, D., Cate, J.H.D., Tjian, R., and Doudna, J.A. (2016). Two distinct RNase activities of CRISPR-C2c2 enable guide-RNA processing and RNA detection. Nature *538*, 270–273.
7. Abudayyeh, O.O., Gootenberg, J.S., Essletzbichler, P., Han, S., Joung, J., Belanto, J.J., Verdine, V., Cox, D.B.T., Kellner, M.J., Regev, A., et al. (2017). RNA targeting with CRISPR-Cas13. Nature *550*, 280–284.
8. Cox, D.B.T., Gootenberg, J.S., Abudayyeh, O.O., Franklin, B., Kellner, M.J., Joung, J., and Zhang, F. (2017). RNA editing with CRISPR-Cas13. Science *358*, 1019–1027.
9. Konermann, S., Lotfy, P., Brideau, N.J., Oki, J., Shokhirev, M.N., and Hsu, P.D. (2018). Transcriptome Engineering with RNA-Targeting Type VI-D CRISPR Effectors. Cell *173*, 665–676.e14.
10. Ozcan, A., Krajeski, R., Ioannidi, E., Lee, B., Gardner, A., Makarova, K.S., Koonin, E.V., Abudayyeh, O.O., and Gootenberg, J.S. (2021). Programmable RNA targeting with the single-protein CRISPR effector Cas7-11. Nature *597*, 720–725.
11. Colognori, D., Trinidad, M., and Doudna, J.A. (2023). Precise transcript targeting by CRISPR-Csm complexes. Nat. Biotechnol. *41*, 1256–1264.
12. Konermann, S., Brigham, M.D., Trevino, A.E., Joung, J., Abudayyeh, O.O., Barcena, C., Hsu, P.D., Habib, N., Gootenberg, J.S., Nishimasu, H., et al. (2015). Genome-scale transcriptional activation by an engineered CRISPR-Cas9 complex. Nature *517*, 583–588.
13. Gilbert, L.A., Horlbeck, M.A., Adamson, B., Villalta, J.E., Chen, Y., Whitehead, E.H., Guimaraes, C., Panning, B., Ploegh, H.L., Bassik, M.C., et al. (2014). Genome-Scale CRISPR-Mediated Control of Gene Repression and Activation. Cell *159*, 647–661.
14. Anzalone, A.V., Koblan, L.W., and Liu, D.R. (2020). Genome editing with CRISPR-Cas nucleases, base editors, transposases and prime editors. Nat. Biotechnol. *38*, 824–844.
15. Hilton, I.B., D'Ippolito, A.M., Vockley, C.M., Thakore, P.I., Crawford, G.E., Reddy, T.E., and Gersbach, C.A. (2015). Epigenome editing by a CRISPR-Cas9-based acetyltransferase activates genes from promoters and enhancers. Nat. Biotechnol. *33*, 510–517.
16. Anzalone, A.V., Randolph, P.B., Davis, J.R., Sousa, A.A., Koblan, L.W., Levy, J.M., Chen, P.J., Wilson, C., Newby, G.A., Raguram, A., and Liu, D.R. (2019). Search-and-replace genome editing without double-strand breaks or donor DNA. Nature *576*, 149–157.
17. Tanenbaum, M.E., Gilbert, L.A., Qi, L.S., Weissman, J.S., and Vale, R.D. (2014). A protein-tagging system for signal amplification in gene expression and fluorescence imaging. Cell *159*, 635–646.
18. Ma, H., Marti-Gutierrez, N., Park, S.W., Wu, J., Lee, Y., Suzuki, K., Koski, A., Ji, D., Hayama, T., Ahmed, R., et al. (2017). Correction of a pathogenic gene mutation in human embryos. Nature *548*, 413–419.
19. Wang, D., Li, J., Song, C.Q., Tran, K., Mou, H., Wu, P.H., Tai, P.W.L., Mendonca, C.A., Ren, L., Wang, B.Y., et al. (2018). Cas9-mediated allelic exchange repairs compound heterozygous recessive mutations in mice. Nat. Biotechnol. *36*, 839–842.
20. Beyret, E., Liao, H.K., Yamamoto, M., Hernandez-Benitez, R., Fu, Y., Erikson, G., Reddy, P., and Izpisua Belmonte, J.C. (2019). Single-dose CRISPR-Cas9 therapy extends lifespan of mice with Hutchinson-Gilford progeria syndrome. Nat. Med. *25*, 419–422.
21. Maeder, M.L., Stefanidakis, M., Wilson, C.J., Baral, R., Barrera, L.A., Bounoutas, G.S., Bumcrot, D., Chao, H., Ciulla, D.M., DaSilva, J.A., et al. (2019). Development of a gene-editing approach to restore vision loss in Leber congenital amaurosis type 10. Nat. Med. *25*, 229–233.
22. Nelson, C.E., Wu, Y., Gemberling, M.P., Oliver, M.L., Waller, M.A., Bohning, J.D., Robinson-Hamm, J.N., Bulaklak, K., Castellanos Rivera, R.M., Collier, J.H., et al. (2019). Long-term evaluation of AAV-CRISPR genome editing for Duchenne muscular dystrophy. Nat. Med. *25*, 427–432.
23. Santiago-Fernandez, O., Osorio, F.G., Quesada, V., Rodriguez, F., Basso, S., Maeso, D., Rolas, L., Barkaway, A., Nourshargh, S., Folgueras, A.R., et al. (2019). Development of a CRISPR/Cas9-based therapy for Hutchinson-Gilford progeria syndrome. Nat. Med. *25*, 423–426.
24. Kleinstiver, B.P., Sousa, A.A., Walton, R.T., Tak, Y.E., Hsu, J.Y., Clement, K., Welch, M.M., Horng, J.E., Malagon-Lopez, J., Scarfò, I., et al. (2019). Engineered CRISPR-Cas12a variants with increased activities and improved targeting ranges for gene, epigenetic and base editing. Nat. Biotechnol. *37*, 276–282.
25. Mali, P., Aach, J., Stranges, P.B., Esvelt, K.M., Moosburner, M., Kosuri, S., Yang, L., and Church, G.M. (2013). CAS9 transcriptional activators for target specificity screening and paired nickases for cooperative genome engineering. Nat. Biotechnol. *31*, 833–838.
26. Maeder, M.L., Linder, S.J., Cascio, V.M., Fu, Y., Ho, Q.H., and Joung, J.K. (2013). CRISPR RNA-guided activation of endogenous human genes. Nat. Methods *10*, 977–979.
27. Cheng, A.W., Wang, H., Yang, H., Shi, L., Katz, Y., Theunissen, T.W., Rangarajan, S., Shivalila, C.S., Dadon, D.B., and Jaenisch, R. (2013). Multiplexed activation of endogenous genes by CRISPR-on, an RNA-guided transcriptional activator system. Cell Res. *23*, 1163–1171.
28. Perez-Pinera, P., Kocak, D.D., Vockley, C.M., Adler, A.F., Kabadi, A.M., Polstein, L.R., Thakore, P.I., Glass, K.A., Ousterout, D.G., Leong, K.W., et al. (2013). RNA-guided gene activation by CRISPR-Cas9-based transcription factors. Nat. Methods *10*, 973–976.
29. Zetsche, B., Gootenberg, J.S., Abudayyeh, O.O., Slaymaker, I.M., Makarova, K.S., Essletzbichler, P., Volz, S.E., Joung, J., van der Oost, J., Regev, A., et al. (2015). Cpf1 is a single RNA-guided endonuclease of a class 2 CRISPR-Cas system. Cell *163*, 759–771.
30. Fonfara, I., Richter, H., Bratovič, M., Le Rhun, A., and Charpentier, E. (2016). The CRISPR-associated DNA-cleaving enzyme Cpf1 also processes precursor CRISPR RNA. Nature *532*, 517–521.
31. DeWeirdt, P.C., Sanson, K.R., Sangree, A.K., Hegde, M., Hanna, R.E., Feeley, M.N., Griffith, A.L., Teng, T., Borys, S.M., Strand, C., et al. (2021). Optimization of AsCas12a for combinatorial genetic screens in human cells. Nat. Biotechnol. *39*, 94–104.

32. Xie, K., Minkenberg, B., and Yang, Y. (2015). Boosting CRISPR/Cas9 multiplex editing capability with the endogenous tRNA-processing system. Proc. Natl. Acad. Sci. USA *112*, 3570–3575.

33. Shao, S., Chang, L., Sun, Y., Hou, Y., Fan, X., and Sun, Y. (2018). Multiplexed sgRNA Expression Allows Versatile Single Nonrepetitive DNA Labeling and Endogenous Gene Regulation. ACS Synth. Biol. *7*, 176–186.

34. Breunig, C.T., Durovic, T., Neuner, A.M., Baumann, V., Wiesbeck, M.F., Köferle, A., Götz, M., Ninkovic, J., and Stricker, S.H. (2018). One step generation of customizable gRNA vectors for multiplex CRISPR approaches through string assembly gRNA cloning (STAgR). PLoS One *13*, e0196015.

35. Zhou, H., Huang, C., and Xia, X.G. (2008). A tightly regulated Pol III promoter for synthesis of miRNA genes in tandem. Biochim. Biophys. Acta *1779*, 773–779.

36. Xu, L., Zhao, L., Gao, Y., Xu, J., and Han, R. (2017). Empower multiplex cell and tissue-specific CRISPR-mediated gene manipulation with self-cleaving ribozymes and tRNA. Nucleic Acids Res. *45*, e28.

37. Campa, C.C., Weisbach, N.R., Santinha, A.J., Incarnato, D., and Platt, R.J. (2019). Multiplexed genome engineering by Cas12a and CRISPR arrays encoded on single transcripts. Nat. Methods *16*, 887–893.

38. Passmore, L.A., and Coller, J. (2022). Roles of mRNA poly(A) tails in regulation of eukaryotic gene expression. Nat. Rev. Mol. Cell Biol. *23*, 93–106.

39. Potapov, V., Ong, J.L., Kucera, R.B., Langhorst, B.W., Bilotti, K., Pryor, J.M., Cantor, E.J., Canton, B., Knight, T.F., Evans, T.C., Jr., and Lohman, G.J.S. (2018). Comprehensive Profiling of Four Base Overhang Ligation Fidelity by T4 DNA Ligase and Application to DNA Assembly. ACS Synth. Biol. *7*, 2665–2674.

40. Pryor, J.M., Potapov, V., Kucera, R.B., Bilotti, K., Cantor, E.J., and Lohman, G.J.S. (2020). Enabling one-pot Golden Gate assemblies of unprecedented complexity using data-optimized assembly design. PLoS One *15*, e0238592.

41. Zufferey, R., Donello, J.E., Trono, D., and Hope, T.J. (1999). Woodchuck hepatitis virus posttranscriptional regulatory element enhances expression of transgenes delivered by retroviral vectors. J. Virol. *73*, 2886–2892.

42. Conant, D., Hsiau, T., Rossi, N., Oki, J., Maures, T., Waite, K., Yang, J., Joshi, S., Kelso, R., Holden, K., et al. (2022). Inference of CRISPR Edits from Sanger Trace Data. CRISPR J. *5*, 123–130.

OMTN, Volume *36*

Supplemental information

High-accuracy crRNA array assembly strategy

for multiplex CRISPR

Xiangtong Zhao, Lixian Yang, Peng Li, Zijing Cheng, Yongshi Jia, Limin Luo, Aihong Bi, Hanchu Xiong, Haibo Zhang, Hongen Xu, Jinrui Zhang, and Yaodong Zhang

Supplemental Figures

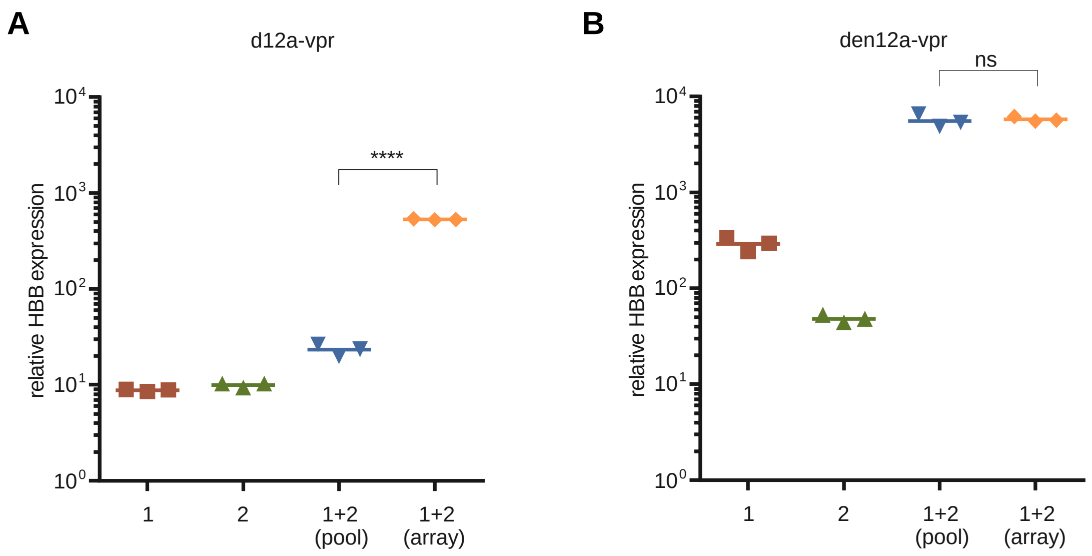

Figure S1. Synergistic transcriptional activation of *HBB* with d12a-VPR or den12a-VPR. **(A**, **B)** Quantification of relative *HBB* expression over non-targeting control in HEK293T cells 48h after transfection with plasmids encoding d12a-VPR **(A)** or den12a-VPR **(B)** and *HBB* promoter-targeting crRNA-1, crRNA-2, a pool of both, or an array containing both. Values shown as mean, n = 3 independent experiments.

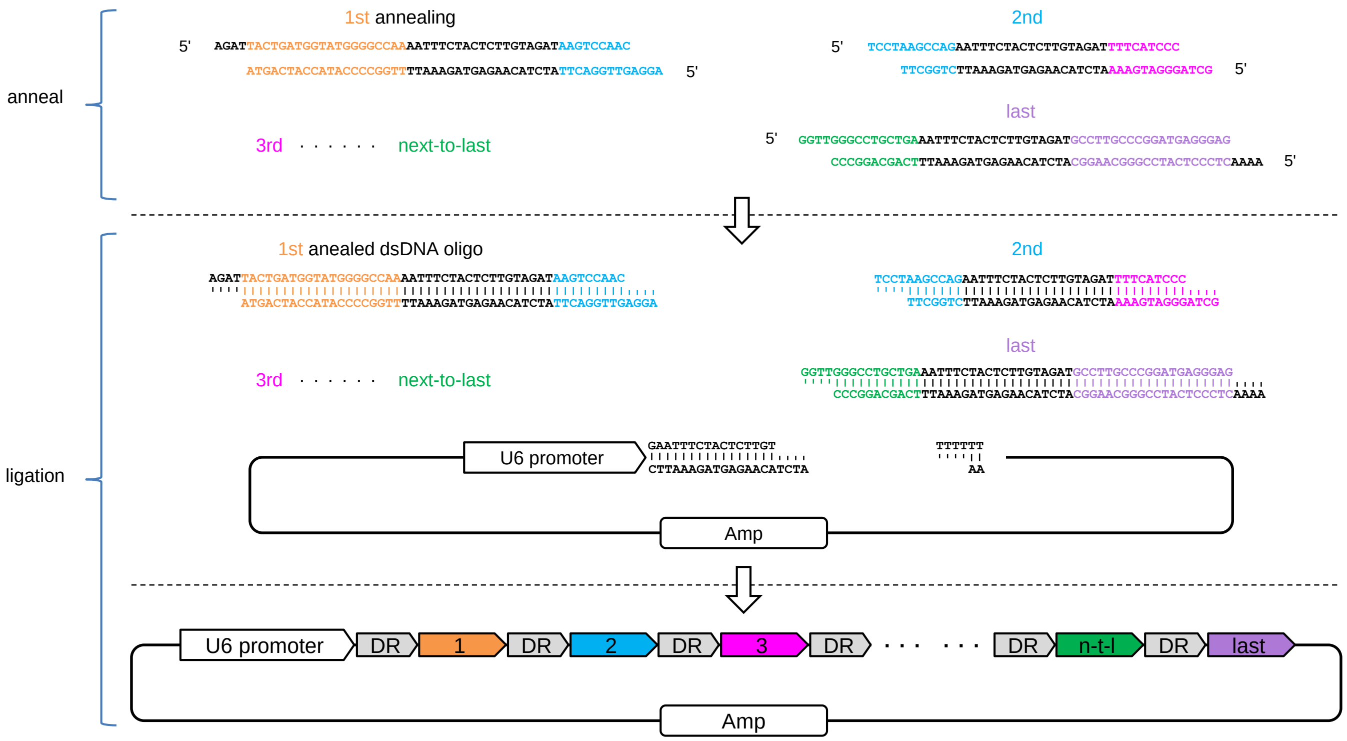

Figure S2. Schematic illustration and workflow of conventional sticky end-based CRISPR array assembly strategy. First, anneal single-stranded oligo pairs to form double-stranded DNA segments with desired sticky ends. Then, set up and run a ligation reaction with diluted annealed oligos and destination cloning vector (with/without predigestion and purification). Detailed protocols are provided in Supplemental Methods.

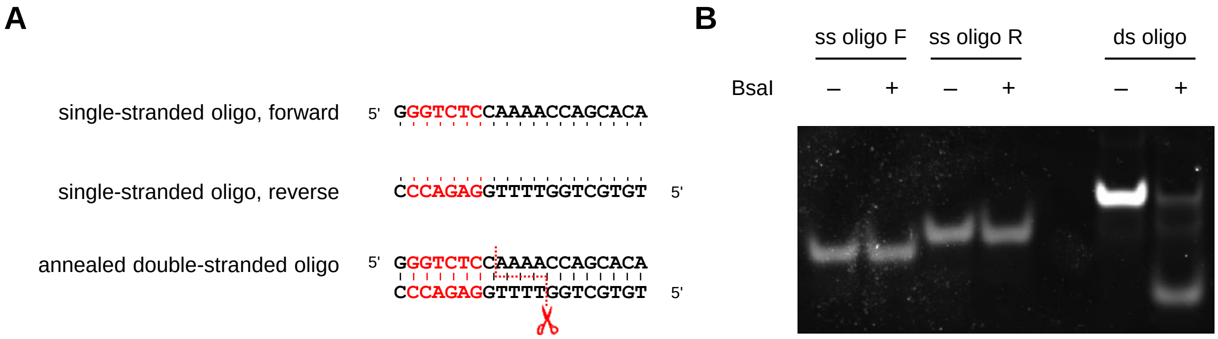

Figure S3. Restriction endonuclease BsaI specifically cleaves double-stranded DNA substrates. **(A)** Reverse complementary single-stranded oligos and resulting annealed double-stranded product. Recognition site of BsaI is colored red. Cleavage site is indicated by red dashed line. **(B)** Representative PAGE image of oligos depicted in (**A**) after incubation with/without BsaI at 37°C for 1 hour.

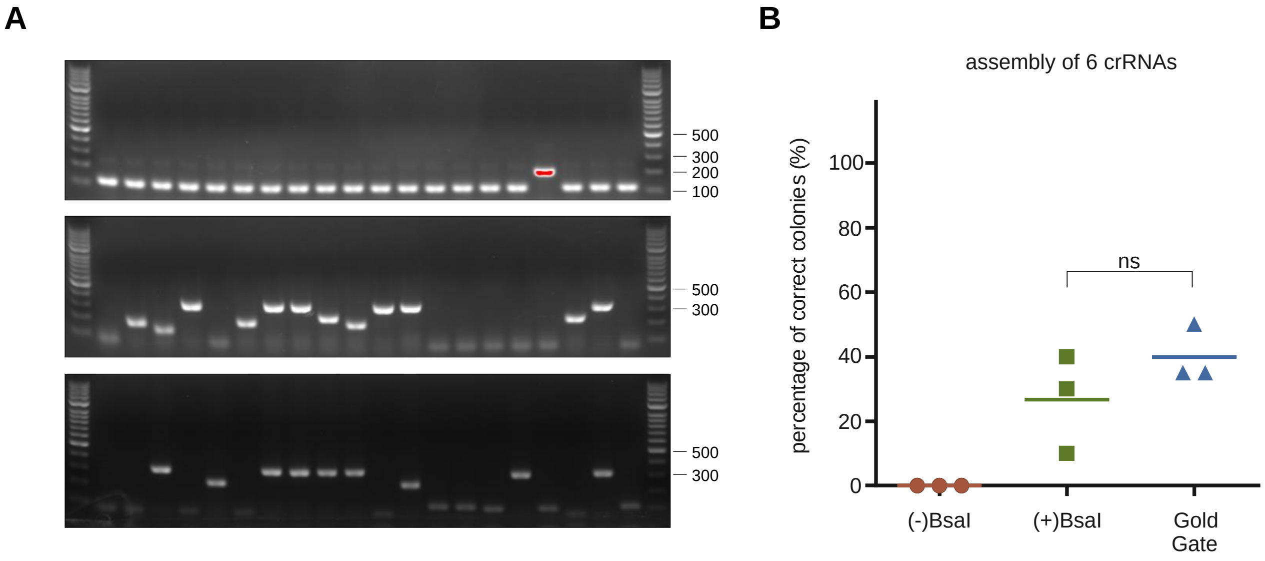

Figure S4. Comparison of assembly accuracy across different versions of conventional sticky end-based strategy. **(A)** Representative images of colony PCR to evaluate the accuracy of assembling 6 crRNAs (correct size ≈ 290 nt) using three versions of sticky end-based strategy: (-)BsaI (upper), (+)BsaI (middle), Gold Gate (lower), and the corresponding accuracies are shown in **(B)**. (-)BsaI: pre-digested cloning vector is recovered and added to a ligation reaction with annealed oligos, incubated overnight at room temperature, then directly transformed into competent cells; (+)BsaI: same as (-)BsaI, except that the ligation mixture is re-digested with BsaI for 15 minutes before transformation; Gold Gate: circular cloning vector and annealed oligos are added to a Gold Gate reaction, and then run a standard Gold Gate assembly program (37℃ 5 min → 16℃ 5 min, 30 cycles, followed by 60℃ 5 min) with a thermocycler. Detailed protocols are provided in Supplemental Methods. Values shown as mean, n = 3 independent experiments.

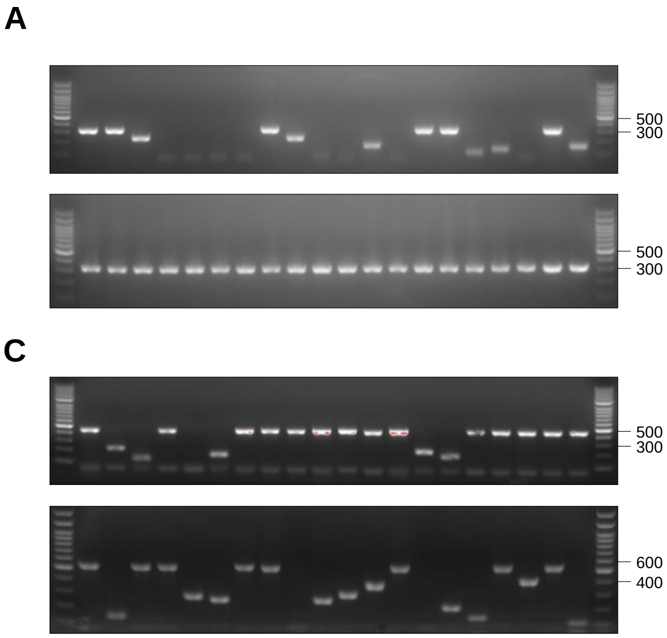

Figure S5. High-accuracy assembly of CRISPR array with GGA-based strategy. (**A**, **B**) Representative images of colony PCR to evaluate the accuracy of assembling 6 (**A**, correct size ≈ 290 nt) or 7 (**B**, correct size ≈ 330 nt) crRNAs using conventional sticky end-based (upper) or GGA-based strategy (lower). (**C**) Representative images of colony PCR to evaluate the accuracy of assembling 9 (upper, correct size ≈ 410 nt) or 12 (lower, correct size ≈ 530 nt) crRNAs using GGA-based strategy.

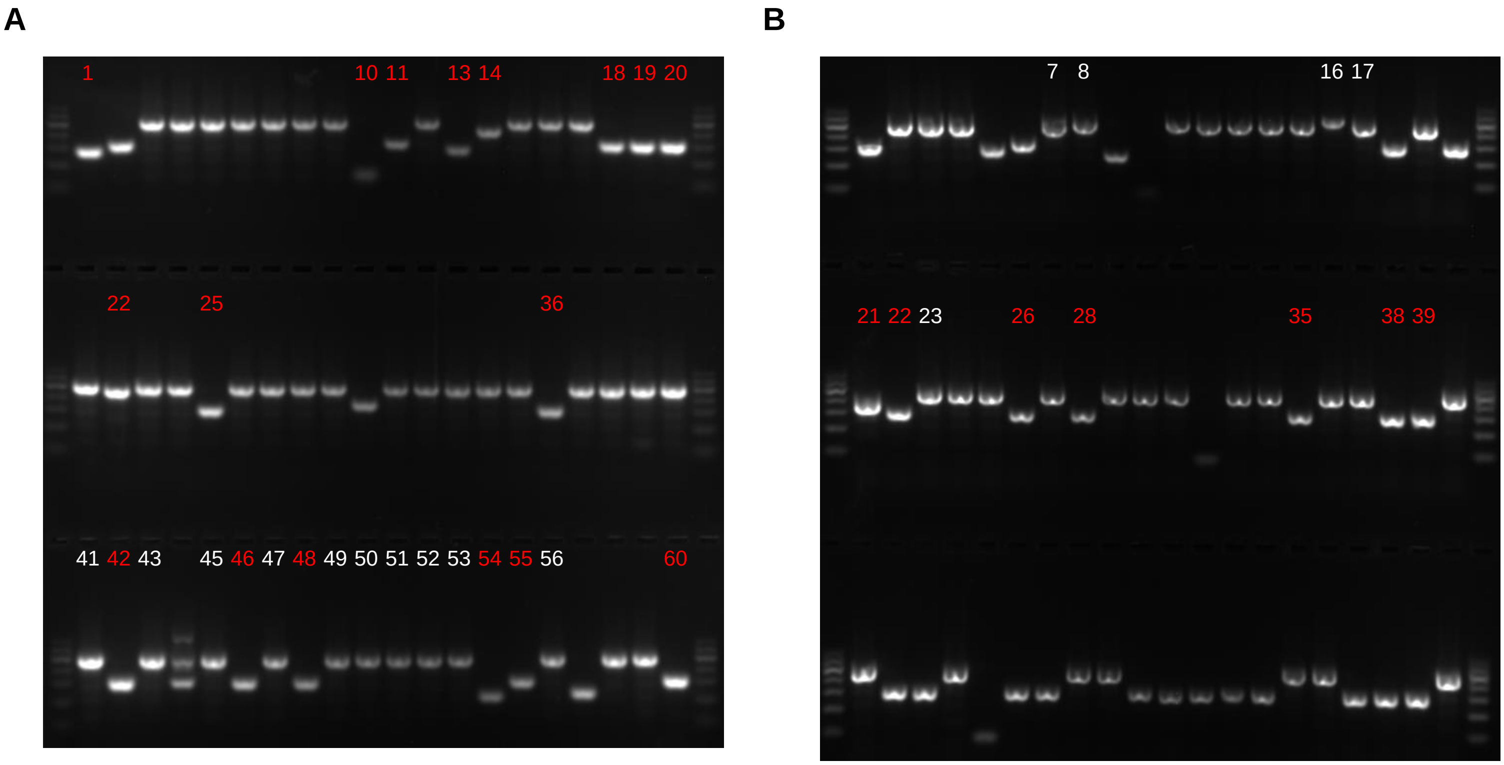

Figure S6. For the assembly of the 12 crRNAs, colonies from an additional replicate experiment were sequenced to confirm our results and investigate the source of errors in incorrect inserts during the revision of this manuscript. The 12×crRNA array presented here was based on the oligos used to assemble the reverse array shown in Figure S14A, as these oligos are closer to the manufacturing date. Note that these oligos were designed based on the mutant DR sequence (Figure 3C).

Colonies numbered with a white front indicated sequenced correctly sized colonies. All colonies except colony A-43 were sequenced to be correct (14/15). The sanger sequencing results in FASTA format were provided in the Table S37.

Colonies numbered with a red front indicated sequenced incorrectly sized colonies. Due to our limited capacity, we were barely able to find any clues to further mechanisms from the sequencing results, except that 15 of the 24 incorrectly sized colonies had the same wrong sequence: A (11, 18, 20, 25, 36, 42, 46, 55, 60), B (22, 26, 28, 35, 38, 39). And the sequence is: AATTCCTACTCTTGTAGGTGCCTTGCCCGGATGAGGGAGAATTCCTACTCTTGTAGGTCTGTGGG-
TTGGGCCTGCTGAAATTCCTACTCTTGTAGGTACATCAGCAACCAATGCTCTAATTCCTACTCTTGTAGGTTA
TGACTGCCCACAAGTGCTAATTCCTACTCTTGTAGGTAATGAATGTGTGCCAGCCAATTCCTACTCTTGTAGG
TAAGTCCAACTCCTAAGCCAGAATTCCTACTCTTGTAGGTTACTGATGGTATGGGGCCAA. The error site had been annotated as red underlined letters. It was an 18-nt hybrid of the following two 20-nt spacers: AATGAATGT**GTGC**ACACATG, TTCTTCATCCC**TAGC**CAGCC. The sanger sequencing results in FASTA format were provided in the Table S38.

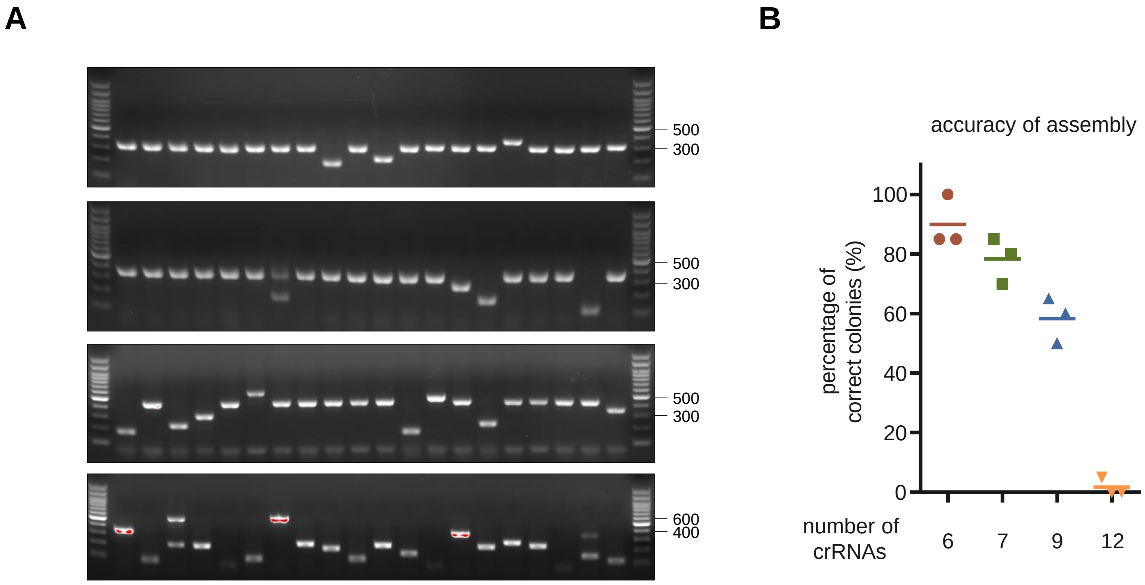

Figure S7. Accuracies of assembling 6 to 12 crRNAs using simplified recovery-free version of GGA-based strategy. (**A**) Representative images of colony PCR when assembling 6 (upper, correct size ≈ 290 nt), 7 (middle-upper, correct size ≈ 330 nt), 9 (middle-lower, correct size ≈ 410 nt), and 12 (lower, correct size ≈ 530 nt) crRNAs, and the corresponding accuracies are shown in **(B)**. Values shown as mean with n = 3.

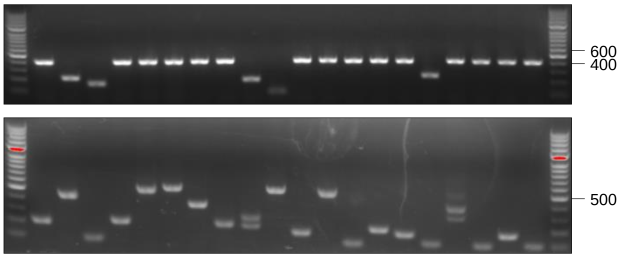

Figure S8. Representative images of colony PCR when assembling 9 (upper, correct size ≈ 410 nt) and 12 (lower, correct size ≈ 530 nt) crRNAs using mutant DR with higher GC content.

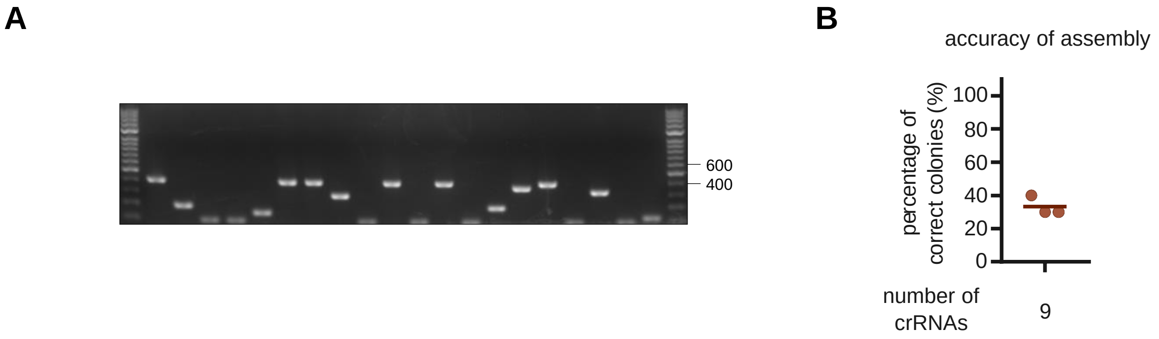

Figure S9. Accuracies of assembling 9 crRNAs (correct size ≈ 410 nt) using simplified recovery-free version of GGA-based strategy and mutant DR with higher GC content. (**A**) Representative image of colony PCR, and the corresponding accuracies are shown in **(B)**. Values shown as mean with n = 3.

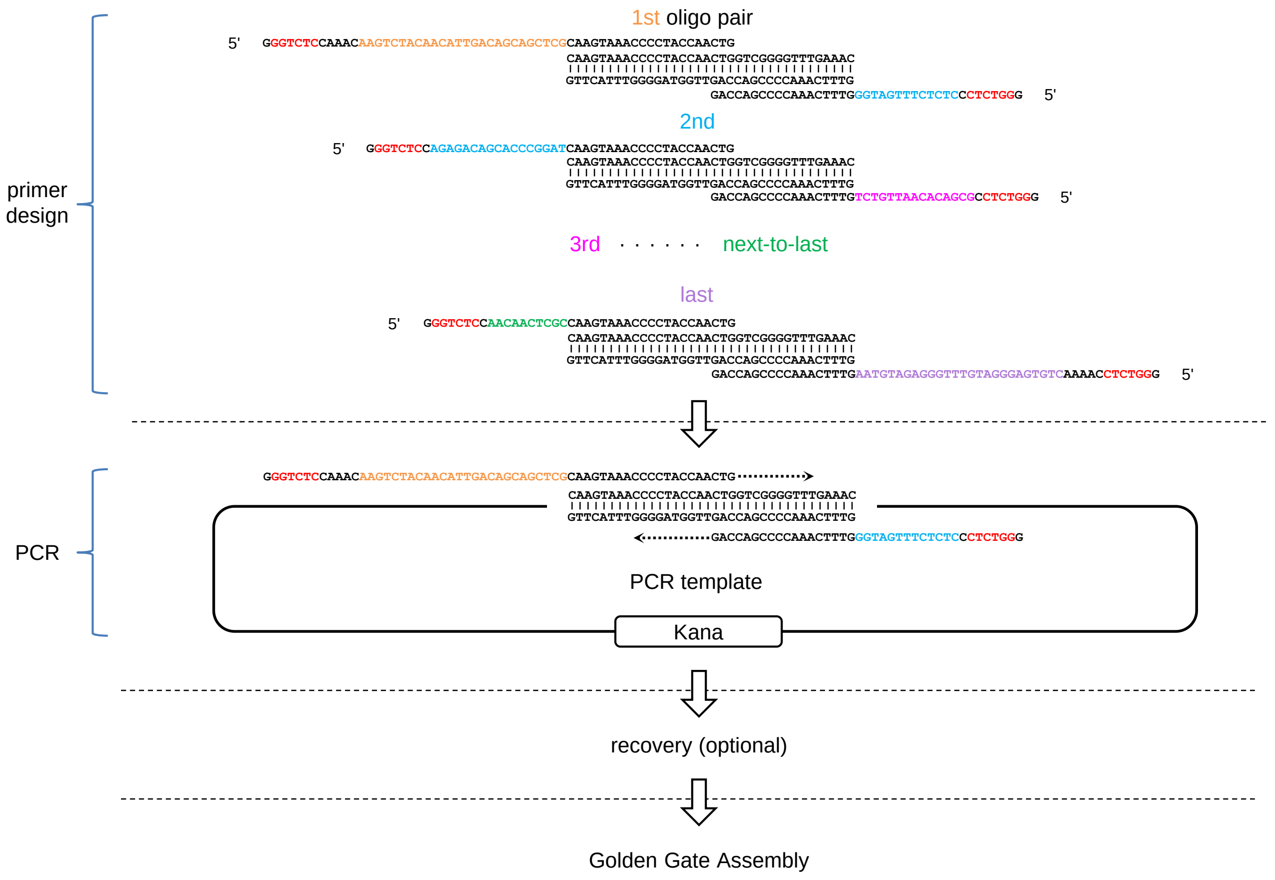

Figure S10. Schematic illustration and workflow of assembling crRNAs for RfxCas13d. Similar to Figure 1 except for the following minor modifications: (1) Primer pairs are designed to be partially (~20 bases 3' section) complementary to the 36bp DR of RfxCas13d, instead of to each other; (2) To further shorten the initial oligos for cost-saving, a kanamycin-resistant plasmid containing the 36nt DR of RfxCas13d, which could be easily home-made, is introduced as a template for PCR reactions; (3) The relatively long length of the PCR products (70~100nt) renders them compatible with common commercial DNA recovery kits, which are therefore used to obtain purer dsDNA segments.

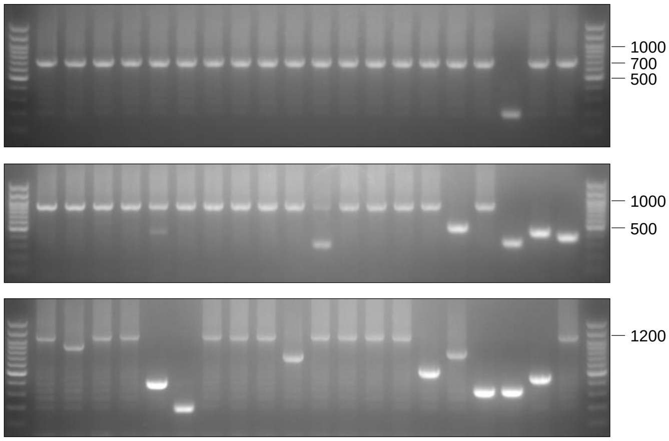

Figure S11. High-accuracy assembly of crRNAs for a Cas13d nuclease. Representative images of colony PCR to evaluate the accuracy of assembling 9 (upper, correct size ≈ 690 nt), 12 (middle, correct size ≈ 870 nt), or 15 (lower, correct size ≈ 1060 nt) crRNAs for RfxCas13d using GGA-based strategy.

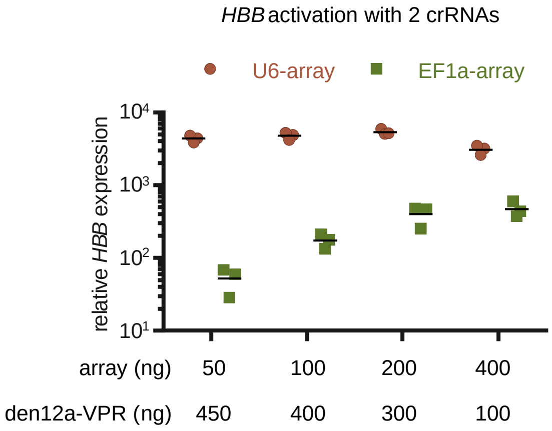

Figure S12. Quantification of relative *HBB* expression over non-targeting control in HEK293T cells 48h after transfection with the indicated amount of denAsCas12a-VPR and an array of 2 crRNAs targeting *HBB* promoter. Values shown as mean with n = 3.

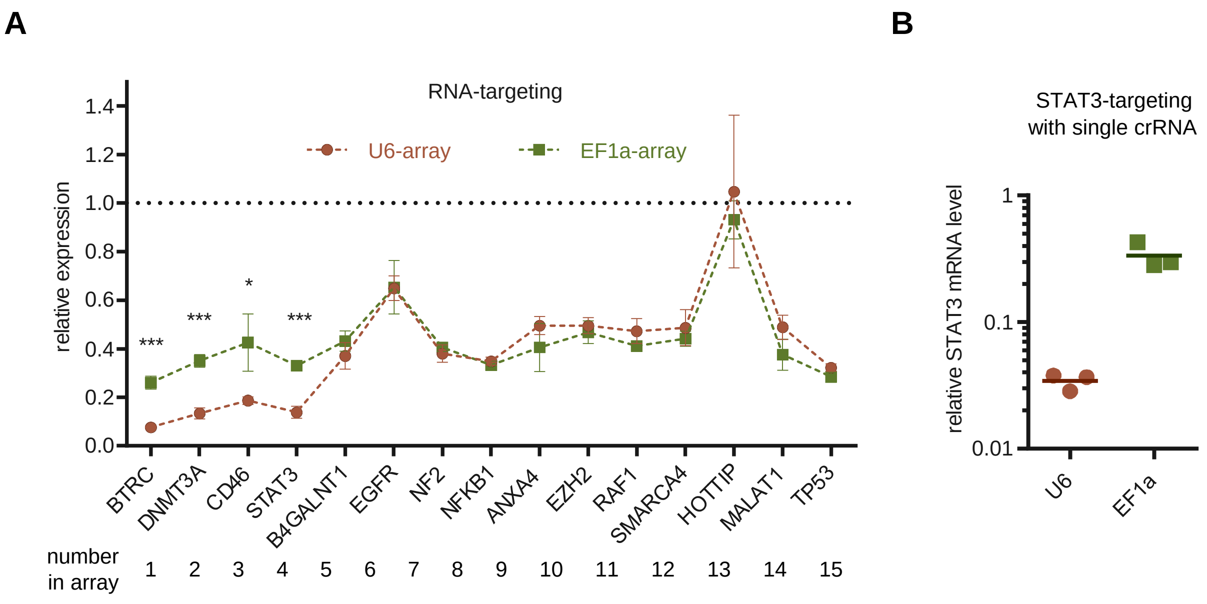

Figure S13. Distinct expression patterns of Pol II/III promoter-driven CRISPR arrays with *Rfx*Cas13d system. (**A**) Quantification of relative mRNA expression of the indicated genes compared to non-targeting control in HEK293T cells 48h after transfection with RfxCas13d and an array of 15 crRNAs driven by either U6 (U6-array) or EF1a (EF1a-array). Values shown as mean ± SD with n = 3. (**B**) Quantification of relative STAT3 mRNA expression compared to non-targeting control in HEK293T cells 48h after transfection with RfxCas13d and a STAT3-targeting crRNA driven by either U6 or EF1a. Values shown as mean with n = 3.

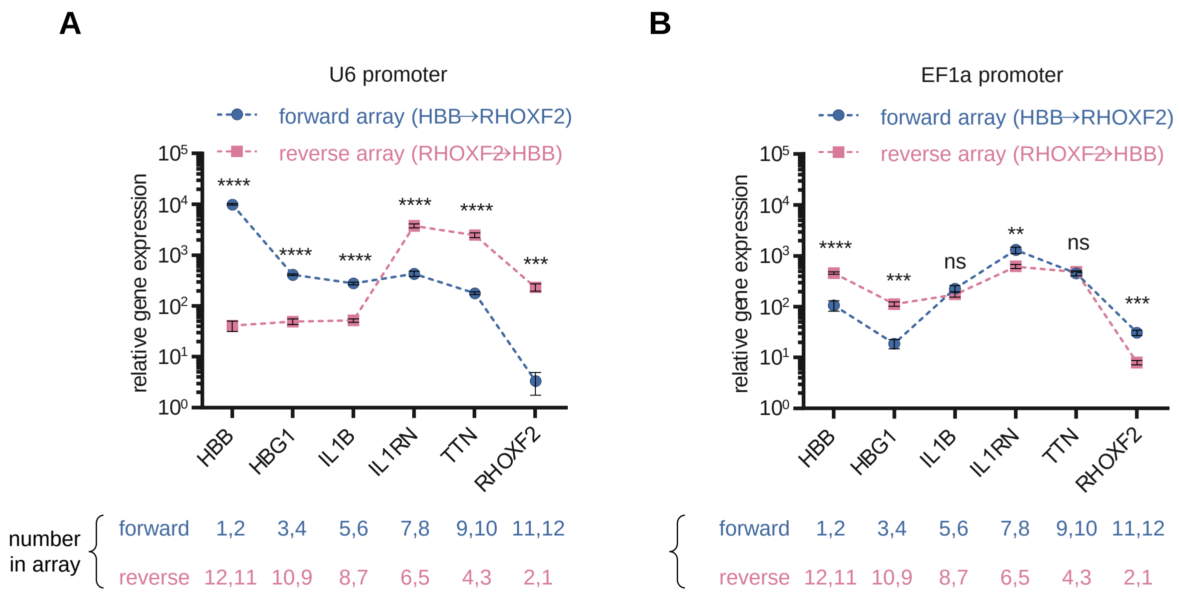

Figure S14. Transcription intensities across crRNAs located at different sites are milder but more evenly distributed in CRISPR arrays driven by Pol II promoters. (**A**) Quantification of relative mRNA expression over non-targeting control in HEK293T cells 48h after transfection with den12a-VPR and U6-driven arrays whose internal crRNAs are assembled in reverse order. (**B**) Quantification of relative mRNA expression over non-targeting control in HEK293T cells 48h after transfection with den12a-VPR and EF1a-driven arrays whose internal crRNAs are assembled in reverse order. Values shown as mean ± SD with n = 3.

A

B

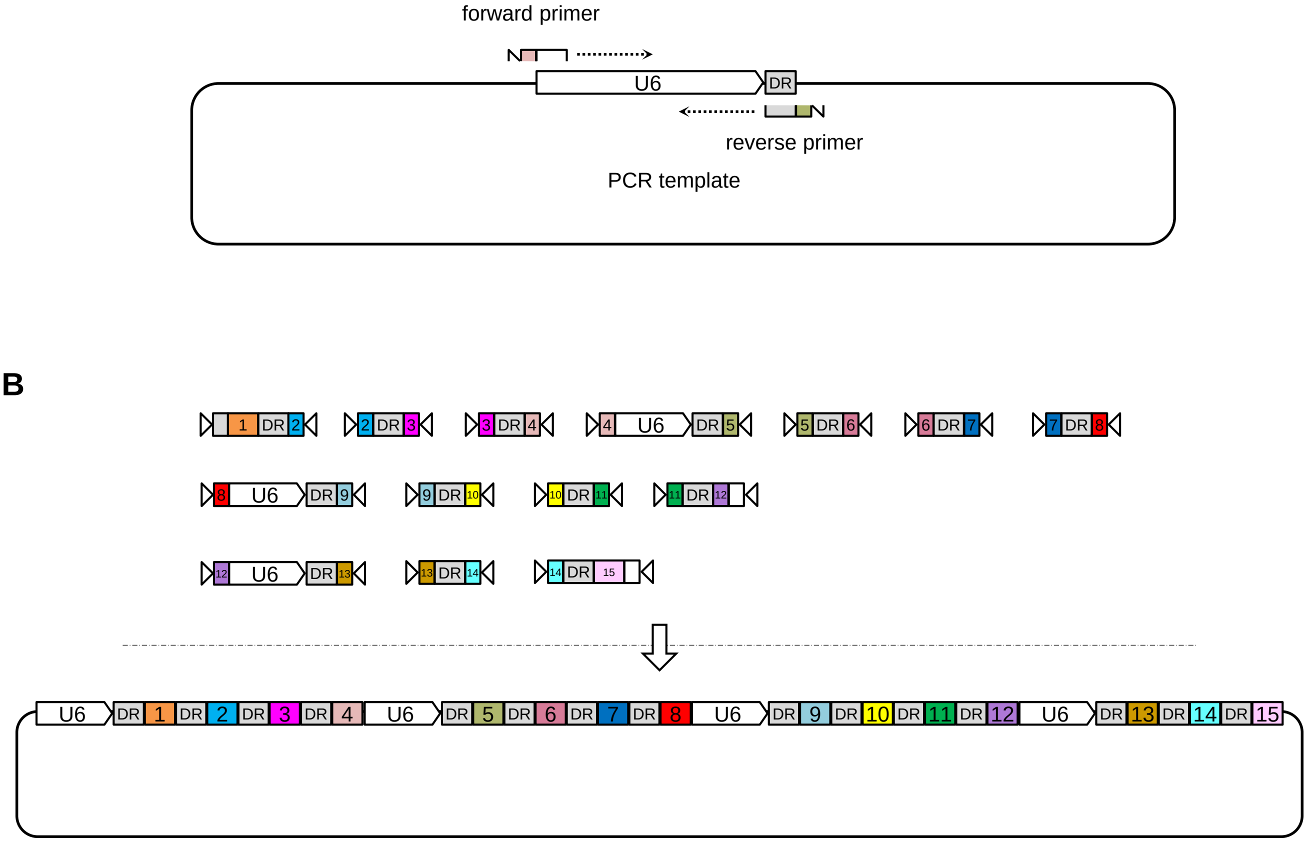

Figure S15. Assumption for the strategy to assemble a hierarchical CRISPR array. (**A**) Schematic illustration of the amplification of U6-containing fragments using a template containing a U6-DR cassette. (**B**) Schematic illustration of the proto assumption to assemble hierarchical CRISPR array.

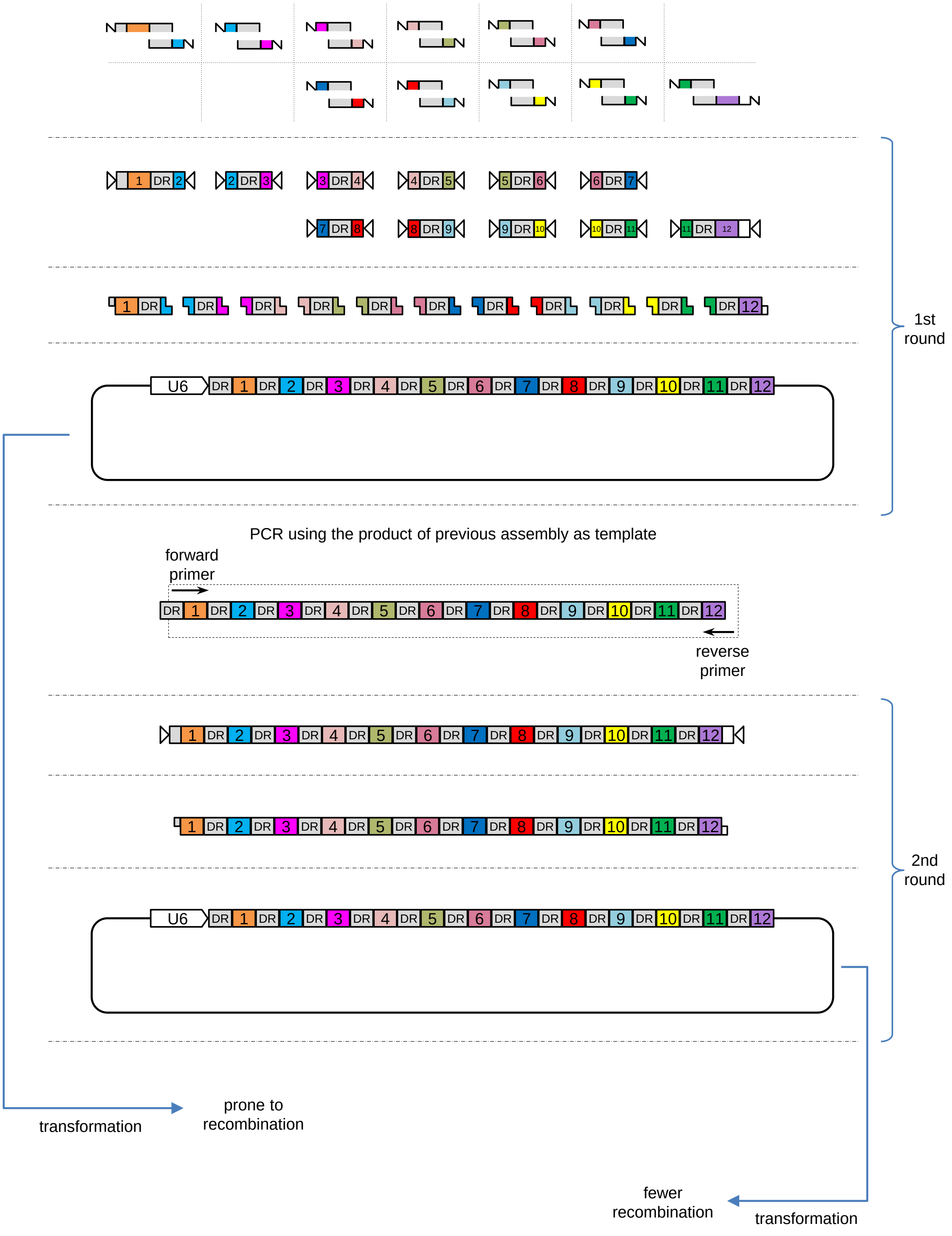

Figure S16. Schematic illustration and workflow of assembling CRISPR array using 2 rounds of assembly.

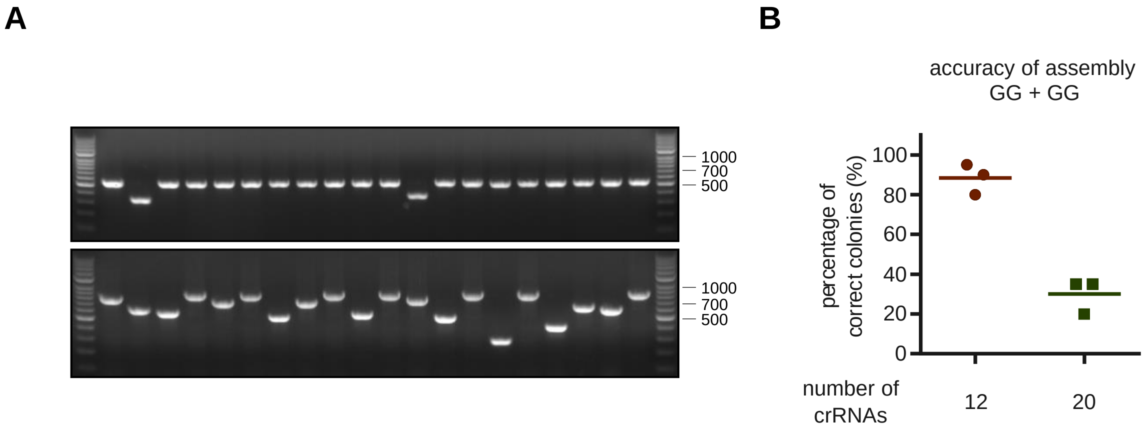

Figure S17. Accuracies of assembling 12 and 20 crRNAs using 2 rounds of assembly. (**A**) Representative images of colony PCR when assembling 12 (upper, correct size ≈ 530 nt) and 20 (lower, correct size ≈ 840 nt) crRNAs, and the corresponding accuracies are shown in (**B**). Note that since we failed to amplify the overall array from the assembly mixture of 20 crRNAs, the second round of PCR was separated into two individual reactions: crRNA 1-10, crRNA 11-20. Values shown as mean with n = 3.

A

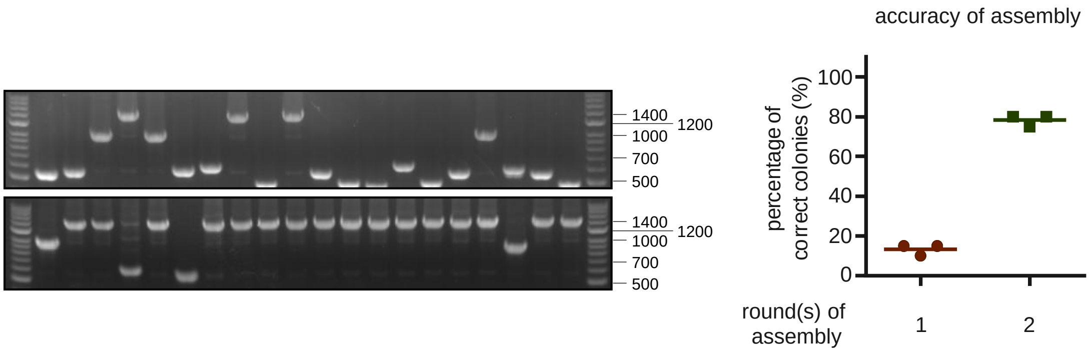

B

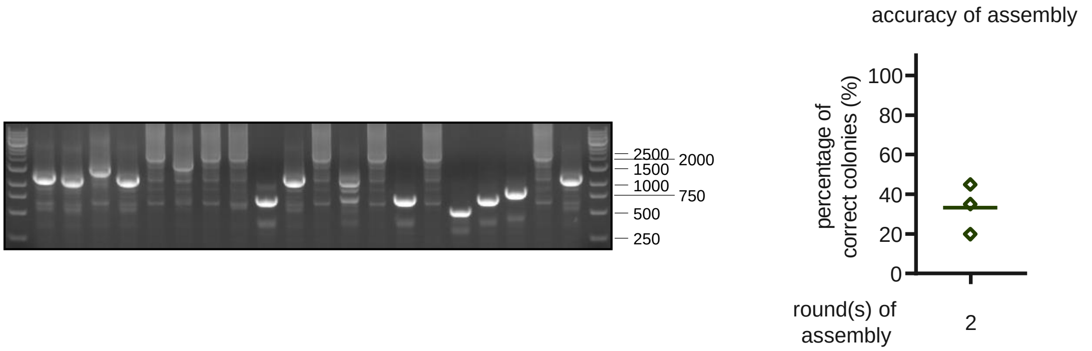

Figure S18. Assembly of complex, hierarchical CRISPR arrays. (**A**) Representative images of colony PCR when assembling an array consisting of 3 U6 promoters and 12 crRNAs (correct size ≈ 1450 nt) , by either a single (upper) or two (lower) rounds of assembly, and the corresponding accuracies are shown in (right). (**B**) Representative image of colony PCR when assembling an array consisting of 4 U6 promoters and 15 crRNAs by two rounds of assembly (correct size ≈ 2240 nt), and the corresponding accuracies are shown in (right). Values shown as mean with n = 3.

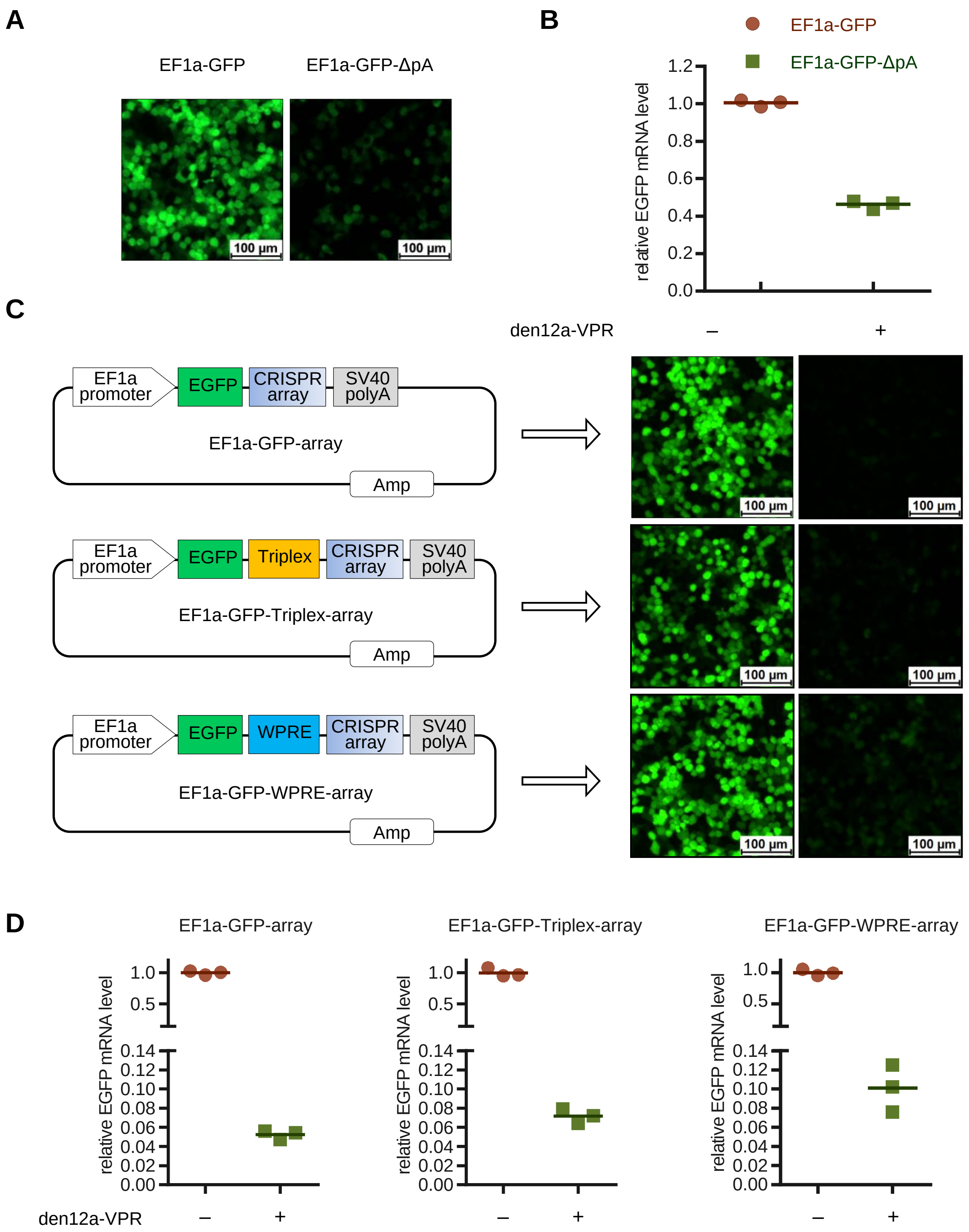

Figure S19. Processing of CRISPR array leads to inadequate expression of upstream EGFP encoded on the same transcript. **(A**, **B)** Representative fluorescence images **(A)** and mRNA expression **(B)** in HEK293T cells 48h after transfection with EF1a-driven EGFP constructs with/without a downstream SV40-poly(A) signal. Scale bar, 100 μm. **(C)** Schematics of EGFP constructs (left) and corresponding representative fluorescence images (right) 48h after transfection together with/without den12a-VPR. Scale bar, 100 μm. **(D)** Quantification of EGFP mRNA expression 48h after transfection with EGFP constructs together with/without den12a-VPR. Values shown as mean with n = 3.

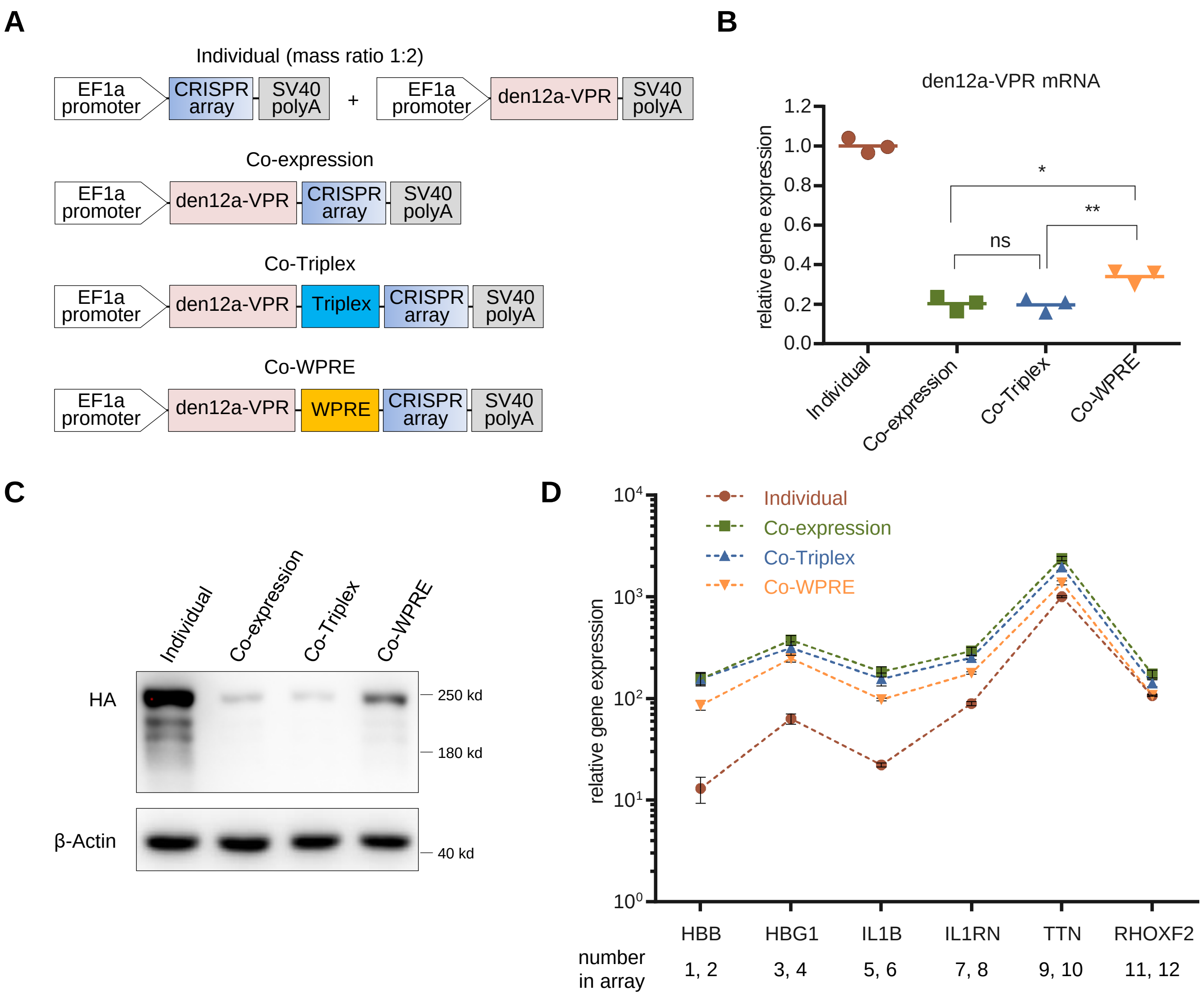

Figure S20. Co-expression of Cas protein and CRISPR array on a single transcript. (**A**) Schematics of constructs used to assess the targeting efficiency of co-expressing Cas protein and CRISPR array on a single transcript. (**B**, **C**) Relative mRNA (**B**) and protein (**C**) expression level of denAsCas12a-VPR in HEK293T cells 48h after transfection with constructs depicted in (**A**). Values shown as mean with n = 3. (**D**) Quantification of relative mRNA expression over non-targeting control in HEK293T cells 48h after transfection with constructs depicted in (**A**). Values shown as mean ± SD with n = 3.

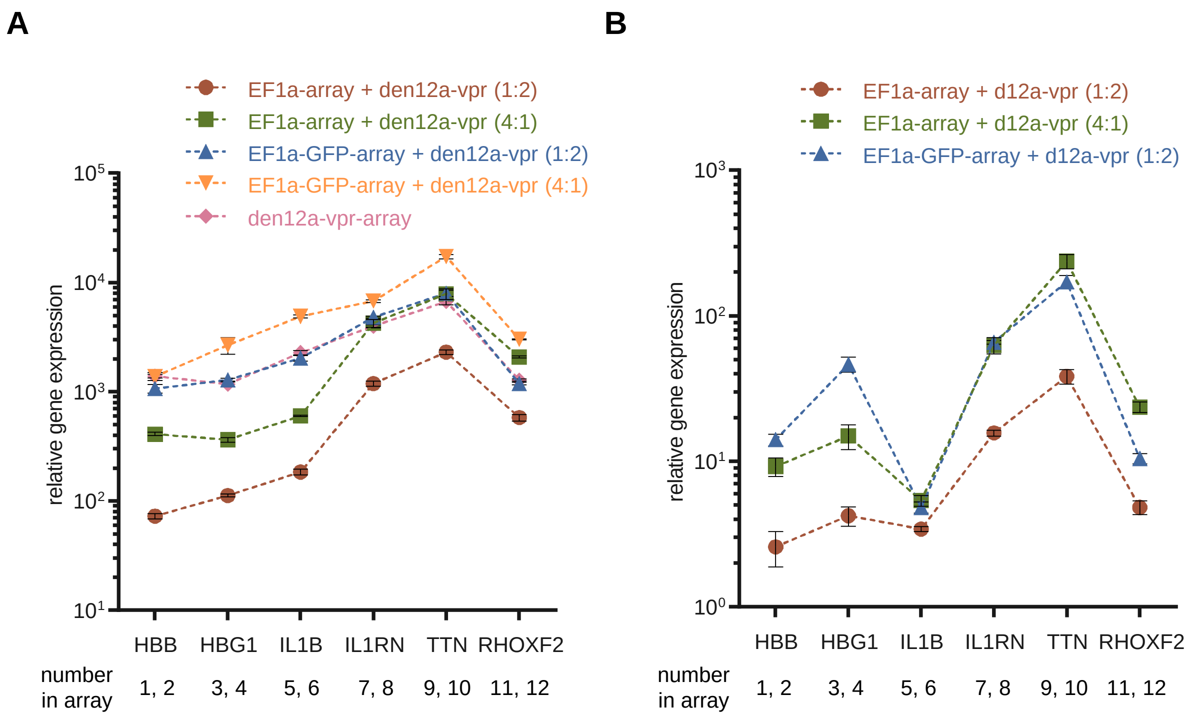

Figure S21. Comparison of the targeting efficiency across different mass ratios or expression patterns. **(A)** Quantification of relative mRNA expression over non-targeting control in HEK293T cells 48h after transfection with the indicated constructs at the mass ratios annotated in parentheses. **(B)** Quantification of relative mRNA expression over non-targeting control in HEK293T cells 48h after transfection with dAsCas12a-VPR and the indicated array-expressing constructs at the mass ratios annotated in parentheses. Values shown as mean ± SD with n = 3.

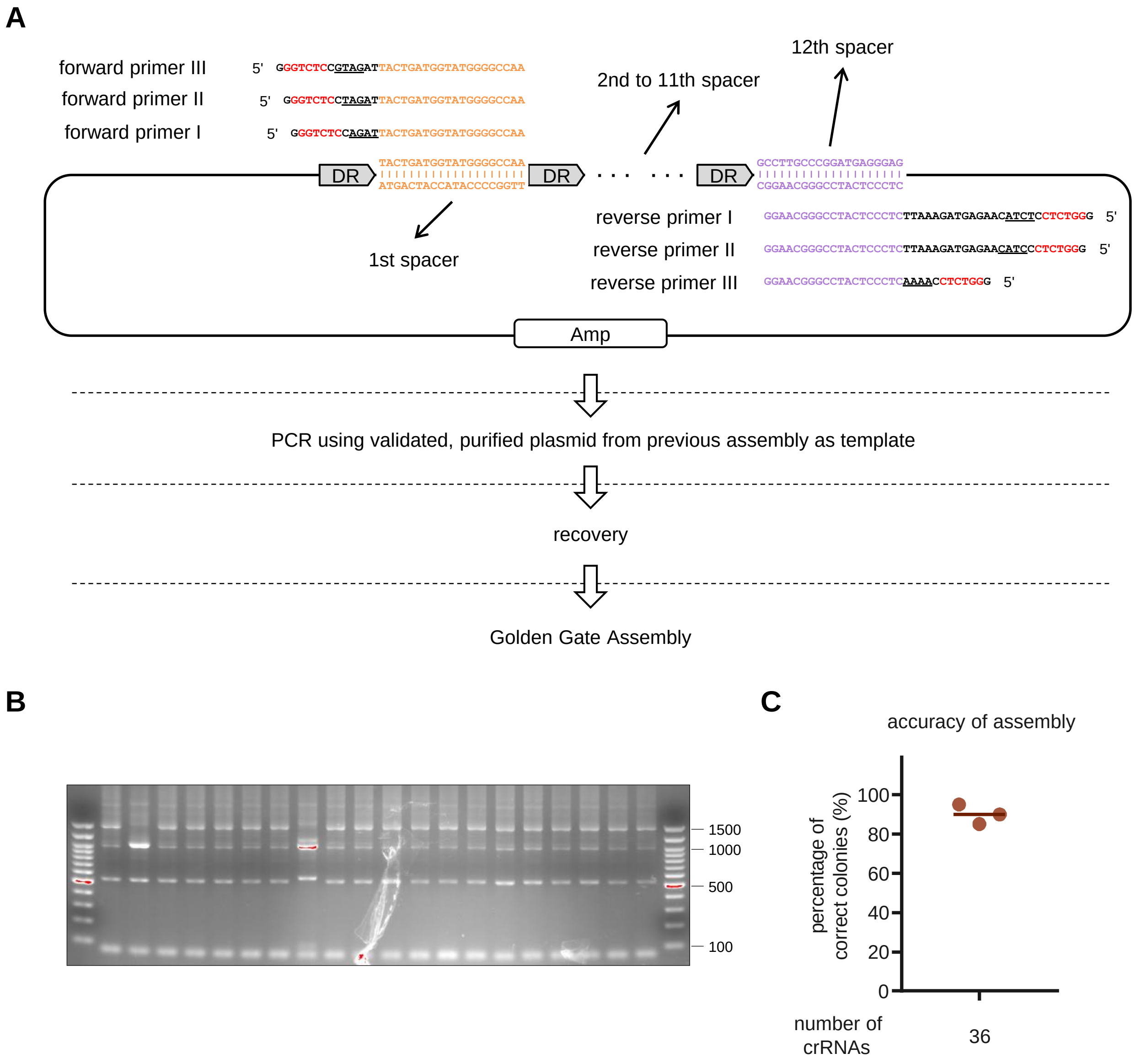

Figure S22. Generation of an array containing 36 crRNAs by two rounds of assembly. **(A)** Schematic illustration and workflow of the second round of assembly for the generation of an array containing 36 crRNAs. A detailed description of the first round of assembly is illustrated in Figure 1 and will not be shown here again. The resulting plasmid from the first round of assembly, encoding an array of 12 crRNAs, is used as a template for the second round of 3 PCR reactions (with primer pair I, II, III, respectively). Recovery the PCR products. Then, set up and run a standard Gold Gate assembly reaction with purified segments and destination cloning vector. **(B)** Representative images of colony PCR to evaluate the accuracy of the second round of assembly (correct size ≈ 1460 nt), and the corresponding accuracy are shown in **(C)**.

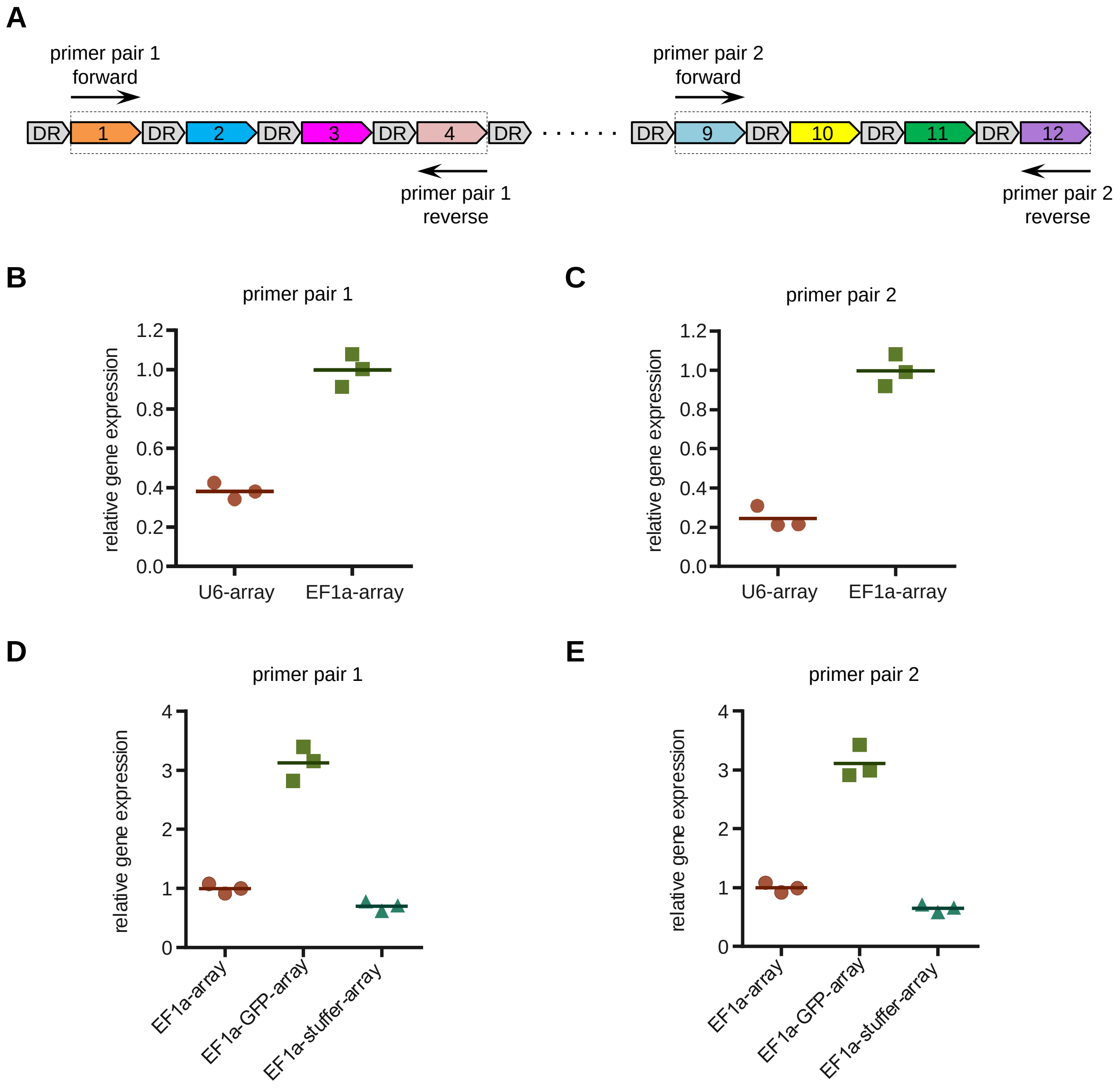

Figure S23. Expression of precursor arrays (for den12a-VPR) driven by Pol II or Pol III promoter. **(A)** Schematics of the two pairs of primers used to quantify the expression of CRISPR arrays. **(B**, **C)** Relative expression of the front (**B**) and back (**C**) parts of the CRISPR arrays driven by either U6 or EF1a. Values shown as mean with n = 3. **(D**, **E)** Relative expression of the front (**D**) and back (**E**) parts of the CRISPR arrays driven by EF1a with or without an upstream GFP or stuffer. Values shown as mean with n = 3.

An amount of 1 μg total RNA was reverse transcribed using ReverTra Ace® qPCR RT Kit (Toyobo) with respective reverse primer show in **Figure A**, followed by qPCR using ChamQ Universal SYBR qPCR Master Mix (Vazyme). qPCR reactions were performed using LightCycler 480 II (Roche). Quantification of RNA expression was normalized to ACTB (unless otherwise specified) and calculated using the ΔΔCt method.

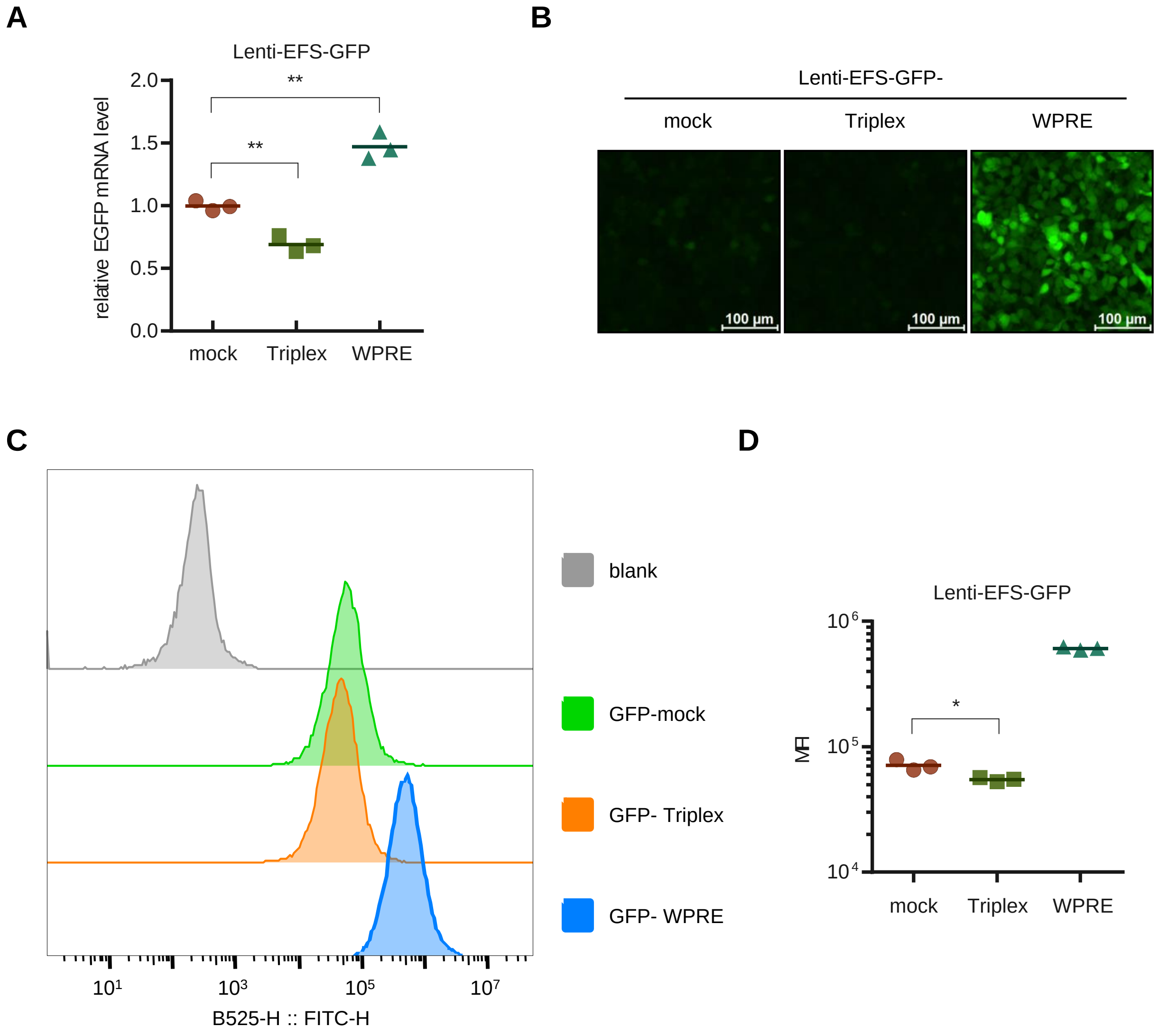

Figure S24. Introduction of WPRE, rather than the "Triplex" element, enhances the expression of gene delivered by lentivirus. **(A-D)** Quantification of EGFP mRNA expression **(A)**, representative fluorescence images **(B)**, representative fluorescence histograms detected by flow cytometry **(C)**, and mean fluorescence intensities **(D)** in HEK293T cells 72h after infection with EGFP-encoding lentivirus, either with or without WPRE or the "Triplex" element. Scale bar, 100 µm. Values shown as mean with n = 3.

Supplemental Methods

Protocol 1: Assembly of CRISPR arrays for (d)(en)AsCas12a with conventional sticky end-based strategies.

Reagents: BsaI-HF v2 (NEB, R3733), T4 DNA Ligase (NEB, M0202).

1) Set up the annealing reactions (total volume of 20 μl for each reaction) in PCR tubes as in **Table S23**.

2) Transfer PCR tubes to a thermocycler and run the annealing program as in **Table S24**.

3) Dilute annealed oligos to a final concentration of 0.5 μM (i.e., 5-fold dilution by adding 80 μl ddH_2O to each reaction).

4) Three different versions of assembly were performed individually as indicated in Figure S4: (-)BsaI, (+)BsaI, Gold Gate.

   4.1) Prepare the assembly reaction as in **Table S25**.

   Incubate at 16 ℃ overnight with a thermocycler.

   4.2) Prepare the assembly reaction as in **Table S26**.

   Incubate at 16 ℃ overnight with a thermocycler, followed by 37 ℃ for 15 min before transformation.

   4.3) Prepare the assembly reaction as in **Table S27**.

   Run the following Golden Gate Assembly program with a thermocycler: (37°C 5 min → 16 ℃ 5 min) × 30 cycles, followed by 60 ℃ for 5 min. If reactions are done overnight, add a 4 ℃ terminal hold to the program and repeat the step of 60 ℃ for 5 min the next day before transformation.

5) Transform 2-10 μl of the assembly reaction into competent cells.

Protocol 2: Assembly of CRISPR arrays for (d)(en)AsCas12a with GGA-based strategy.

Reagents: BsaI-HF v2 (NEB, R3733), T4 DNA Ligase (NEB, M0202), Taq DNA Polymerase (2 × premix), sodium acetate (3M, pH 5.2).

1) Set up the PCR reactions (total volume of 50 μl for each reaction) as in **Table S28**.

2) Transfer PCR tubes to a thermocycler and run a routine PCR program as in **Table S29**.

3) Upon completion, transfer PCR products to 1.5 ml tubes. Add 1/10 volume of NaAc (3M, pH 5.2), and 2 volumes of pre-chilled 100% ethanol. Mix well. Store at -20 ℃ for 20 min to overnight to precipitate the DNA.

4) Centrifuge at 10,000-20,000g for 15 min (at 4 ℃ if convenient). Carefully pour off the ethanol (or remove by pipetting) without disturbing the pellet. Wash twice with 70-75% ethanol. Allow the DNA pellet to air-dry. Dissolve the DNA 20 μl TE buffer or ddH_2O.

5) Quantify the concentration of recovered DNA samples. Adjust them to a uniform concentration (e.g., around 5 ng/μl).

6) Prepare the assembly reaction as in **Table S30**.

7) Run the following Golden Gate Assembly program with a thermocycler: (37°C 5 min → 16°C 5 min) × 30-60 cycles, followed by 60 ℃ for 5 min. If reactions are done overnight, add a 4 ℃ terminal hold to the program and repeat the step of 60 ℃ for 5 min the next day before transformation.

8) Transform 2-10 μl of the assembly reaction into competent cells.

Protocol 3: A simplified version of Protocol 2.

Reagents: BsaI-HF v2 (NEB, R3733), T4 DNA Ligase (NEB, M0202), Taq DNA Polymerase (2 × premix).

Note: The optimal primer concentration for PCR reaction and the volume pipetted for subsequent assembly were determined based on the designated PCR reagent used in this study. Although these reaction parameters may need to be optimized for different PCR reagents, they can be used as benchmarks. When necessary, adjust them up or down to obtain satisfactory results.

1) Set up the PCR reactions (total volume of 20 μl for each reaction) as in **Table S31**.

2) Transfer PCR tubes to a thermocycler and run a routine PCR program as in **Table S32**.

3) Upon completion, dilute PCR products 10-fold by adding 180 μl ddH_2O to each reaction.

4) Prepare the assembly reaction as in **Table S33**.

5) Run the following Golden Gate Assembly program with a thermocycler: (37°C 5 min → 16°C 5 min) × 30-60 cycles, followed by 60 ℃ for 5 min. If reactions are done overnight, add a 4 ℃ terminal hold to the program and repeat the step of 60 ℃ for 5 min the next day before transformation.

6) Transform 2-10 μl of the assembly reaction into competent cells.

Protocol 4: Assembly of CRISPR arrays for RfxCas13d with GGA-based strategy.

Reagents: BsaI-HF v2 (NEB, R3733), T4 DNA Ligase (NEB, M0202), PrimeSTAR HS DNA Polymerase (Takara, R010A).

1) Set up the PCR reactions (total volume of 50 µl for each reaction) as in **Table S34**.
2) Transfer PCR tubes to a thermocycler and run a routine PCR program as in **Table S35**.
3) Upon completion, purify PCR segments with available DNA recovery kit. Quantify the concentration. Adjust them to a uniform concentration (e.g., around 5 ng/µl).
4) Prepare the assembly reaction as in **Table S36**.
5) Run the following Golden Gate Assembly program with a thermocycler: (37°C 5 min → 16°C 5 min) × 30-60 cycles, followed by 60 ℃ for 5 min. If reactions are done overnight, add a 4 ℃ terminal hold to the program and repeat the step of 60 ℃ for 5 min the next day before transformation.
6) Transform 2-10 µl of the assembly reaction into competent cells.

Supplemental Tables

Table S1. Primers used for qPCR.

Table S2. Gene-specific primers used for reverse transcription (also used as the reverse primer of subsequent qPCR) and counterpart qPCR primers.

Table S3. Spacer sequences for transcriptional activation mediated by dAsCas12a-VPR or denAsCas12a-VPR.

Table S4. Spacer sequences for RfxCas13d-mediated RNA cleavage.

Table S5. Spacer sequence for gene editing mediated by enAsCas12a.

Table S6. Primers used for the amplification and sequencing of the target region flanking indels.

Table S7. Primers used to assemble the CRISPR array of 6 crRNAs for dAsCas12a-VPR or denAsCas12a-VPR with conventional sticky end-based strategies.

Table S8. Primers used to assemble the CRISPR array of 7 crRNAs for dAsCas12a-VPR or denAsCas12a-VPR with conventional sticky end-based strategies.

Table S9. Primers used to assemble the CRISPR array of 6 crRNAs for dAsCas12a-VPR or denAsCas12a-VPR with GGA-based strategy.

Table S10. Primers used to assemble the CRISPR array of 7 crRNAs for dAsCas12a-VPR or denAsCas12a-VPR with GGA-based strategy.

Table S11. Primers used to assemble the CRISPR array of 9 crRNAs for dAsCas12a-VPR or denAsCas12a-VPR with GGA-based strategy.

Table S12. Primers used to assemble the CRISPR array of 12 crRNAs for dAsCas12a-VPR or denAsCas12a-VPR with GGA-based strategy.

Table S13. Primers used to assemble 12 crRNAs for dAsCas12a-VPR or denAsCas12a-VPR based on mutant DR.

Table S14. Primers used to assemble the CRISPR array of 9 crRNAs for RfxCas13d with GGA-based strategy.

Table S15. Primers used to assemble the CRISPR array of 12 crRNAs for RfxCas13d with GGA-based strategy.

Table S16. Primers used to assemble the CRISPR array of 15 crRNAs for RfxCas13d with GGA-based strategy.

Table S17. Primers used to assemble 12 crRNAs for dAsCas12a-VPR or denAsCas12a-VPR with two rounds of assembly.

Table S18. Primers used to assemble 20 crRNAs for dAsCas12a-VPR or denAsCas12a-VPR with two rounds of assembly.

Table S19. Primers used to assemble U6-4-U6-4-U6-4 for dAsCas12a-VPR or denAsCas12a-VPR with two rounds of assembly.

Table S20. Primers used to assemble U6-4-U6-4-U6-4-U6-3 for RfxCas13d with two rounds of assembly.

Table S21. Sequences of 5’ bioton labelled DNA probes used for Northern blots of mature crRNAs.

Table S22. Sequences of constructs used in this study.

Table S23. Corresponding to step 1) of **Protocol 1**.

component	volume	final concentration
forward primer (10 μM)	5 μl	2.5 μM
reverse primer (10 μM)	5 μl	2.5 μM
T4 DNA Ligase Buffer (10×)	2 μl	1×
ddH_2O	8 μl	

Table S24. Corresponding to step 2) of **Protocol 1**.

step	temperature	duration	cycles
1	95℃	4 min	
2	70℃	10 min	
3	70℃ (set: -0.1℃/cycle)	2 sec	
4	go to step 3		500
end			

Table S25. Corresponding to step 4.1) of **Protocol 1**.

component	volume/amount
diluted annealed oligos (0.5 μM)	1 μl each
recovered predigested destination vector	30-80 ng
T4 DNA Ligase Buffer (10×)	2 μl
T4 DNA Ligase	0.5 μl
ddH_2O	to 20 μl

Table S26. Corresponding to step 4.2) of **Protocol 1**.

component	volume/amount
diluted annealed oligos (0.5 μM)	1 μl each
recovered predigested destination vector	30-80 ng
T4 DNA Ligase Buffer (10×)	2 μl
BsaI-HF v2	1 μl
T4 DNA Ligase	0.5 μl
ddH_2O	to 20 μl

Table S27. Corresponding to step 4.3) of **Protocol 1**.

component	volume/amount
diluted annealed oligos (0.5 μM)	1 μl each
destination vector	30-80 ng
T4 DNA Ligase Buffer (10×)	2 μl
BsaI-HF v2	1 μl
T4 DNA Ligase	0.5 μl
ddH_2O	to 20 μl

Table S28. Corresponding to step 1) of **Protocol 2**.

component	volume	final concentration
forward primer (10 μM)	5 μl	1 μM
reverse primer (10 μM)	5 μl	1 μM
Taq premix (2×)	25 μl	1×
ddH_2O	15 μl	

Table S29. Corresponding to step 2) of **Protocol 2**.

step	temperature	duration	cycles
1	95℃	3 min	
2	95℃	30 sec	
3	45-50℃	30 sec	
4	72℃	30 sec	
5	go to step 2		34
6	72℃	5 min	

Table S30. Corresponding to step 6) of **Protocol 2**.

component	volume/amount
recovered PCR segments	~5 ng each
destination vector	30-80 ng
T4 DNA Ligase Buffer (10×)	2 μl
BsaI-HF v2	1-2 μl
T4 DNA Ligase	0.5 μl
ddH_2O	to 20 μl

Table S31. Corresponding to step 1) of **Protocol 3**.

component	volume	final concentration
forward primer (10 μM)	5 μl	2.5 μM
reverse primer (10 μM)	5 μl	2.5 μM
Taq premix (2×)	10 μl	1×

Table S32. Corresponding to step 2) of **Protocol 3**.

step	temperature	duration	cycles
1	95℃	3 min	
2	95℃	30 sec	
3	45-50℃	30 sec	
4	72℃	30 sec	
5	go to step 2		34
6	72℃	5 min	

Table S33. Corresponding to step 4) of **Protocol 3**.

component	volume/amount
diluted PCR products	1 μl each
destination vector	30-80 ng
T4 DNA Ligase Buffer (10×)	2 μl
BsaI-HF v2	1-2 μl
T4 DNA Ligase	0.5 μl
ddH_2O	to 20 μl

Table S34. Corresponding to step 1) of **Protocol 4**.

component	volume/amount	final concentration
5 ×Buffer (Mg^{2+} Plus)	10 μl	1 ×
dNTP mixture (2.5 mM each)	4 μl	200 μM each
DNA polymerase	0.5 μl	
forward primer (10 μM)	5 μl	1 μM
reverse primer (10 μM)	5 μl	1 μM
template plasmid	0.2-1 ng	
ddH_2O	to 50 μl	

Table S35. Corresponding to step 2) of **Protocol 4**.

step	Temperature	duration	cycles
1	98℃	30 sec	
2	98℃	10 sec	
3	50℃	15 sec	
4	72℃	30 sec	
5	go to step 2		34
6	72℃	5 min	

Table S36. Corresponding to step 4) of **Protocol 4**.

component	volume/amount
recovered PCR segments	~5 ng each
destination vector	30-80 ng
T4 DNA Ligase Buffer (10×)	2 µl
BsaI-HF v2	1-2 µl
T4 DNA Ligase	0.5 µl
ddH_2O	to 20 µl

Table S37. Sanger sequencing results in FASTA format of correctly sized colonies.

Table S38. Sanger sequencing results in FASTA format of incorrectly sized colonies.
